# Global burden and trends of occupational noise-induced hearing loss (1990–2021) and projection to 2040

**DOI:** 10.3389/fpubh.2025.1682413

**Published:** 2025-09-22

**Authors:** Xin Gong, Meitao Yi, Cuiping Jiang, Qiao Xiong, Bingying Xu, Falin Weng, Lingna Zeng, Rumei Lu, Zhenglong Chen, Chuanjiang Yan, Qingqing Li, Qiang Zhang

**Affiliations:** ^1^Department of Otolaryngology, Wushan County People’s Hospital of Chongqing, Wushan, Chongqing, China; ^2^Department of Operating Rooms, Wushan County People's Hospital of Chongqing, Wushan, Chongqing, China; ^3^Department of Pediatrics, Wushan County People's Hospital of Chongqing, Wushan, Chongqing, China; ^4^Department of Geriatric Medicine, Wushan County People's Hospital of Chongqing, Wushan, Chongqing, China; ^5^Department of Radiology, Wushan County People's Hospital of Chongqing, Wushan, Chongqing, China; ^6^Department of Spinal Surgery, Wushan County People's Hospital of Chongqing, Wushan, Chongqing, China; ^7^Mental Health Center, Wushan County People's Hospital of Chongqing, Wushan, Chongqing, China

**Keywords:** noise-induced hearing loss, occupational noise, DALYs, summary exposure values, health inequality, trends, Global Burden of Disease

## Abstract

**Background:**

Occupational noise is a global issue that widely affects workers’ physical health and quality of life. This study aimed to illustrate the trends and spatiotemporal patterns of occupational noise-induced hearing loss (ONIHL) burden from 1990 to 2021 and project future trends.

**Methods:**

Utilizing the Global Burden of Disease Study (GBD) 2021 data, we calculated ONIHL disability-adjusted life years (DALYs), age-standardized DALY rates (ASDRs), and summary exposure values (SEVs) by age, sex, and the Socio-demographic Index (SDI). Inequality and decomposition analyses were used to quantify health inequalities and identify the drivers of the ONIHL burden, respectively. The autoregressive integrated moving average (ARIMA) model was used to project the disease burden until 2040.

**Results:**

In 2021, the global burden of ONIHL remained substantial, with a total of 7,847,444.59 DALYs (95% uncertainty intervals [UI]: 5,313,648.10–10,980,789.34), indicating a 104.46% increase compared with that in 1990. The ASDR for ONIHL in 2021 was 91.12 per 100,000 individuals (95% UI: 61.98–127.20). The ASDRs and SEVs showed remarkable growth in high-middle SDI regions, especially for females. Regionally, East Asia, South Asia, Southeast Asia, and Eastern Sub-Saharan Africa experienced the greatest ONIHL burden in the world. Spearman correlation analysis revealed a significant negative correlation between the ASDR and SDI across 21 GBD regions and 204 countries and territories. ONIHL DALYs occurred mainly in middle-aged and older adults, and men consistently presented higher DALYs and ASDRs than females. The ONIHL burden was greater in lower-SDI regions, but cross-country health inequalities did not improve. Decomposition analysis revealed population growth as the main driver. By 2040, ONIHL DALYs are predicted to increase, whereas the ASDR is projected to decrease; however, the disease burden among females will rise significantly.

**Conclusion:**

The ONIHL burden is characterized by global growth, regional divergence, and widening disparities in sex trends. Targeted actions like bolstering occupational safety in lower SDI regions, adopting gender-responsive policies for female workers in high-noise industries, and prioritizing early hearing screening and long-term monitoring of noise hazards are key to tackling the burden of ONIHL worldwide.

## 1 Introduction

Hearing loss (HL), the most prevalent disability affecting sensory organs, is a major global health concern, affecting more than one-fifth of the global population, with at least 700 million individuals requiring rehabilitation services (1, 2). Noise-induced hearing loss (NIHL) is the second most common cause of HL after age-related hearing loss (ARHL) in adults, and approximately 5% of the global population has NIHL (3, 4). Among these, occupational noise-induced hearing loss (ONIHL) refers to sensorineural HL caused by long-term exposure to occupational noise where the 8-h equivalent sound level exceeds 85 decibels [dB(A)] (5). Occupational noise, a key modifiable risk factor, accounted for 16.87% of total disability-adjusted life years (DALYs) for HL in 2019 (6). Studies have shown that approximately 22 million people in the U. S. industries are affected by hazardous noise exposure (7). In China, 88.8% of enterprises had noise hazards in 2020, and an estimated 80 million out of a total of 574 million workers in the country’s industrial and service sectors were exposed to such hazardous noise (8). ONIHL is associated with tinnitus, cardiovascular ailments, sleep disturbances, and diminished performance (9–11), and it not only impairs an individual’s quality of life, hinders communication, and is linked to various emotional and cognitive problems (such as depression and cognitive deterioration) but also imposes substantial socioeconomic burdens, including reduced productivity and increased healthcare costs (12–15).

From a pathological perspective, outer hair cells are generally more susceptible to damage and usually sustain greater numerical losses compared to inner hair cells. Specifically, when these cells are impaired, it results in a decrease in cochlear amplification, which eventually causes an elevation in hearing threshold. Furthermore, long-term exposure to intense noise can cause synaptic impairment and degeneration of spiral ganglion neurons while also damaging the stria vascularis, which leads to a decrease in blood flow and, together with the former, leads to threshold elevation (16–20). Regrettably, the condition remains irreversible once permanent hearing damage resulting from occupational noise exposure occurs, yet the ONIHL burden is continuing to grow (5). To adhere to national regulations on noise exposure limits, employers are obligated to strengthen workplace noise management and control, ensuring that noise levels in all work areas comply with legal standards—a measure critical to protecting employees’ hearing health and maintaining workplace safety. Notably, occupational noise intensity and duration are key factors contributing to ONIHL. In reality, however, many enterprises fail to fulfill this obligation: they allow excessive noise levels, subject workers to prolonged, high-intensity work, exercise lax supervision, and provide inadequate training on occupational noise exposure, and these practices leave workers without effective protection (21, 22). Certainly, workers’ lack of occupational protection awareness is also a reason (23). Given that ONIHL causes irreversible hearing damage, its burden continues to grow, and practical prevention and control efforts remain inadequate due to enterprise non-compliance and low worker awareness. Therefore, using the newly released Global Burden of Disease study (GBD) 2021 data to understand the latest burden and trends of ONIHL is of great importance for formulating relevant policies.

Prior studies have explored the worldwide epidemiology of ONIHL utilizing data from GBD-based studies, including GBD 2000, GBD 2017, and GBD 2019 (6, 24–26). Nevertheless, compared with the latest GBD 2021 data used in this study, these studies have certain limitations. First, as explicitly stated in GBD 2021, newly released employment data from the International Labour Organisation (ILO) were included (27)—leading to significant discrepancies from previous research, particularly in the values and trends of ONIHL burden. Yet GBD 2021’s latest methodologies and data are likely to be more closely aligned with the actual burden. For example, GBD 2019 showed a decreasing trend in the ASDR (from 85.70 in 1990 to 84.23 in 2019) (6), whereas GBD 2021 indicated an increasing trend (from 84.28 in 1990 to 91.12 in 2021). Second, building on previous descriptive studies, this research incorporates health inequality analysis, decomposition analysis, frontier analysis, and autoregressive integrated moving average (ARIMA) model predictive analysis that help to shed light on health inequalities in disease burden among countries at different income levels, key factors driving regional disease burden changes, achievable disease burden control levels across countries, and future disease burden trends. This bridges existing research gaps and enables a more intuitive and in-depth exploration of the trends and spatiotemporal patterns of the ONIHL burden. These findings aim to underpin evidence-driven strategies for preventing and controlling the ONIHL burden, particularly in specific regions and vulnerable populations, and provide pivotal insights for public health planning endeavors.

## 2 Methods

### 2.1 Overview

All data on the ONIHL burden were derived from GBD 2021, a comprehensive study that is maintained by the Institute for Health Metrics and Evaluation (IHME) at the University of Washington and systematically synthesizes the non-fatal burden of 371 diseases and injuries and 88 risk factors across 204 countries and territories (28). DisMod-MR 2.1, a Bayesian mixed-effects meta-regression modeling tool developed for GBD analyses, is designed to estimate non-fatal health outcomes using sparse and heterogeneous epidemiological data and provides a comprehensive view of diseases. Further information is available on the IHME website at https://vizhub.healthdata.org/gbd-results/.

### 2.2 Definition and data sources

The GBD study defines HL as a hearing loss greater than 20 dB in the better ear, measured as the pure-tone average (PTA) of four frequencies (0.5, 1, 2, and 4 kHz) (29). Age-related hearing loss and other hearing loss—Level 3 causes, which include causes not identified as meningitis, chronic otitis media, or congenital, have occupational noise as one of their contributing factors (30). Occupational noise is defined in the GBD as level 3 risk, referring to the proportion of the population ever occupationally exposed to 85 + dB of noise, on the basis of population distributions across 17 economic activities (27). Although not directly defined, according to GBD’s classification methodology, the age-related hearing loss and other hearing loss caused by occupational noise refers to ONIHL. The summary exposure value (SEV) of occupational noise was calculated as the weighted average of noise exposure levels across all workers, employing a relative risk function that captures the relationship between noise exposure and HL. In our data search, we used ‘age-related and other hearing loss’ as the cause, whereas ‘occupational noise’ served as the risk factor under investigation. We measured ‘DALYs (Disability-Adjusted Life Years)’, the age-standardized DALY rate (ASDR) and the SEV from 1990 to 2021, utilizing metrics such as number and rate. ASDR and SEV were computed via the GBD 2021 global population age standard. Data on the number of DALYs, ASDRs, and SEVs and their 95% uncertainty intervals (UI) were collected and further analyzed by sex, age, region, and country. We categorized the world into 21 regions on the basis of epidemiological similarities and geographic proximity. For age classification, individuals aged 15 to 95+ years were grouped into 17 categories at 5-year intervals.

The Socio-demographic Index (SDI) serves as a measure of a country’s socio-economic standing, where higher values correspond to more advanced socio-economic development. Derived from national indicators—including per capita income, average educational attainment, and total fertility rate—this index ranges from 0 to 1. GBD 2021 classifies 204 countries and territories into five development levels by SDI: low (≤0.4658), low-middle (0.4658–0.6188), middle (0.6188–0.7120), high-middle (0.7120–0.8103), and high (>0.8103) (31).

### 2.3 Estimation of disease burden

To assess and compare DALY rates across countries or regions with differing age structures and demographic features, the ASDR was utilized. For a clearer understanding of the temporal trends in the ONIHL burden, the estimated annual percentage changes (EAPCs) were computed. A regression line was applied to the natural logarithm of the ASDR, following the equation:


y=α+βx+e,

where *y* represents ln (ASDR) and x represents the calendar year. The EAPC, along with its 95% confidence interval (CI), was then calculated via the formula.


EAPC=100×(exp[β]−1),

with β indicating the slope of the log-linear regression model (32). If the 95% CI of the EAPC is greater than 0, it indicates an upward trend in the ASDR; if it is less than 0, it indicates a downward trend. If the CI includes 0, the indicator remains stable over time (33). Spearman correlation was employed to assess the association between the ONIHL burden and the SDI.

### 2.4 Cross-country inequality analysis

Cross-country inequality analysis was performed to assess the absolute and relative inequalities in the disease burden of ONIHL by calculating the slope index of inequality (SII) and the concentration index (CI) (34). The SII functions as a measure to quantify absolute inequality in a health indicator between the most and least privileged subgroups of a population, with the entire distribution of socioeconomic variables like education or wealth factored in through a weighted regression model. In contrast, the CI quantifies relative inequality by demonstrating how much a health indicator clusters among disadvantaged or advantaged groups. The SII was calculated via regression of the country-level ASDRs due to ONIHL across all age groups on the sociodemographic development-related relative position scale, defined by the midpoint of the cumulative class range of the population ranked by the SDI. In the estimation of the SII, a robust linear model (RLM) was employed to mitigate the impact of outliers and violations of the homoscedasticity assumption on the results. The CI was calculated by fitting a Lorenz concentration curve to the observed cumulative relative distribution of the populations ranked by the SDI and the ASDRs of disease, as well as numerically integrating the area under the curve. A negative SII or CI represents that a higher SDI corresponds to a lower ASDR, and vice versa. A larger absolute value of the SII or CI indicates greater inequality.

### 2.5 Decomposition analysis

Decomposition analysis is a statistical approach that dissects an overall change into the contributions of various factors, aiming to identify which factors exert a significant effect on the change and quantify the extent of their influence (35). We utilized the Das Gupta decomposition method to decompose changes in ONIHL DALYs from 1990 to 2021 into contributions from aging, population growth, and epidemiological changes. This method allowed us to break down the overall changes in burden into these key factors, providing a clearer understanding of how demographic and epidemiological changes have influenced trends over time. Through analysis of these trends, we obtained a clearer understanding of the underlying or major drivers behind changes in the global burden of ONIHL.

### 2.6 Frontier analysis

To assess the relationship between the ONIHL burden and sociodemographic development levels, we employed frontier analysis to construct an ASDR-based frontier model using the SDI (36). Frontier analysis, which focuses on determining the theoretically lowest ASDR value that each country or territory could achieve on the basis of its current development level (serving as a benchmark for optimal performance), quantifies the gap between a country’s or territory’s current burden and its potential minimum, highlighting areas for improvement. They were used as a benchmark for optimal performance to evaluate the relative efficiency in health outcomes among different countries and regions. Different smoothing spans (0.3, 0.4, 0.5) were used to generate the smooth frontier lines in terms of estimating the non-linear relationship between SDI and ASDR of ONIHL, by combining locally weighted regression (LOESS) with local polynomial regression. To guarantee the robustness and reliability of our findings, we performed 1,000 bootstrap resampling iterations and computed the mean ASDR for each SDI value, thereby effectively accounting for data fluctuations. By quantifying the absolute distance between each country’s or territory’s 2021 ASDR and the frontier line (namely, efficiency difference), we evaluated the improvement potential of each country or territory.

### 2.7 ARIMA model projection

The ARIMA model was developed to predict the trend of the ONIHL burden from 2022 to 2040, utilizing data on DALYs for ONIHL from 1990 to 2021. Prior studies have demonstrated that the ARIMA model is effective for predicting incidence and DALY burdens across a range of conditions, including chronic diseases like Alzheimer’s disease and dementia, as well as acute diseases such as intracerebral hemorrhage (37, 38). The ARIMA model is typically denoted as ARIMA (p, d, q), where p = autoregressive order, d = differencing for stationarity, and q = moving average order (39). This model effectively captures patterns and seasonal fluctuations in time series data by integrating three key components: autoregression, differencing, and moving average. We selected the ARIMA model over alternative forecasting approaches due to its robust performance in capturing long-term trends and irregular fluctuations in epidemiological data with limited prior assumptions, a strength consistently demonstrated in comparative studies on disease burden projection (40).

### 2.8 Statistical analysis

All analyses were performed using R software (version 4.3.3). Statistical significance was set at *p* < 0.05. Our study was carried out in compliance with the Guidelines for Accurate and Transparent Health Estimates Reporting (GATHER) (41).

## 3 Results

### 3.1 Global level

**Figure 1:**
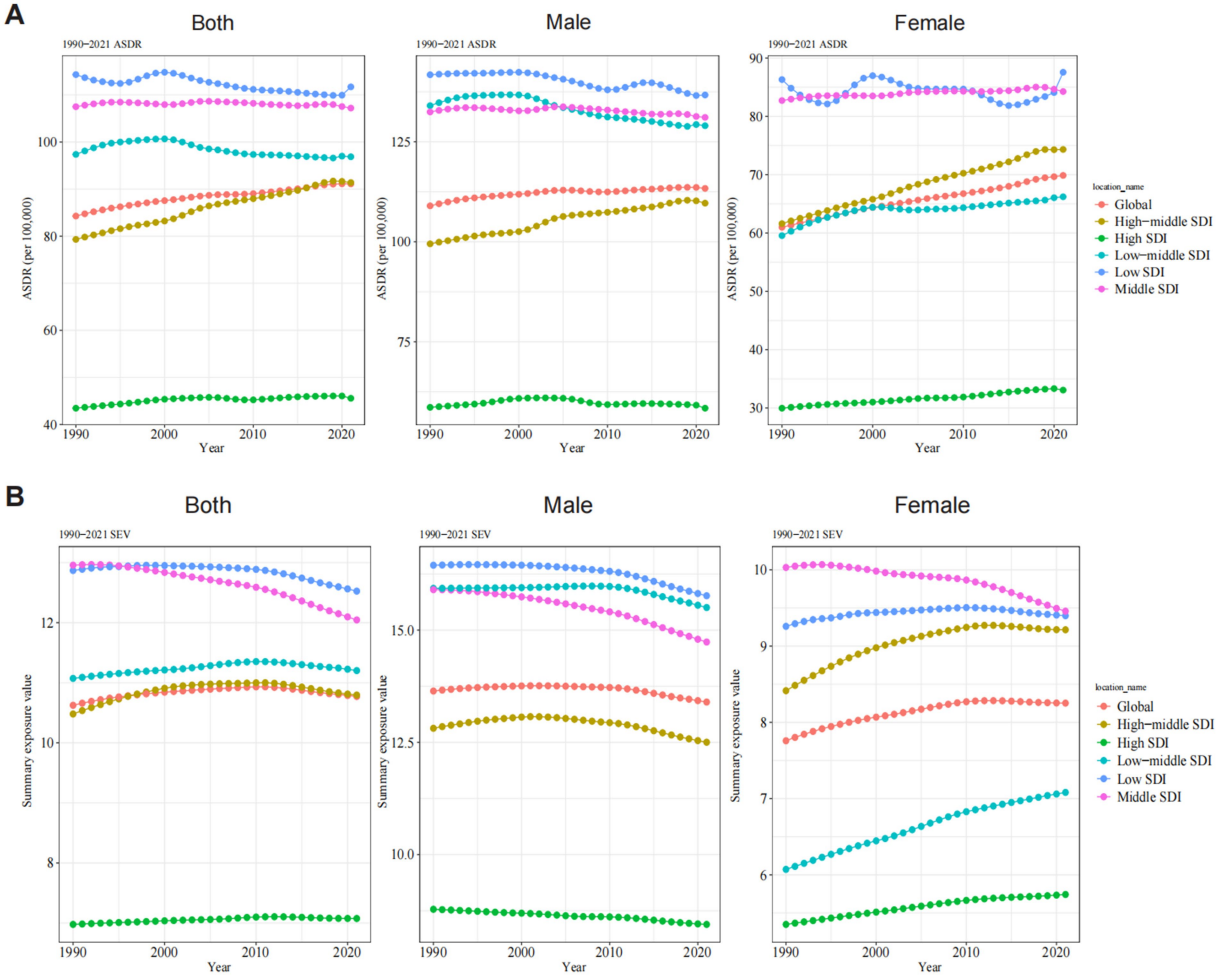
Trends of ASDR **(A)** and SEV **(B)** for ONIHL by sex and SDI stratification from 1990 to 2021. ASDR, age-standardized DALY rate; SEV, summary exposure value; SDI, Socio-demographic Index; ONIHL, occupational noise-induced hearing loss.

In 2021, the global burden of ONIHL remained substantial, with a total of 7,847,444.59 DALYs (95% UI: 5,313,648.10–10,980,789.34), indicating a 104.46% increase compared with that in 1990 (3,838,055.31, 95% UI: 2,630,898.71–5,373,293.46) (Table 1). From 1990 to 2021, the ASDR of ONIHL increased from 84.28 (95% UI: 57.62–118.17) per 100,000 individuals in 1990 to 91.12 (95% UI: 61.98–127.20) per 100,000 in 2021 globally, with an EAPC of 0.23 (95% CI: 0.21 to 0.25) (Table 1 and Figure 1A). In 2021, the estimated global DALYs of ONIHL for males and females were 4,779,974.99 (95% UI: 3,229,198.87–6,666,481.25) and 3,067,469.60 (95% UI: 2,087,476.66–4,314,308.09), respectively. The ASDR for males was greater than that for females (male: 113.35 per 100,000; 95% UI: 76.93–157.86; female: 69.87 per 100,000; 95% UI: 47.67–97.95), and the ratio of ASDR in males to females was approximately 1.6: 1. Notably, from 1990 to 2021, the EAPC of the ASDR in females was 0.42 (95% CI: 0.41 to 0.43), which was significantly greater than that in males (0.11, 95% CI: 0.09 to 0.12) (Table 1 and Figure 1A). In general, the occupational noise-related SEV was 10.77% (95% UI: 10.36–11.34%) in 2021, slightly higher than the 1990 level, with males consistently having higher exposure levels than females. However, SEV showed distinct gender trends: the global increase was driven mainly by increased occupational noise-related SEV among females, from 7.76% (95% UI: 7.45–8.22%) in 1990 to 8.25% (95% UI: 7.93–8.73%) in 2021 (Supplementary Table S1 and Figure 1B).

**Table 1:** Global and regional trends of occupational noise-induced hearing loss burden (DALYs, by 1990–2021).

Location	1990		2021		1990–2021
	Number of DALYs, (95% UI)	ASDR per 100,000, (95% UI)	Number of DALYs, (95% UI)	ASDR per 100,000, (95% UI)	EAPC, %, (95% CI)
Global	3,838,055.31 (2,630,898.71 – 5,373,293.46)	84.28 (57.62–118.17)	7,847,444.59 (5,313,648.10 – 10,980,789.34)	91.12 (61.98–127.20)	0.23 (0.21 to 0.25)
Sex					
Male	2,427,796.12 (1,658,222.99 – 3,398,390.00)	108.98 (74.17–152.26)	4,779,974.99 (3,229,198.87 – 6,666,481.25)	113.35 (76.93–157.86)	0.11 (0.09 to 0.12)
Female	1,410,259.19 (963,822.93 – 1,986,866.03)	60.98 (41.37–85.93)	3,067,469.60 (2,087,476.66 – 4,314,308.09)	69.87 (47.67–97.95)	0.42 (0.41 to 0.43)
SDI regions					
High SDI	445,410.44 (302,453.46 – 629,365.89)	43.46 (29.37–61.72)	734,616.65 (495,089.19 – 1,039,610.69)	45.56 (30.66–64.63)	0.15 (0.12 to 0.18)
High–middle SDI	851,449.18 (577,125.64 – 1,196,402.14)	79.32 (53.63–111.58)	1,654,261.45 (1,112,237.33 – 2,342,966.25)	91.40 (61.49–127.84)	0.49 (0.47 to 0.50)
Middle SDI	1,424,977.92 (972,881.05 – 1,998,624.00)	107.50 (72.62–151.54)	3,014,041.76 (2,027,103.94 – 4,241,933.90)	107.22 (72.41–150.41)	−0.01 (−0.02 to 0)
Low–middle SDI	767792.75 (532,014.92 – 1,074,484.73)	97.38 (66.41–135.99)	1,648,016.96 (1,137,711.94 – 2,286,803.36)	96.87 (66.53–135.34)	−0.11 (−0.15 to −0.08)
Low SDI	345,555.62 (236,904.82 – 480,732.84)	114.32 (78.70–157.77)	791,734.20 (543,965.76 – 1,102,724.52)	111.72 (77.54–154.69)	−0.12 (−0.15 to −0.09)
GBD regions					
East Asia	1,305,543.37 (879,365.14 – 1,831,703.35)	121.06 (81.65–170.84)	2,731,637.08 (1,835,820.06 – 3,881,313.25)	131.71 (88.59–185.33)	0.32 (0.30 to 0.35)
Oceania	2,998.81 (2,014.68 – 4,222.02)	71.07 (48.47–100.09)	7,467.28 (5,009.47 – 10,545.15)	71.69 (48.90–100.85)	−0.03 (−0.05 to −0.01)
Southeast Asia	407,317.37 (279,697.62 – 565,155.62)	120.13 (82.38–167.37)	892,645.36 (607,629.72 – 1,251,756.67)	120.16 (82.07–168.41)	−0.09 (−0.12 to −0.06)
Central Sub–Saharan Africa	33,877.74 (23,148.53 – 47,365.60)	107.72 (74.27–148.25)	85,108.35 (58,390.83 – 118,549.15)	104.55 (72.19–144.19)	−0.09 (−0.10 to −0.08)
Eastern Sub–Saharan Africa	156,845.02 (107,627.65 – 217,066.12)	151.03 (104.06–208.14)	390,368.51 (268,494.30 – 539,441.63)	154.36 (106.98–212.39)	0.06 (−0.02 to 0.15)
Southern Sub–Saharan Africa	21,953.93 (14,849.55 – 30,890.36)	62.77 (42.81–87.27)	43,808.97 (29,799.91 – 61,438.43)	60.88 (41.45–84.90)	−0.03 (−0.06 to −0.01)
Western Sub–Saharan Africa	115,149.41 (79,410.28 – 158,839.71)	100.96 (69.63–140.40)	282,599.16 (192,810.09 – 390,077.32)	97.66 (67.26–136.07)	−0.11 (−0.17 to −0.05)
South Asia	752,640.65 (515,898.64 – 1,056,241.15)	98.76 (67.19–137.20)	1,632,495.53 (1,120,482.94 – 2,264,695.76)	94.63 (64.39–132.24)	−0.27 (−0.32 to −0.22)
Andean Latin America	15,853.48 (10,678.55 – 22,208.87)	61.68 (41.60–86.29)	42,974.35 (28,844.89 – 59,802.30)	67.04 (44.95–93.02)	0.39 (0.34 to 0.43)
Caribbean	16,583.64 (11,120.46 – 23,242.60)	57.48 (38.21–80.94)	31,598.32 (21,033.88 – 44,763.78)	60.15 (40.21–84.96)	0.14 (0.12 to 0.17)
Central Latin America	73,928.04 (50,386.27 – 103,516.76)	67.96 (46.22–95.71)	180,132.05 (121,100.81 – 254,868.46)	68.43 (46.10–96.61)	0.01 (0 to 0.02)
Tropical Latin America	99,601.67 (66,589.07 – 141,045.66)	86.54 (57.67–120.96)	223,661.32 (148,016.30 – 317,898.13)	85.61 (56.91–121.26)	−0.06 (−0.14 to 0.02)
North Africa and Middle East	150,885.03 (104,614.67 – 212,962.52)	69.01 (47.88–97.53)	357,381.91 (244,498.38 – 507,017.54)	63.67 (43.63–90.38)	−0.27 (−0.28 to −0.27)
Central Asia	40,268.70 (26,924.94 – 56,790.13)	74.50 (49.76–105.41)	70,644.02 (47,208.82 – 99,778.41)	74.71 (50.09–104.65)	0 (−0.01 to 0.01)
Central Europe	75,459.48 (50,938.93 – 107,042.77)	52.24 (35.60–73.62)	87215.18 (58,020.19 – 122,997.68)	49.47 (33.20–70.05)	−0.22 (−0.24 to −0.21)
Eastern Europe	155,974.78 (104,289.71 – 219,988.52)	57.65 (38.92–81.55)	167,545.78 (111,322.58 – 236,239.36)	54.92 (36.96–77.8)	−0.15 (−0.16 to −0.14)
Australasia	9,281.96 (6,302.85 – 13,214.33)	41.03 (27.96–58.22)	17,729.86 (11,810.93 – 25,225.50)	41.09 (27.28–58.54)	0.11 (0.03 to 0.19)
High–income Asia Pacific	74,079.16 (49,347.99 – 105,903.92)	36.46 (24.35–51.86)	112,601.14 (76,107.77 – 162,131.71)	35.81 (23.88–51.22)	−0.08 (−0.09 to −0.07)
High–income North America	163,237.32 (111,607.80 – 229,713.43)	51.12 (34.94–71.65)	251,929.11 (170,406.66 – 354,997.71)	46.84 (32.04–65.78)	−0.46 (−0.58 to −0.34)
Southern Latin America	23,888.32 (16,326.16 – 34,163.83)	50.51 (34.55–72.05)	42,907.28 (29,125.17 – 61,509.63)	53.10 (35.90–76.27)	0.18 (0.15 to 0.21)
Western Europe	142,687.42 (96,431.32 – 204,120.52)	29.21 (19.59–41.61)	194,994.03 (131,900.65 – 278,178.89)	29.11 (19.41–41.47)	0.07 (0.04 to 0.10)

GBD, Global Burden of Disease; DALYs, disability-adjusted life years; ASDR, age-standardized DALY rate; CI, confidence interval; SDI, socio-demographic Index; UI, uncertainty interval.

### 3.2 Regional level

The global burden of ONIHL exhibits significant regional variations, closely tied to SDI levels. The highest number of ONIHL DALYs occurred in middle SDI regions both in 1990 (1,424,977.92, 95% UI: 972,881.05–1,998,624.00) and 2021 (3,014,041.76, 95% UI: 2,027,103.94–4,241,933.90) (Table 1 and Figure 1A). The ASDR demonstrated notable disparities, with low SDI regions experiencing the highest rate of 111.72 per 100,000 individuals (95% UI: 77.54–154.69), whereas high SDI regions reported the lowest rate of 45.56 per 100,000 (95% UI: 30.66–64.63) (Table 1 and Figure 1A). The ASDR remained stable or slightly decreased in the middle, low-middle, and low SDI regions. In contrast, the ASDR showed remarkable growth in high-middle SDI regions, where the ASDR increased from 79.32 (95% UI: 53.63–111.58) in 1990 to 91.40 (95% UI: 61.49–127.84) per 100,000 in 2021, with an EAPC of 0.49 (95% CI: 0.47 to 0.50). In particular, for females in high-middle SDI regions, the ASDR increased from 61.61 (95% UI: 41.30–86.57) in 1990 to 74.33 (95% UI: 49.73–104.68) per 100,000 in 2021, representing an increase of 20.65% (Table 1 and Figure 1A). Moreover, the occupational noise-related SEVs exhibited the same distribution characteristics, with the highest value occurring in low SDI regions and the lowest value occurring in high SDI regions. The male occupational noise-related SEV showed a decreasing trend across all the SDI regions, whereas the trend differed for females: except for the middle SDI regions (where the trend was decreasing), the remaining SDI regions exhibited an increasing trend, particularly in the low-middle- and high-middle SDI regions (Supplementary Table S1 and Figure 1B).

**Figure 2:**
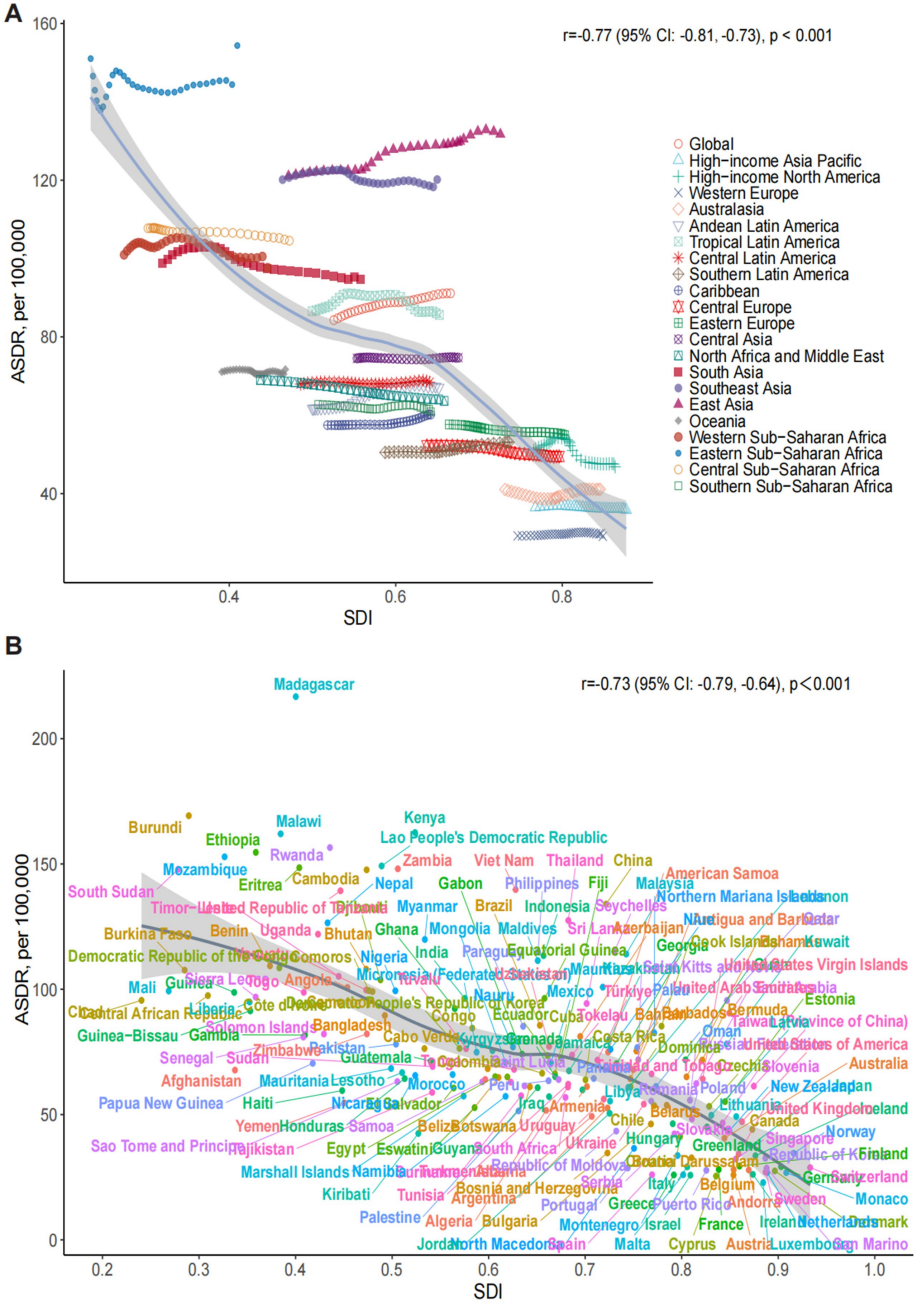
Association between the SDI and the ASDR of ONIHL. **(A)** Trends in 21 regions, 1990–2021. **(B)** Estimates for 204 countries and territories, 2021. The blue line represents the expected ASDR of ONIHL based solely on the SDI. ASDR, age-standardized DALY rate; SDI, socio-demographic index; ONIHL, occupational noise-induced hearing loss.

Spearman correlation analysis revealed a significant negative correlation between the ASDR of ONIHL and the SDI across 21 global regions (*r* = −0.77, 95% CI: −0.81 to −0.73, *p* < 0.001), indicating that regions with lower SDI (e.g., Sub-Saharan Africa) have greater disease burdens of ONIHL (Figure 2A). In terms of GBD regions, East Asia had the highest figure, reaching 2,731,637.08 (95% UI: 1,835,820.06–3,881,313.25), followed by South Asia, Southeast Asia, Eastern Sub-Saharan Africa, and North Africa and Middle East. These regions experience the greatest burden in the world. In 2021, the highest ASDR occurred in Eastern Sub-Saharan Africa at 154.36 per 100,000 individuals (95% UI: 106.98–212.39), followed by East Asia, Southeast Asia, Western Sub-Saharan Africa, and South Asia. From 1990 to 2021, the ASDR of ONIHL increased the most in Andean Latin America (EAPC 0.39, 95% CI: 0.34 to 0.43), followed closely by East Asia (EAPC 0.32, 95% CI: 0.30 to 0.35), and decreased the most in high-income North America (EAPC: –0.46, 95% CI: −0.58 to −0.34) (Table 1 and Figure 2A).

In terms of SEV related to ONIHL, Eastern Sub-Saharan Africa and East Asia ranked the highest, at 14.80% (95% UI: 14.30–15.53%) and 14.55% (95% UI: 14.01–15.24%), respectively, whereas Western Europe had the lowest SEV (6.26, 95% UI: 5.97–6.71%). Most regions showed a decreasing trend, whereas Andean Latin America and Southern Latin America exhibited notable increases. The trends of SEV related to ONIHL differed markedly between males and females. For males, all regions declined except Latin America and the Caribbean. For females, however, all regions except Eastern Europe, Central Europe, and Eastern Sub-Saharan Africa experienced varying increases—notably in Latin America and the Caribbean (Supplementary Table S1).

### 3.3 National level

**Figure 3:**
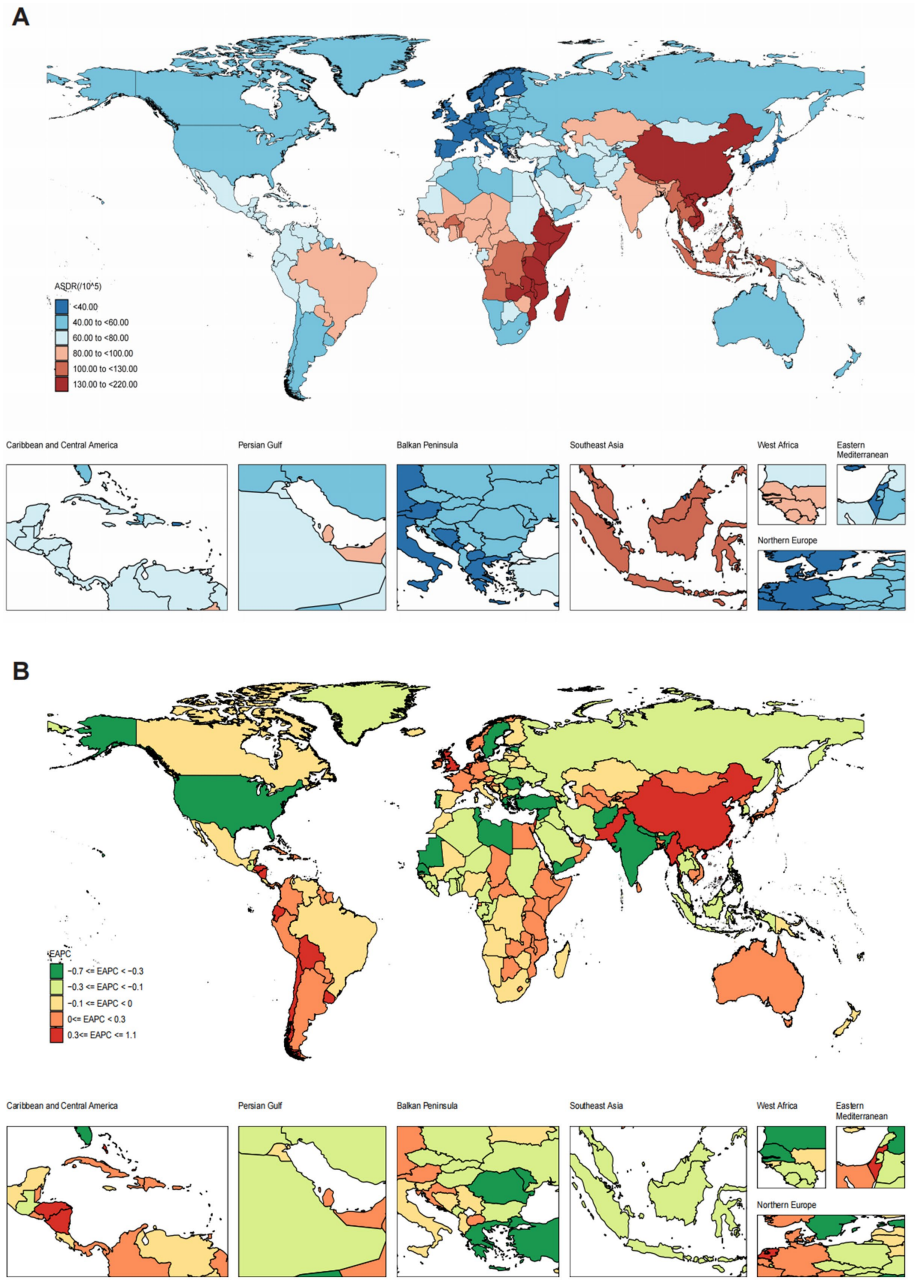
Spatiotemporal distribution map of ONIHL among 204 countries and territories. **(A)** ASDR in 2021. **(B)** EAPC in the ASDR from 1990 to 2021. ASDR, age-standardized DALY rate; EAPC, estimated annual percentage change; ONIHL, occupational noise-induced hearing loss.

Across 204 countries and territories, the SDI was significantly negatively correlated with the ONIHL burden (*r* = −0.73, 95% CI: −0.79 to −0.64, *p* < 0.001) (Figure 2B). In 2021, China had the highest number of ONIHL DALYs at 2,683,891.80 (95% UI: 1,804,391.74–3,813,460.13), followed by India, Indonesia, the United States of America, and Brazil (Supplementary Table S2). The ASDR of ONIHL varies from approximately 22.87 to 216.77 per 100,000 individuals. Among all the countries, Madagascar (216.77 per 100,000, 95% UI: 148.91–297.71), Burundi (169.25 per 100,000, 95% UI: 118.18–235.28), Kenya (162.52 per 100,000, 95% UI: 110.79–223.47), Malawi (162.01 per 100,000, 95% UI: 113.41–224.12), and Rwanda (156.52 per 100,000, 95% UI: 107.15–216.45) presented the highest ASDRs (Figure 3A and Supplementary Table S2). Notably, these five countries are located in Sub-Saharan Africa. Conversely, Luxembourg (22.87 per 100,000, 95% UI: 15.09–33.20) presented the lowest ASDR, followed by Cyprus, Israel, Greece, and Belgium. The most significant increases in ASDR were observed in the Solomon Islands (EAPC 1.08, 95% CI: 0.89 to 1.27), followed by Honduras, Bolivia, Kiribati, and the Netherlands; São Tomé and Príncipe (EAPC –0.66, 95% CI: −0.74 to −0.59) showed the steepest decline, followed by the Syrian Arab Republic, Libya, Turkey, and the United States of America (Figure 3B and Supplementary Table S2). Notably, as the most populous countries, China and India had ASDRs of 133.99 per 100,000 (95% UI: 90.16–188.50) and 96.37 per 100,000 (95% UI: 65.17–134.57) in 2021, respectively, with the EAPCs in the ASDRs from 1990 to 2021 being 0.32 (95% CI: 0.30 to 0.35) and −0.36 (95% CI: −0.43 to −0.30), respectively (Figure 3 and Supplementary Table S2).

Our analysis of the SEV related to occupational noise in 204 countries revealed that occupational noise exposure also varied considerably from country to country (Supplementary Table S3). Madagascar ranked first in SEV (19.10, 95% UI: 18.48–19.86), followed by Burundi, Tanzania, Cambodia, Lao People’s Democratic Republic, Nepal, Malawi, Vietnam, Rwanda, and Uganda. Notably, in general, the SEV level of countries with lower SDIs was much higher than that of countries with higher SDIs. Additionally, among the top 20 countries, China, as a high-middle SDI country, had an SEV of 14.69 in 2021 (95% UI: 14.15–15.38), whereas Qatar, a high-SDI country, had an SEV of 14.24 (95% UI: 13.69–14.90). Montenegro had the lowest SEV related to occupational noise, at 3.60 (95% UI: 3.40–3.93).

### 3.4 Age and sex patterns

**Figure 4:**
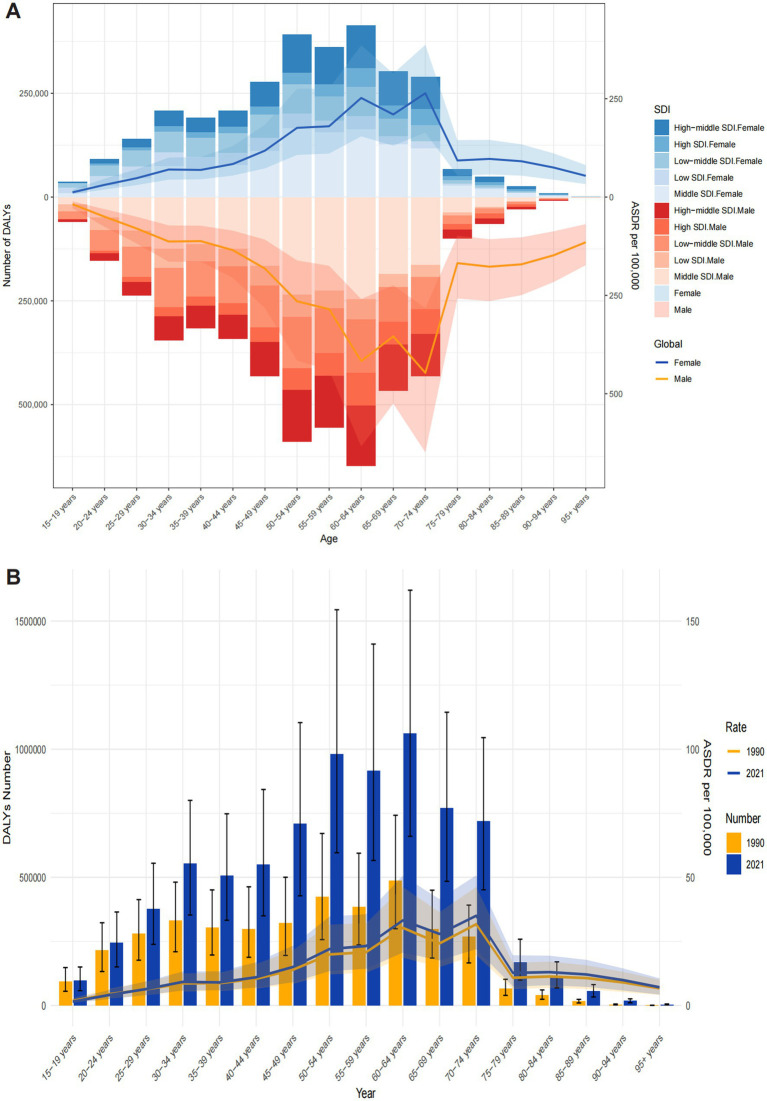
Age-specific DALYs and ASDRs of ONIHL. **(A)** By sex and SDI in 2021. **(B)** By year between 1990 and 2021. ASDR, age-standardized DALY rate; DALYs, disability-adjusted life years; ONIHL, occupational noise-induced hearing loss; SDI, Socio-demographic Index.

In terms of age, ONIHL data are only available for individuals aged 15 years and above from 1990 to 2021 (Figure 4). Both the number of ONIHL DALYs and the ASDR increased progressively with age: the DALYs peaked in the 60–64 age group, and the ASDR peaked in the 60–64 and 70–74 age groups. These data indicate that the ONIHL burden primarily occurs in individuals aged 45–74 years. Both the number of ONIHL DALYs and the ASDR were significantly greater in males than in females in all age groups. Notably, among all the SDI regions, the middle SDI regions had the highest proportions of ONIHL DALYs, and the proportion of DALYs in the low SDI regions decreased progressively with increasing age (Figure 4A). The numbers of ONIHL DALYs and ASDRs in 1990 and 2021 exhibited similar age patterns. Compared with that in 1990, the number of ONIHL DALYs in 2021 clearly increased in all age groups, whereas the ASDR of ONIHL slightly increased (Figure 4B).

### 3.5 Cross-country inequality analysis

**Figure 5:**
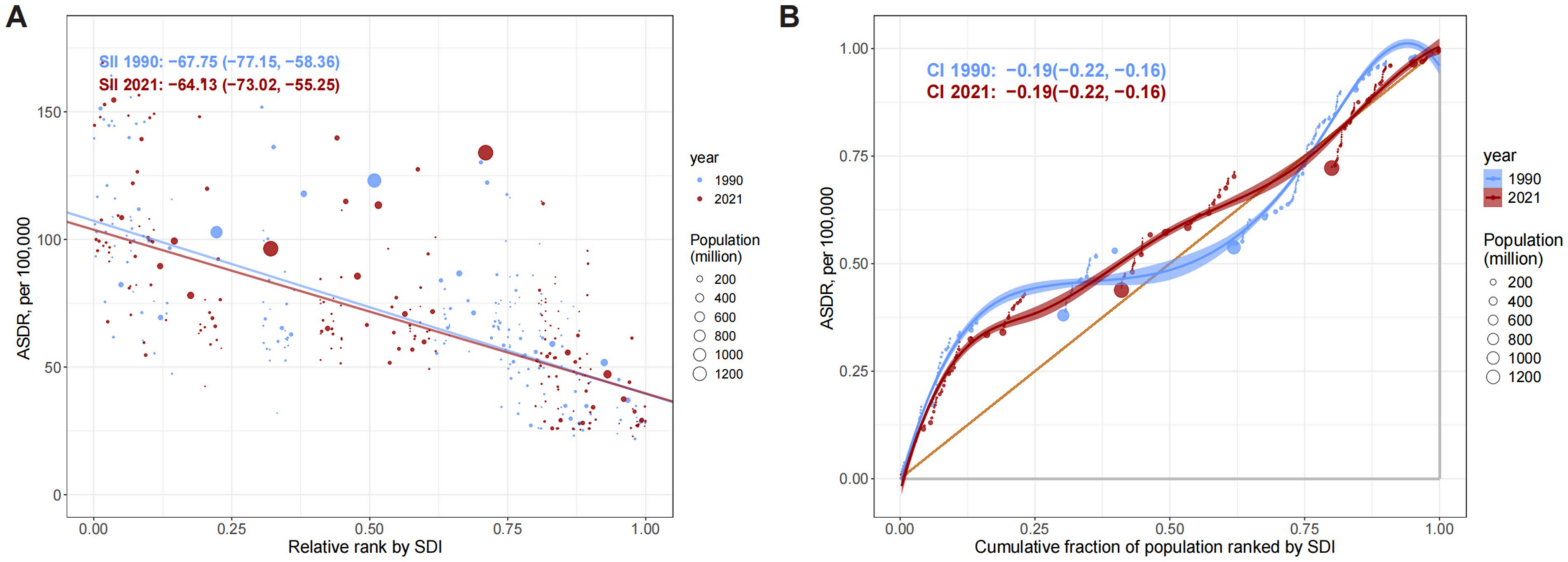
Absolute and relative cross-country inequality in the ASDR of ONIHL, 1990–2021. **(A)** Health inequality regression curves. **(B)** Concentration curves. ASDR, age-standardized DALY rate; ONIHL, occupational noise-induced hearing loss; SDI, Socio-demographic Index.

For the ONIHL burden, we observed significant absolute and relative inequalities associated with the SDI. The SII and CI were negative values were negative values, indicating that lower SDI countries/territories disproportionately bear a greater burden (Figure 5). The SII revealed that, between the highest and lowest SDI countries/territories, the ASDR gap declined from −66.75 (95% CI: −77.15 to −58.36) in 1990 to −64.13 (95% CI: −73.02 to −55.25) in 2021 (Figure 5A). The CI for the ASDR was −0.19 (95% CI: −0.22 to −0.16) in both 1990 and 2021 (Figure 5B). The absolute values of both indices did not decrease significantly, indicating that the absolute and relative cross-country health inequalities in the ONIHL burden did not improve.

### 3.6 Decomposition analysis

**Figure 6:**
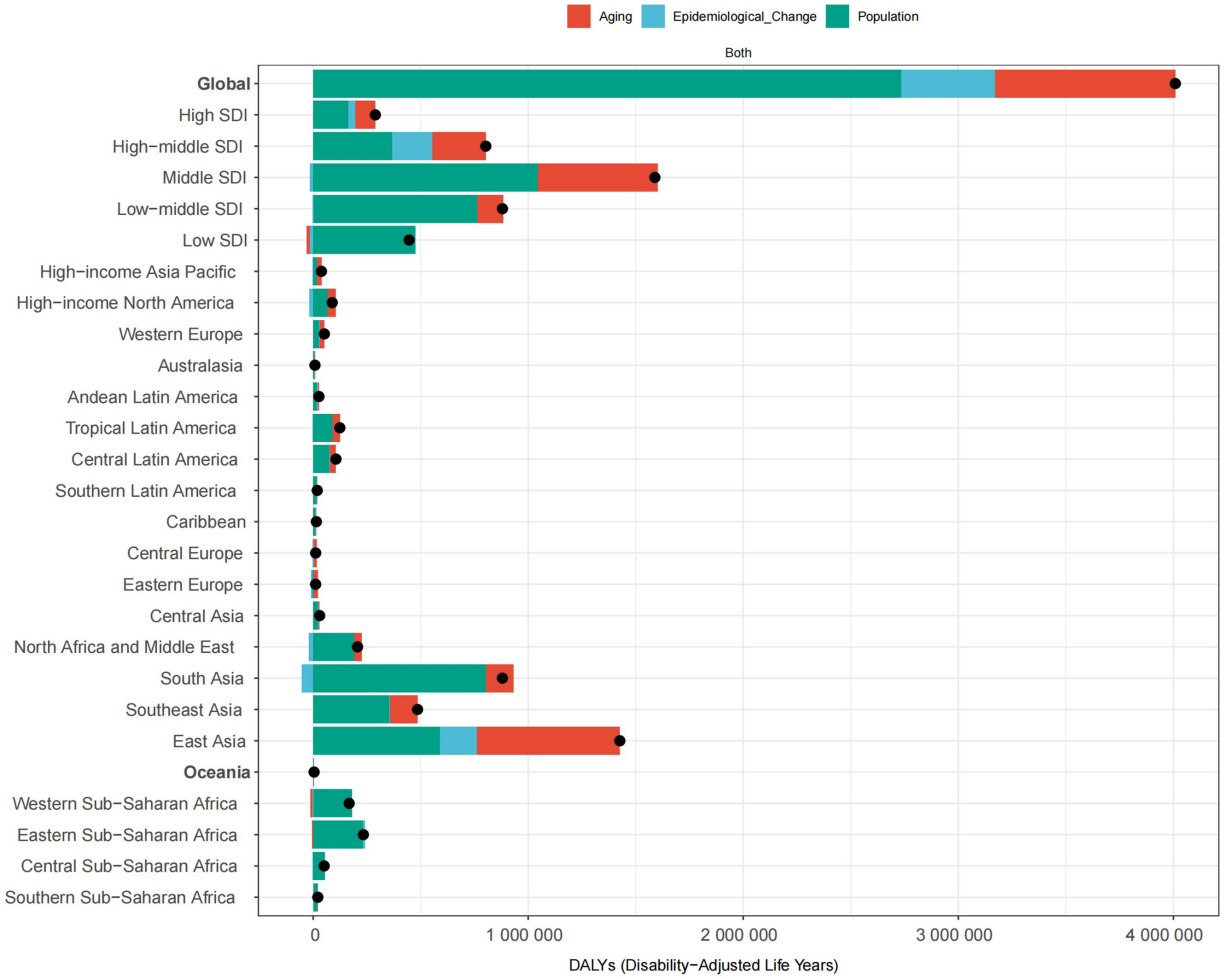
Changes in ONIHL DALYs globally, in various SDI regions, and in 21 GBD regions, 1990–2021 (driven by population growth, aging, and epidemiology). The black dots represent the total change contributed by all three components. A positive value for each component indicates a positive contribution to ONIHL DALYs, and a negative value indicates a negative contribution. ASDR, age-standardized DALY rate. ONIHL, occupational noise-induced hearing loss; SDI, Socio-demographic Index; GBD, Global Burden of Disease study.

By performing decomposition analysis on the number of DALYs of ONIHL, this study evaluated the impacts of aging, population growth, and epidemiological changes on ONIHL from 1990 to 2021 (Figure 6 and Supplementary Table S4). Overall, the DALYs associated with ONIHL showed an increasing trend globally and in all the SDI regions. Population growth, aging, and epidemiological changes contributed 68.23%, 20.94%, and 10.83%, respectively, to the increase in disease burden globally. Population growth was the dominant factor contributing to the increase in burden. Notably, low SDI regions exhibited excessive population compensation (106.63%), suggesting epidemiological deterioration. In East Asia, the contribution of aging to the disease burden (46.75%) exceeded that of population growth (41.31%), indicating that the disease burden is “aging-dominated.” In some regions of South Asia, Eastern Sub-Saharan Africa, and North Africa and the Middle East, the contribution of population growth to the disease burden exceeded 90%, indicating “population growth dominated.”

### 3.7 Frontier analysis

**Figure 7:**
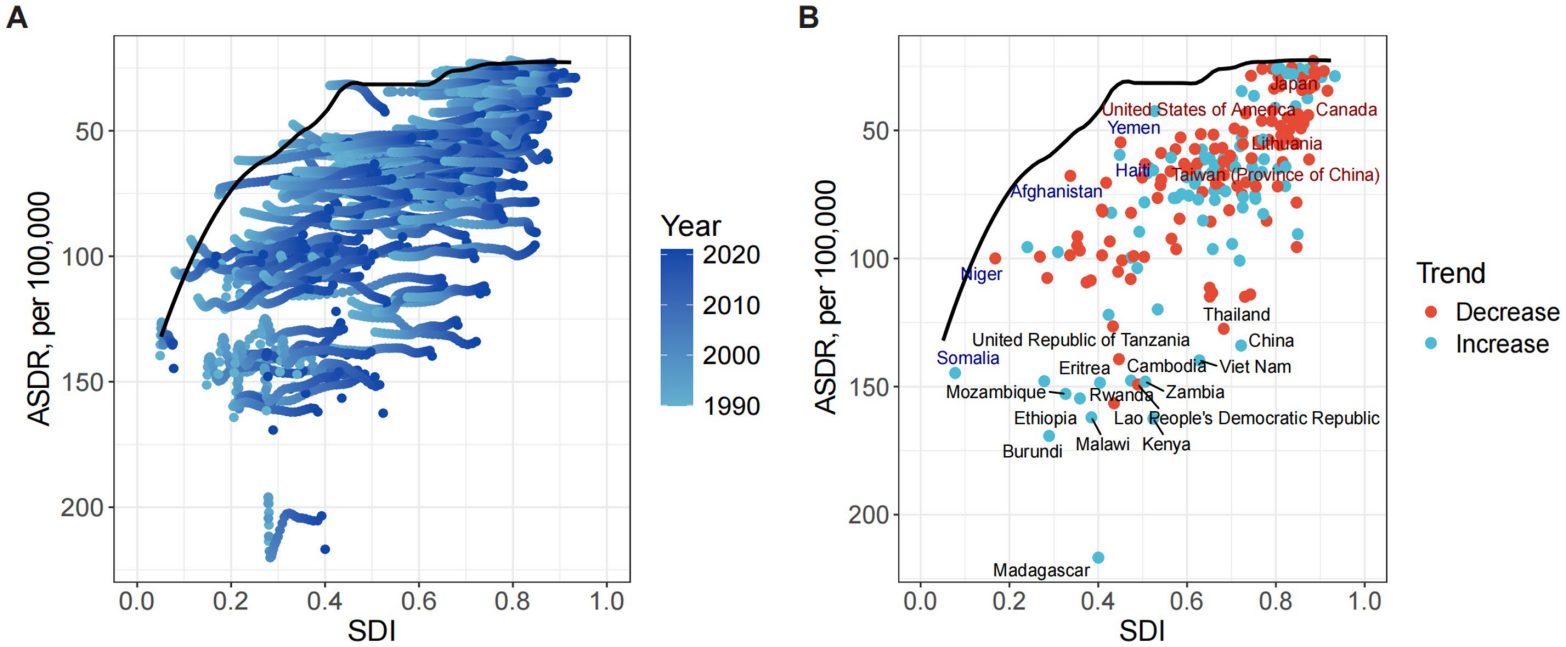
Frontier analysis exploring the relationship between the SDI and ASDR of ONIHL in 204 countries and territories. **(A)** Light blue (1990) to dark blue (2021) indicate the change over time. The frontier line delineates the countries and territories with the lowest ASDRs (optimal performers) given their SDIs. **(B)** Each point represents a specific country or territory in 2021, the frontier line is shown in black, and the top 15 countries and territories with the largest differences from the frontier are marked in black. The direction of the change in the ASDR from 1990 to 2021 is indicated by the color of the dots, with red dots representing decreases and blue dots representing increases. ASDR, age-standardized DALY rate; ONIHL, occupational noise-induced hearing loss; SDI, Socio-demographic Index.

To explore possible reductions in the ASDR, frontier analysis was performed using the SDI as a factor. The efficiency difference for a given SDI generally decreased with increasing global SDI (Figure 7). The 15 countries with the largest actual differences (efficiency difference range: 97.65–168.77 per 100,000 people) included Madagascar, Kenya, Rwanda, Lao People’s Democratic Republic, Zambia, Cambodia, Malawi, China, Vietnam, the United Republic of Tanzania, Burundi, Ethiopia, Thailand, Eritrea, and Mozambique. Low-income countries such as Madagascar (efficiency difference = 168.77), Kenya (131.00), and Rwanda (124.81) presented the largest efficiency gaps. The actual ASDRs of these countries were significantly higher than the theoretical optimal values (frontier) at the same SDI level, indicating that there is an enormous avoidable disease burden in these countries. Although China (SDI ~ 0.72) is an upper-middle-income country, the efficiency difference reached 109.66, suggesting that there may be structural deficiencies in its ONIHL prevention and control system. Countries with extremely low SDIs (SDI < 0.20), such as Niger (5.56) and Somalia (18.03), had actual performances close to the optimal level under their socio-economic conditions. The ASDRs of high-income countries such as Japan (14.78), Canada (21.45), and the United States (24.59) were highly consistent with the theoretical frontier, reflecting the high efficiency of their health resource allocation (Figure 7 and Supplementary Table S5).

### 3.8 Future forecasts of the global burden of ONIHL

**Figure 8:**
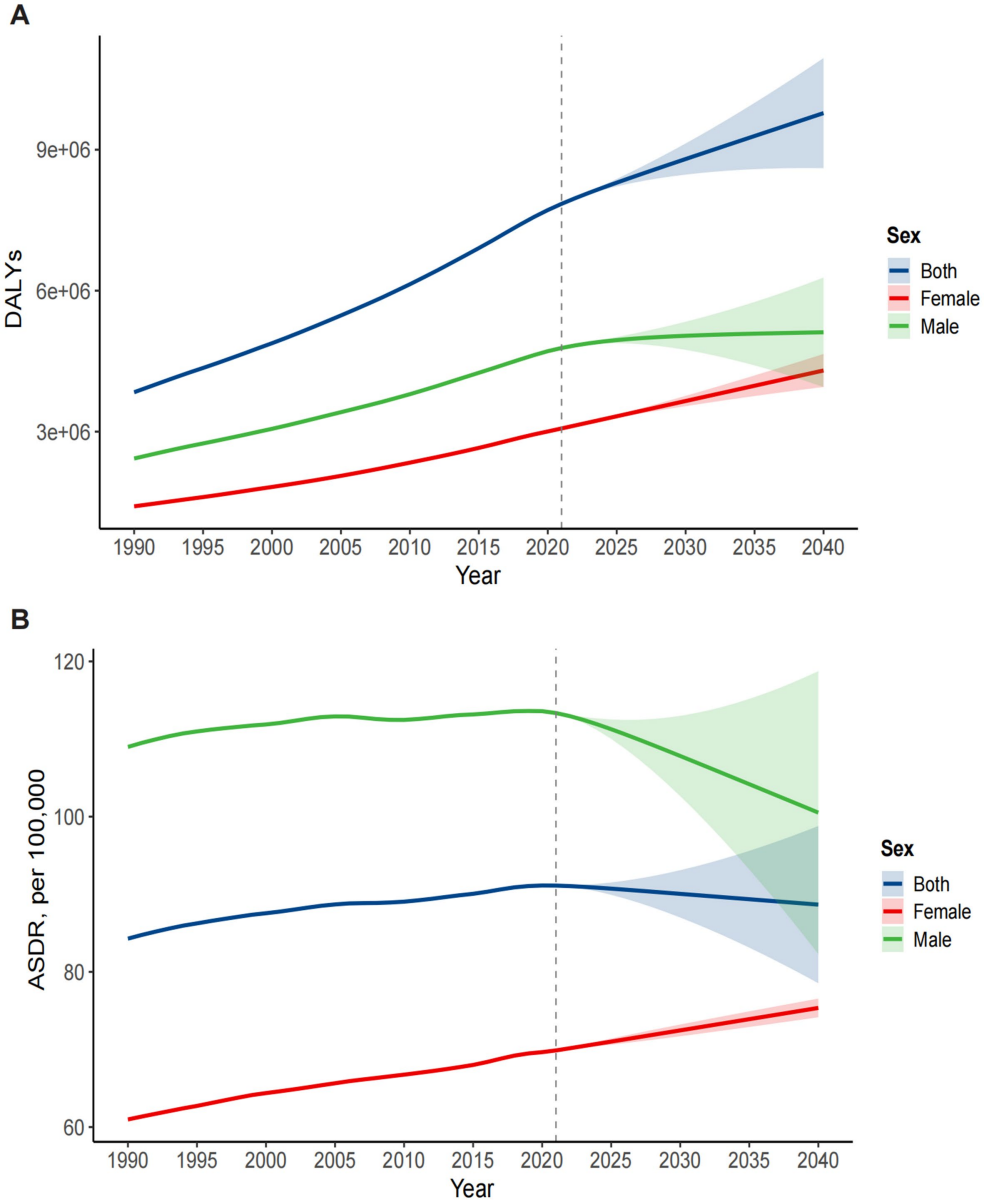
Prediction of the disease burden trend of ONIHL for both sexes from 1990 to 2040 via the ARIMA model. **(A)** DALYs. **(B)** ASDR. ASDR: age-standardized DALY rate. DALYs, disability-adjusted life years; ONIHL, occupational noise-induced hearing loss; SDI, Socio-demographic Index; ARIMA, autoregressive integrated moving average.

The global burden of ONIHL is projected to evolve significantly from 2022 to 2040 according to the ARIMA model (Figure 8 and Supplementary Table S6). The global and male ONIHL DALYs are expected to increase from 7,969,135.74 (95% UI: 7,960,571.70–7,977,699.79) and 4,835,323.70 (95% UI: 4,828,859.51–4,841,787.90) in 2022 to 9,778,997.44 (95% UI: 8,607,164.25–10,950,830.62) and 5,113,855.40 (95% UI: 3,948,769.84–6,278,940.97) by 2040, respectively. The increase in DALYs among males is slowing. In contrast, there is a significant increase in DALYs among females, with an increase of approximately 1.17 million DALYs, increasing from 3,131,992.22 (95% UI: 3,128,130.23–3,135,854.22) in 2022 to 4,301,293.44 (95% UI: 3,950,073.01–4,652,513.88) by 2040 (Figure 8A and Supplementary Table S6). The global ASDR of ONIHL shows different trends between the sexes: it is decreasing overall and among males but increasing significantly among females. Specifically, the global and male ASDRs are expected to decrease from 91.05 (95% UI: 90.96–91.14) and 112.96 (95% UI: 112.82–113.09) per 100,000 individuals in 2022 to 88.66 (95% UI: 78.53–98.79) and 100.52 (95% UI: 82.29–118.75) per 100,000 by 2040, respectively. However, the global ASDR for females is expected to increase from 70.16 (95% UI: 70.08–70.23) per 100,000 in 2022 to 75.35 (95% UI: 74.15–76.56) per 100,000 by 2040 (Figure 8B and Supplementary Table S6).

## 4 Discussion

### 4.1 Global trends and regional disparities

Using the latest data from GBD 2021, we described the epidemiological characteristics and spatiotemporal patterns of the ONIHL burden globally and regionally and identified the general growth trends of the disease burden and SEV related to occupational noise from 1990 to 2021. The trend of the ASDR of ONIHL, both globally and across the SDI and GBD regions, generally corresponds to the changes in SEV levels. By applying regional, Spearman correlation, inequality, decomposition, and frontier analyses, were identified marked disparities across geographic and socioeconomic regions, revealing that lower SDI regions presented greater ONIHL burdens and that high-middle SDI regions and females faced greater burdens in 2021 than in 1990. Understanding the reasons behind these trends and disparities is crucial for shaping targeted prevention strategies and policy interventions.

On a global scale, the ASDR of ONIHL increased from 1990 to 2021, which was different from previous GBD studies over time, primarily because of the updates and iterations of data and algorithms (6, 24, 25). Notably, regardless of the slight decline or increase in the ASDR, the absolute number of DALYs has been increasing in almost all countries—with the global figure doubling from 1990 to 2021—which inevitably increases the medical and economic burden on society. Predictive analysis shows that the global ASDR will decrease, while DALYs will continue to increase, which may be mainly due to population growth and changes in population structure caused by aging. The higher ASDR was in lower SDI regions, with Spearman analysis revealing a significant negative correlation between ONIHL’s ASDR and SDI across 21 GBD regions. These findings highlight the persistent socioeconomic disparities in the global burden of ONIHL. The greater burden in low SDI regions is likely the result of a combination of factors: inadequate occupational safety regulations, limited access to hearing protection, underinvestment in workplace health surveillance, inadequate management of occupational risk factors, and restricted access to healthcare services (24, 42, 43). By comparison, high SDI regions have probably gained advantages from mature healthcare systems, efficacious public health initiatives, stricter noise exposure limits, and greater awareness of the factors and symptoms associated with ONIHL (44).

Regionally, East Asia, South Asia, Southeast Asia, and Eastern Sub-Saharan Africa bear the heaviest ONIHL burden. These regions have witnessed rapid industrialization over the past 30 years, and alongside this, many workers have engaged in industrial development, naturally resulting in a growing population exposed to occupational noise (45). Unfortunately, this rise in exposure coincided with insufficient awareness of occupational noise hazards, substandard working conditions, and inadequate hearing protection—factors that together contributed to a high incidence of ONIHL during this period (46). Moreover, limited access to healthcare services—exacerbated by shortages of medical personnel—further compounds the burden (20). Higher SDI regions have enforced rigorous legal restrictions on occupational noise and imposed strict limits on workplace noise levels, with High-Income North America, for example, seeing the largest decline (EAPC: −0.46) owing to these measures (47).

### 4.2 Sex differences and age effects

Our study revealed that males consistently had higher ONIHL burden than females, which aligns with the findings of previous studies (6, 24–26). This gap might stem from the interaction between biological susceptibility and social behavioral factors. Biologically, males exhibit greater susceptibility to noise-induced auditory damage, partly due to reduced cochlear antioxidant capacity and vascular density, which increase vulnerability to oxidative stress (48). Moreover, females generally have better hearing than males of the same age, which can be attributed to the protective role of estrogen and its receptors in preserving hearing function (49). High-intensity noise exposure is common in occupational environments, such as those in manufacturing, transportation, mining, construction, and agriculture, which are the occupations at highest risk for NIHL, and men are more likely to work in environments with poor working conditions and greater, prolonged noise exposure (50). Additionally, smoking is more prevalent among men: it exposes smokers to various toxic substances, which damage cochlear hair cells by increasing carboxyhemoglobin levels or reducing cochlear blood flow (51). When smoking is combined with occupational noise, these substances can substantially increase susceptibility to ONIHL (52).

However, a worrying issue is that the occupational noise risk and burden of ONIHL among females are continuously increasing. Projections to 2040 highlight a paradox: while global and male ASDRs are expected to decline, female ASDRs will rise, and female DALYs are expected to increase by approximately 1.17 million. This underscores the urgency of proactive measures, particularly for female workers in growing industries. This trend may be attributed to several factors. First, female participation in traditionally male-dominated industries has further increased (53). With adjustments in the labor market structure, the proportion of females employed in traditional high-noise industries such as manufacturing and construction has gradually increased (54). Second, the global prevalence of smoking among females has increased in recent years. One main contributing factor is the increased promotion of tobacco use among women by various tobacco companies in both high- and low-income countries. Additionally, exposure to both noise and smoking may have a combined effect on HL in females, potentially exacerbating the burden of ONIHL (55, 56). Third, regarding sex differences in the use of hearing protection devices (HPDs), researchers have reported that female workers have a significantly greater prevalence of nonuse of HPDs. This may be attributed to females experiencing greater sensitivity to discomfort, pressure, and even headaches associated with wearing HPDs (57). Fourth, after women have reached menopause, the weakening of the protective effect of estrogen against noise may also be a contributing factor.

Our results also indicate that with increasing age, both the DALYs and ASDRs of ONIHL generally exhibit an increasing trend and are mainly concentrated in middle-aged and older populations. This phenomenon stems from the progressive characteristic of ONIHL, with the degree of noise-related damage to the auditory system being closely linked to noise exposure duration, noise properties, and intensity. Middle-aged and older workers typically have more than a decade or more of work experience, resulting in the buildup of noise exposure in both duration and intensity, emphasizing the importance of early intervention and long-term monitoring in the workforce (58). Additionally, aging itself and noise exposure may have overlapping effects, coupled with other risk factors for HL in older adults, such as hypertension, diabetes, and drug-induced deafness (59).

### 4.3 Special analysis and policy implications

Cross-country inequality analysis revealed that despite advancements in global public health measures over the past 30 years, the absolute and relative cross-country health inequalities in ONIHL have not improved significantly, highlighting the need to strengthen targeted interventions for vulnerable groups. Several factors contribute to this persistence. A key driver lies in economic and industrial disparities: low- and middle-income countries (LMICs), heavily reliant on noise-intensive sectors (e.g., manufacturing, mining) for growth, face barriers to investing in noise-control technologies due to limited resources, while high-income countries have shifted such industries offshore. Weak policy implementation further perpetuates gaps—most LMICs lack the institutional capacity to enforce noise standards, with low penalties for violations reducing employer accountability. Additionally, disparities in healthcare access (e.g., limited hearing screenings in LMICs) and worker awareness (e.g., inadequate education on risks) widen the divide (60). Countries should integrate hearing health related to occupational noise exposure into the priorities of Universal Health Coverage (UHC) and reduce health disparities through social security policies, especially in LMICs (44). Decomposition analysis revealed that population growth was the primary contributor to increasing DALYs globally. The East Asian region is “aging-dominated”; thus, priority should be given to establishing a hearing healthcare system for older individuals. In less developed regions such as South Asia and Eastern Sub-Saharan Africa, efforts should focus on addressing population growth-driven challenges by strengthening healthcare infrastructure and expanding early detection programs to better mitigate associated burdens.

Frontier analysis reveals significant inefficiencies in ONIHL management: countries such as Madagascar and Burundi present large gaps between their actual ASDRs and theoretical minima, indicating a substantial avoidable burden. Even upper-middle-income countries such as China show notable inefficiencies, suggesting structural weaknesses in their prevention systems such as uneven regulatory enforcement—where coastal industrial hubs maintain stricter noise control oversight whereas inland regions lack sufficient occupational health inspectors—as well as potentially inadequate access to hearing care (26). In contrast, high SDI countries (e.g., the U. S., Canada) align closely with theoretical frontiers, demonstrating the effectiveness of integrated policies that combine regulation, surveillance, and worker education. Low SDI countries and regions such as Yemen and Haiti also perform well, which indicates that SDI is not the sole determinant of health outcomes; governance capacity and health system efficiency are equally critical. The frontier analysis has limitations, though: its sensitivity to national SDI aggregation and potential data gaps in low SDI countries could marginally influence result interpretation. In addition to the current significant burden, ARIMA model projections indicate that the global burden of ONIHL will continue to pose ongoing challenges, particularly for females. It is important to note, however, that there is a limitation in the completeness of historical GBD data, particularly in gender-disaggregated records, which may constrain the reliability of these projections. Additionally, the ARIMA model relies on the assumption of the continuity of historical trends, while unforeseen factors such as the implementation of large-scale noise control policies and advancements in hearing protection technology may undermine this assumption, leading to forecasting biases.

The majority of ONIHL cases are preventable (2), and measures need to be taken to address the ONIHL burden. From a macro perspective, first, in higher-SDI regions, the ONIHL DALYs and ASDRs have been effectively controlled because the promulgation of regulations has reduced occupational noise, as has the implementation of governance measures. Thus, less developed regions should draw on relevant policies and experience from developed regions. Second, strengthening international cooperation in sharing best practices between countries is essential for reducing the ONIHL burden, and for less developed regions, guidance and support can be provided on occupational noise protection policies and their implementation. Third, targeted protection measures should be prioritized for female workers in high-noise roles, with interventions tailored to their specific workplace risks. From a micro perspective, first, workplace noise control and personal protection should be increased, and industrial activities should be conducted in strict accordance with the set thresholds for occupational noise. Second, efforts should be made to strengthen onsite hearing monitoring, implement early, regular, and long-term screenings, and provide prompt intervention for workers with early signs of HL. Additionally, establishing personal hearing screening records and integrating ONIHL into the chronic disease management system is also a recommended policy measure. Third, workers should be provided with regular occupational health training to increase their self-protection awareness and capabilities and improve their compliance with HPD use (61). For instance, use contextualized training (local language videos, hands-on HPD fitting) to address knowledge gaps in occupational noise risks and HPD use. Fourth, noise monitoring points should be established, with real-time noise level monitoring, and protective equipment use adjusted based on noise intensity. Additionally, targeting the projected increase in female ONIHL burden (particularly in high-middle SDI regions), employers should develop gender-responsive HPDs (e.g., smaller ear canal sizes) and prioritize female-dominated sectors (e.g., garment manufacturing) for enhanced monitoring.

### 4.4 Limitations

This study also has several limitations that should be considered. First, the precision of estimates may be affected by data quality and availability across countries/regions. In some LMICs, limited reliable epidemiological data and underreported ONIHL cases may underestimate the true burden. Second, confounding factors, including occupational exposure to toxic substances and individual comorbidities such as hypertension or diabetes, may have influenced data interpretation. Third, racial factors—known to significantly impact disease burden—were not included in the GBD datasets, and genetic susceptibility may also influence the development of ONIHL.

## 5 Conclusion

In summary, our research revealed that the global burden of ONIHL has grown from 1990 to 2021, highlighting the threat of ONIHL to global, regional, and national public health—underscoring the urgency for policy action—by integrating cross-country inequality, decomposition, frontier, and ARIMA forecasting analyses to generate targeted evidence. Key findings include: lower-SDI countries bear a disproportionate ONIHL burden, with no improvement in global health inequalities, thereby offering a quantifiable basis for equity-focused policies. Additionally, population growth is identified as the primary global driver of ONIHL burden. Furthermore, quantifying “efficiency gaps” between nations’ actual burden and optimal levels provides an actionable tool for identifying targeted improvement opportunities. Finally, ARIMA forecasting projects ONIHL trends through 2040, with a critical warning that the increase in female burden will far outpace that of men. These findings and trends underscore the need for targeted interventions: strengthening occupational safety regulations specifically in lower SDI regions, implementing gender-responsive policies to protect female workers in high-noise industries, and prioritizing early detection and prevention in middle-aged and older populations. Addressing these disparities will require coordinated, targeted efforts aligned with our study’s findings: prioritizing the closure of frontier gaps by scaling up cost-effective noise monitoring equipment and industry-adapted HPDs in low-SDI countries lagging behind the optimal frontier; enhancing surveillance of HPD compliance in female-dominated high-noise sectors while delivering gender-tailored training to address usage barriers; and adapting hearing care access strategies to regional contexts—such as providing portable hearing screeners for resource-limited regions and integrating ONIHL check-ups into older care programs in aging populations. These actions will ultimately mitigate the avoidable global burden of ONIHL.

## Data Availability

The original contributions presented in the study are included in the article/Supplementary material, further inquiries can be directed to the corresponding authors.
