# Supplementary material for: Global burden and trends of occupational noise-induced hearing loss (1990–2021) and projection to 2040
Source: Front Public Health. 2025 Sep 22;13:1682413. doi: 10.3389/fpubh.2025.1682413 (PMC12497797; doi:10.3389/fpubh.2025.1682413)
Supplement: Supplementary file 1 [file Data_Sheet_1.PDF]

Supplementary Materials

Table S1. Global and regional summary exposure values (SEV) for occupational noise-induced hearing loss (1990 and 2021).

Location	Both		Male		Female	
	**SEV in 1990, %, (95% UI)**	**SEV in 2021, %, (95% UI)**	**SEV in 1990, %, (95% UI)**	**SEV in 2021, % , (95% UI)**	**SEV in 1990, %, (95% UI)**	**SEV in 2021, %, (95% UI)**
**Global**	10.63 (10.21–11.19)	10.77 (10.36–11.34)	13.64 (13.1–14.34)	13.40 (12.89–14.11)	7.76 (7.45–8.22)	8.25 (7.93–8.73)
**SDI regions**						
High SDI	6.98 (6.65–7.46)	7.08 (6.74–7.58)	8.78 (8.37–9.42)	8.44 (8.05–9.07)	5.35 (5.1–5.75)	5.74 (5.47–6.14)
High–middle SDI	10.48 (10.09–11.06)	10.80 a(10.38–11.35)	12.81 (12.25–13.52)	12.50 (12–13.22)	8.41 (8.05–8.91)	9.21 (8.85–9.72)
Middle SDI	12.96 (12.47–13.63)	12.05 (11.57–12.7)	15.90 (15.3–16.68)	14.73 (14.15–15.51)	10.03 (9.63–10.63)	9.46 (9.06–10.01)
Low–middle SDI	11.07 (10.55–11.76)	11.20 (10.78–11.8)	15.93 (15.17–16.8)	15.50 (14.92–16.27)	6.07 (5.52–6.68)	7.08 (6.80–7.52)
Low SDI	12.87 (12.36–13.53)	12.53 (12.07–13.15)	16.45 (15.81–17.26)	15.77 (15.21–16.51)	9.26 (8.81–9.82)	9.4 (9.05–9.93)
**GBD regions**						
East Asia	15.07 (14.53–15.75)	14.55 (14.01–15.24)	16.70 (16.04–17.55)	15.39 (14.8–16.16)	13.46 (12.9–14.16)	13.77 (13.25–14.4)
Oceania	8.49 (7.81–9.28)	8.59 (8.16–9.17)	10.99 (10.05–12.02)	9.93 (9.41–10.69)	5.84 (5.13–6.60)	7.17 (6.71–7.67)
Southeast Asia	12.48 (11.93–13.22)	12.55 (12.06–13.25)	15.64 (14.94–16.39)	15.40 (14.82–16.18)	9.57 (9.02–10.33)	9.87 (9.46–10.55)
Central Sub–Saharan Africa	12.66 (11.90–13.57)	12.49 (11.95–13.22)	14.28 (13.36–15.37)	13.45 (12.83–14.21)	11.19 (10.20–12.25)	11.68 (11.12–12.47)
Eastern Sub–Saharan Africa	15.12 (14.52–15.86)	14.80 (14.30–15.53)	16.79 (16.12–17.59)	16.22 (15.64–16.99)	13.52 (12.88–14.29)	13.50 (13.03–14.21)

Southern Sub–Saharan Africa	8.75 (8.08–9.52)	8.58 (8.17–9.14)	11.52 (10.63–12.59)	10.61 (10.11–11.32)	6.42 (5.72–7.21)	6.93 (6.58–7.40)
Western Sub–Saharan Africa	12.27 (11.71–13.07)	12.41 (11.91–13.09)	14.36 (13.56–15.23)	14.23 (13.65–14.99)	10.08 (9.46–10.85)	10.78 (10.28–11.43)
South Asia	10.91 (10.28–11.67)	10.50 (10.07–11.08)	16.40 (15.41–17.46)	15.66 (15.02–16.44)	4.89 (4.12–5.72)	5.37 (5.08–5.74)
Andean Latin America	10.93 (10.25–11.84)	12.28 (11.76–13)	14.61 (13.77–15.58)	15.30 (14.65–16.13)	7.41 (6.68–8.29)	9.42 (8.96–10.05)
Caribbean	8.58 (8.10–9.25)	9.24 (8.85–9.77)	12.11 (11.48–12.88)	12.16 (11.66–12.80)	5.25 (4.78–5.89)	6.50 (6.15–6.92)
Central Latin America	10.79 (10.25–11.49)	10.92 (10.47–11.5)	15.88 (15.09–16.79)	15.20 (14.6–15.98)	6.00 (5.50–6.60)	7.09 (6.74–7.57)
Tropical Latin America	11.32 (10.5–12.26)	11.19 (10.67–11.89)	15.37 (14.13–16.69)	14.30 (13.65–15.14)	7.58 (6.60–8.69)	8.41 (7.96–8.97)
North Africa and Middle East	8.53 (8.15–9.06)	8.21 (7.87–8.71)	13.85 (13.23–14.6)	12.81 (12.27–13.56)	2.97 (2.76–3.24)	3.28 (3.14–3.49)
Central Asia	10.86 (10.36–11.48)	10.93 (10.50–11.52)	13.66 (13.01–14.43)	13.24 (12.71–13.95)	8.59 (8.04–9.22)	8.92 (8.54–9.44)
Central Europe	6.86 (6.53–7.37)	6.32 (6.03–6.81)	8.51 (8.05–9.15)	7.68 (7.32–8.31)	5.40 (5.09–5.88)	5.06 (4.80–5.45)
Eastern Europe	7.38 (6.94–7.92)	6.96 (6.62–7.45)	9.19 (8.61–9.87)	8.39 (7.95–8.98)	6.01 (5.53–6.58)	5.79 (5.49–6.30)
Australasia	6.84 (6.52–7.27)	6.94 (6.62–7.40)	9.04 (8.60–9.65)	8.41 (8.02–9.02)	4.76 (4.52–5.10)	5.54 (5.28–5.87)
High–income Asia Pacific	6.56 (6.23–7.08)	6.39 (6.08–6.94)	8.37 (7.89–9.08)	7.79 (7.39–8.45)	4.95 (4.64–5.45)	5.04 (4.78–5.50)
High–income North America	6.75 (6.40–7.25)	6.44 (6.10–6.92)	8.15 (7.71–8.78)	7.30 (6.90–7.89)	5.50 (5.20–5.93)	5.65 (5.36–6.10)
Southern Latin America	8.82 (8.21–9.58)	9.65 (9.18–10.31)	12.56 (11.72–13.50)	12.72 (12.09–13.51)	5.49 (4.80–6.22)	6.91 (6.54–7.41)
Western Europe	6.36 (6.06–6.78)	6.26 (5.97–6.71)	8.46 (8.06–9.07)	7.61 (7.26–8.23)	4.47 (4.24–4.83)	4.99 (4.76–5.31)

Table S2. The ASDR (2021) and EAPC (1990–2021) of occupational noise-induced hearing loss burden among 204 countries and territories.

Location	1990		2021		1990–2021
	Number, (95% UI)	ASDR per 100 000, (95% UI)	Number, (95% UI)	ASDR per 100 000, (95% UI)	EAPC, %, (95% CI)
Afghanistan	5,593.28 (3,784.77–7,985.78)	75.51 (51.28-107.09)	11,238.57 (7,600.53-15,923.46)	67.74 (46.91-94.30)	-0.34 (-0.35 to -0.33)
Albania	1,277.23 (843.84-1,854.29)	49.36 (32.75-71.13)	1,873.91 (1,246.90-2,661.44)	49.27 (33.05-70.02)	0 (-0.01 to 0.01)
Algeria	8,665.16 (5,920.01-12,229.89)	55.38 (37.66-78.98)	21,648.27 (14,646.97-31,093.88)	51.67 (35.00 - 73.91)	-0.20 (-0.25 to -0.14)
American Samoa	24.81 (16.33-36.13)	77.3 (51.06-111.84)	38.53 (25.46-55.13)	74.92 (49.88-105.48)	-0.16 (-0.18 to -0.14)
Andorra	17.4 (11.44-25.18)	28.04 (18.61-40.75)	37.42 (24.84-54.38)	27.9 (18.8-40.35)	-0.05 (-0.07 to -0.03)
Angola	5,961.22 (4,056.11-8,219.65)	104.25 (71.69-145.44)	18,378.97 (12,565.71-25,576.19)	100.74 (69.46-140.06)	-0.07 (-0.09 to -0.05)
Antigua and Barbuda	35.07 (23.10-49.10)	66.72 (43.68-93.69)	74.98 (49.48-105.50)	67.98 (45.30-95.32)	0.07 (0.06 to 0.07)
Argentina	16,892.30 (11,654.90-24,217.44)	52.08 (35.94-74.82)	27,578.94 (18,720.61-39,545.96)	52.64 (35.56-75.87)	0.06 (0.03 to 0.09)
Armenia	1,775.27 (1,187.73-2,534.27)	56.80 (38.02-81.24)	2,461.37 (1,624.90-3,456.86)	61.16 (40.78-86.41)	0.28 (0.23 to 0.32)
Australia	7,637.23 (5,160.80-10,880.43)	40.42 (27.38-57.73)	14,634.46 (9,726.84-20,852.32)	40.6 (26.93-57.86)	0.14 (0.04 to 0.24)
Austria	2,522.73 (1,658.14-3,623.94)	25.91 (17.22-37.37)	3,712.03 (2,474.54-5,420.17)	28.04 (18.52-41.21)	0.25 (0.20 to 0.30)
Azerbaijan	5,119.50 (3,358.67-	85.97 (56.38-	9,698.76 (6,402.54-	81.15 (53.91-	-0.21(-0.23 to

	7,252.55)	121.4)	13,903.64)	115.58)	-0.18)
Bahrain	261.31 (174.4-369.32)	73.55 (49.36-104.74)	1,200.62 (801.28-1,718.61)	75.36 (50.79-107.39)	0.10 (0.04 to 0.16)
Bangladesh	52,002.30 (35,160.52-72,807.11)	82.28 (56.10-115.47)	140,276.95 (96,407.64-196,705.26)	89.55 (61.55-125.52)	0.26 (0.25 to 0.28)
Barbados	166.48 (112.76-233.13)	64.27 (42.81-90.96)	284.1 (187.10-398.21)	64.3 (42.97-90.21)	0 (0 to 0.01)
Belarus	6,722.84 (4,479.01-9,446.11)	54.26 (36.64-76.05)	7,405.91 (4,975.39-10,499.61)	53.71 (36.4-76.39)	-0.06 (-0.10 to -0.01)
Belgium	3,265.58 (2,175.57-4,718.43)	25.2 (16.86-36.41)	4,460.24 (3,051.46-6,412.46)	25.92 (17.47-37.21)	0.12 (0.08 to 0.15)
Belize	71.57 (47.89-100.16)	64.23 (42.76-91.3)	238.98 (162.13-339.09)	64.78 (43.86-90.73)	0.02 (0 to 0.04)
Benin	2,976.34 (2,043.81-4,149.97)	116.02 (79.53-162.24)	8,252.88 (5,659.91-11,292.94)	109.34 (74.65-151.51)	-0.17 (-0.23 to -0.12)
Bermuda	45.94 (30.82-64.8)	68.99 (46.28-97.32)	74.76 (50.38-105.46)	71.76 (47.48-100.39)	0.14 (0.13 to 0.14)
Bhutan	388.4 (262.1-538.24)	108.81 (74.39-152.62)	770.44 (528.76-1,083.31)	108.02 (74.43-151.89)	-0.04 (-0.06 to -0.03)
Bolivarian Republic of Venezuela	8,292.48 (5,633.60-11,720.55)	65.98 (44.62-94.33)	19,572.59 (13,012.47-27,695.14)	63.99 (43.02-89.92)	0.91 (0.87 to 0.95)
Bosnia and Herzegovina	1,618.05 (1,075.50 - 2,313.74)	34.36 (23.04-49.01)	1,761.61 (1,180.11-2,529.74)	34.68 (23.4-49.55)	-0.02 (-0.05 to 0)
Botswana	442.78 (300.72-623.98)	57.67 (39.37-81.25)	1,289.00 (875.33-1,808.61)	61.06 (41.29-84.99)	0.27 (0.21 to 0.33)
Brazil	97,444.27 (65,165.44-138,126.20)	86.68 (57.74-121.32)	218,030.42 (144,190.40-309,856.06)	85.62 (56.90-121.24)	-0.06 (-0.14 to 0.02)
Brunei Darussalam	57.03 (37.91-82.84)	34.33 (23.14-48.85)	153.31 (101.72-224.25)	32.74 (21.9-47.15)	-0.13 (-0.14 to -0.12)

Bulgaria	5,607.73 (3,763.29-7,947.41)	49.21 (32.67-70.16)	5,112.07 (3,412.06-7,348.19)	46.24 (30.77-65.96)	-0.19 (-0.2 to -0.18)
Burkina Faso	6,422.38 (4,386.47-8,973.85)	116.68 (80.67-164.17)	14,168.41 (9,742.65-19,515.72)	107.62 (74.53-149.63)	-0.23 (-0.31 to -0.16)
Burundi	5,023.30 (3,500.76-7,015.56)	164.21 (113.35-230.04)	12,496.18 (8,649.3-17,158.39)	169.25 (118.18-235.28)	0.11 (0.01 to 0.20)
Cambodia	9,059.77 (6,170.19-12,535.04)	145.29 (99.41-200.87)	22,835.60 (15,580.20-31,680.38)	147.65 (100.90-205.13)	0.01 (0 to 0.03)
Cameroon	6,207.21 (4,243.66-8,624.53)	101.32 (69.58-141.18)	19,327.42 (13,157.51-26,615.87)	99.09 (67.90-138.21)	-0.11 (-0.16 to -0.06)
Canada	14,030.93 (9,586.63-20,075.32)	45.08 (30.78-64.24)	24,861.31 (16,678.69-35,600.46)	44.08 (30.15-63.11)	-0.09 (-0.12 to -0.07)
Central African Republic	1,544.07 (1,057.99-2,160.27)	97.46 (67.16-136.94)	3,255.43 (2,214.19-4,488.61)	97.48 (66.92-134.00)	0.01 (0 to 0.01)
Chad	3,179.4 (2,171.93-4,458.58)	93.38 (64.38-132.29)	8,264.49 (5,637.94-11,431.29)	95.57 (66.24-133.31)	0.11 (0.05 to 0.17)
Chile	5,178.12 (3,450.63-7,341.63)	45.55 (30.54-64.69)	12,823.80 (8,576.32-18,066.06)	53.56 (35.89-75.68)	0.51 (0.5 to 0.52)
China	1,279,684.59 (862,566.92-1,794,996.76)	123.08 (83.02-173.74)	2,683,891.80 (1,804,391.74-3,813,460.13)	133.99 (90.16-188.50)	0.32 (0.30 to 0.35)
Colombia	14,932.31 (10,103.82-21,188.95)	65.36 (43.77-92.28)	36,617.59 (24,213.69-51,853.03)	66.5 (44.16-94.09)	0.04 (0.02 to 0.07)
Commonwealth of the Bahamas	112.44 (75.12-159.51)	55.68 (37.24-80.38)	287.92 (192.23-411.11)	65.05 (43.96-92.02)	0.54 (0.52 to 0.56)
Comoros	247.53 (170.73-349.12)	98.49 (67.75-139.39)	596.53 (407.77-849.42)	99.45 (67.09-140.19)	0.03 (-0.05 to 0.12)
Congo	1,223.44 (822.6-1,706.62)	87.34 (58.83-121.98)	3,267.63 (2,243.57-4,599.03)	84.5 (58.24-118.04)	-0.10 (-0.12 to -0.07)

Cook Islands	12.81 (8.34-18.02)	85.38 (56.25-119.96)	19.37 (12.68-27.36)	85.28 (56.58-119.51)	-0.10 (-0.14 to -0.05)
Costa Rica	1,431.42 (954.32-2,028.20)	65.21 (43.55-92.14)	3,501.88 (2,344.03-4,983.2)	63.87 (42.82-90.85)	-0.06 (-0.07 to -0.05)
Croatia	2,399.72 (1,589.69-3,452.48)	39.37 (25.95-55.76)	2,676.98 (1,810.97-3,772.06)	41.19 (27.97-58.13)	0.18 (0.15 to 0.21)
Cuba	6,899.72 (4,575.46-9,822.58)	64.64 (42.89-92.79)	10,859.48 (7,187.19-15,466.39)	66.3 (44.48-94.22)	0.08 (0.05 to 0.11)
Cyprus	217.5 (146.03-311.38)	26.14 (17.63-37.51)	478.91 (318.06-688.46)	25.47 (16.9-36.79)	-0.07 (-0.10 to -0.04)
Czech Republic	7,135.40 (4,837.73-10,042.46)	58.07 (39.36-81.77)	8,756.72 (5,836.82-12,343.29)	53.28 (35.41-75.14)	-0.29 (-0.31 to -0.28)
Democratic People's Republic of Korea	14,119.36 (9,270.91-20,140.19)	72.92 (48.75-104.10)	25,523.81 (16,647.72-36,209.62)	76.39 (50.06-107.91)	0.15 (0.11 to 0.18)
Democratic Republic of the Congo	24,386.64 (16,335.79-34,255.38)	111.85 (76.7-154.46)	58,363.17 (40,061.35-81,676.66)	108.55 (74.73-150.28)	-0.09 (-0.10 to -0.08)
Denmark	1,787.45 (1,182.88-2,587.07)	27.17 (17.97-39.78)	2,360.45 (1,555.01-3,385.77)	27.52 (18.3-40.28)	0.05 (-0.01 to 0.11)
Djibouti	229.02 (156.24-315.01)	103.48 (70.60-144.84)	995.53 (676.82-1,388.28)	103.75 (71.42-145.27)	-0.01 (-0.10 to 0.07)
Dominica	34.79 (23.48-49.66)	58.6 (39.14-83.76)	49.99 (33.26-70.47)	61.03 (40.52-85.82)	0.18 (0.16 to 0.20)
Dominican Republic	2,918.83 (1,973.30-4,096.31)	61.52 (41.43-86.41)	6,995.36 (4,702.52-9,925.08)	65.01 (43.42-92.20)	0.20 (0.19 to 0.20)
Ecuador	4,535.70 (3,071.40-6,321.23)	67.9 (46.26-95.28)	12,788.65 (8,614.58-17,798.80)	73.53 (49.59-102.27)	0.47 (0.36 to 0.59)
Egypt	23,471.09 (16,017.26-33,612.09)	65.3 (45.04-92.98)	53,766.91 (36,793.42-76,665.25)	65.14 (44.56-92.77)	0.03 (0.01 to 0.04)
El Salvador	1,987.01 (1,336.13-	57.14 (38.46-	3,748.32 (2,486.43-	60.6 (40.12-	0.23 (0.19 to

	2,785.12)	80.7)	5,248.83)	85.03)	0.26)
Equatorial Guinea	237.35 (160.69-333.02)	95.55 (65.33-132.66)	844.97 (576.45-1,172.94)	96.35 (66.21-134.22)	0.04 (0 to 0.08)
Eritrea	2,595.18 (1,770.01-3,603.90)	144.49 (99.06-197.8)	6,426.36 (4,369.15-9,010.11)	148.51 (102.61-206.03)	0.06 (-0.04 to 0.16)
Estonia	1,182.22 (787.87-1,669.42)	62.41 (42.19-88.21)	1,105.78 (743.11-1,576.19)	55.16 (36.8-79.41)	-0.42 (-0.43 to -0.41)
Ethiopia	42,387.89 (29,491.46-58,811.85)	151.3 (104.74-208.8)	101,183.64 (69,529.81-139,008.79)	154.62 (107.24-214.5)	0.06 (-0.02 to 0.13)
Federated States of Micronesia	45.35 (30.97-64.27)	72.94 (49.89-104.61)	69.66 (46.26-99.59)	74.88 (49.96-106.95)	0.03 (-0.01 to 0.06)
Fiji	388.63 (258.45-556.27)	74.72 (49.47-106.63)	651.09 (434.65-933.8)	72.53 (48.45-103.02)	-0.18 (-0.21 to -0.15)
Finland	2,019.07 (1,369.31-2,881.96)	31.46 (21.3-45.26)	2,699.21 (1,844.55-3,904.39)	29.42 (19.97-42.00)	-0.09 (-0.15 to -0.03)
France	19,273.58 (12,956.80-27,578.63)	27.93 (18.63-40.41)	27,454.21 (18,629.78-38,762.65)	28.1 (18.72-40.40)	0.05 (0.01 to 0.09)
Gabon	525.03 (356.36-734.61)	80.11 (54.45-111.76)	998.19 (677.70-1403.76)	74.11 (50.58-103.53)	-0.25 (-0.29 to -0.22)
Georgia	4,425.70 (2,893.99-6,283.71)	70.88 (46.68-100.02)	3,603.91 (2,399.51-5,168.64)	70.29 (47.18-100.04)	-0.04 (-0.05 to -0.02)
Germany	30,482.62 (20,571.9-43,731.44)	28.73 (19.39-41.05)	39,403.36 (26,522.15-56,582.21)	29.19 (19.29-41.6)	0.01 (-0.02 to 0.05)
Ghana	8,698.91 (5,832.04-12,157.83)	100.25 (67.67-140.39)	21,756.5 (14,931.46-30,574.75)	92.28 (62.66-129.94)	-0.24 (-0.31 to -0.17)
Greece	3,998.54 (2,701.42-5,662.48)	29.5 (19.95-41.55)	4,445.05 (3,040.70-6,367.50)	25.92 (17.64-36.58)	-0.40 (-0.46 to -0.34)
Greenland	22.24 (15.12-31.73)	46.59 (31.76-67.01)	33.41 (22.29-48.27)	45.04 (30.6-65.59)	-0.11 (-0.13 to -0.08)

Grenada	40.58 (27.48-56.70)	60.54 (40.84-85.14)	77.63 (52.18-110.11)	65.11 (44.28-91.73)	0.25 (0.23 to 0.28)
Guam	83.1 (55.49-119.78)	75.69 (50.62-107.43)	140.34 (92.38-199.8)	71.85 (47.5-102.34)	-0.24 (-0.27 to -0.21)
Guatemala	3,490.86 (2,338.09-4,834.28)	75.06 (50.32-104.42)	9,195.4 (6,185.52-12,769.25)	71.35 (47.78-99.27)	-0.16 (-0.18 to -0.14)
Guinea	4,043.12 (2,738.94-5,596.33)	103.06 (70.00-144.04)	7,822.53 (5,268.44-10,775.77)	98.74 (67.4-138.39)	-0.13 (-0.18 to -0.08)
Guinea-Bissau	504.47 (337.38-709.95)	93.02 (62.18-131.73)	1,073.6 (726.29-1,484.49)	91.38 (62.35-127.24)	-0.03 (-0.08 to 0.02)
Guyana	308.93 (204.22-434.94)	60.5 (40.16-86.15)	447.1 (303.79-627.44)	60.73 (41.41-84.66)	0.01 (0 to 0.02)
Haiti	2,223.38 (1,485.14-3,117.1)	55.37 (37.17-77.88)	5,703.6 (3,797.8-8,089.2)	59.57 (39.64-83.03)	0.25 (0.20 to 0.30)
Honduras	1,285.41 (867.64-1,794.64)	47.4 (32.17-66.47)	5,184.89 (3,463.72-7,361.14)	64.37 (42.54-91.77)	0.99 (0.95 to 1.03)
Hungary	6,448.34 (4,316.22-9,120.12)	49.54 (33.56-69.7)	6,891.07 (4,544.09-9,853.35)	46.35 (30.79-65.6)	-0.28 (-0.32 to -0.25)
Iceland	91.63 (62.22-130.90)	34.1 (23.06-48.51)	159.69 (108.26-228.89)	33.57 (22.71-48.06)	-0.06 (-0.08 to -0.04)
India	632,742.65 (430,525-889,208.15)	102.84 (69.67-143.07)	1,319,576.38 (896,379.27-1,831,832.80)	96.37 (65.17-134.57)	-0.36 (-0.43 to -0.30)
Indonesia	157,673.99 (107,781.98-219,775.06)	117.88 (80.68-165.46)	330,257.42 (224,167.60-464,041.64)	113.43 (77.12-158.02)	-0.28 (-0.32 to -0.23)
Iraq	6,231.59 (4,269.09-8,797.23)	60.03 (41.39-85.19)	18,566.23 (12,629.37-26,428.09)	57.15 (39.07-80.82)	-0.15 (-0.16 to -0.13)
Ireland	953.16 (645.03-1,366.20)	25.54 (17.13-36.76)	1,721.16 (1,155.47-2,502.26)	26.12 (17.48-37.54)	0.07 (0.06 to 0.09)
Islamic Republic of Iran	21,995.83 (15,238.22-	62.77 (43.47-89.19)	54,883.57 (36,970.88-	59.86 (40.70-84.80)	-0.18 (-0.19 to -0.17)

	31,318.49)		77,791.99)		
Israel	1,075.75 (705.41-1,549.24)	23.15 (15.27-33.22)	2,735.66 (1,814.56-3,932.17)	25.82 (16.87-37.18)	0.39 (0.37 to 0.41)
Italy	22,480.26 (15,271.96-31,660.01)	29.78 (20.1-41.98)	29,941.46 (20,445.85-42,689.12)	29.14 (19.8-41.05)	-0.05 (-0.08 to -0.02)
Jamaica	1,248.39 (841.27-1,756.76)	68.21 (45.03-96.30)	2,098.16 (1,394.24-2,953.68)	67.46 (44.79-95.00)	-0.06 (-0.08 to -0.05)
Japan	59,745.25 (39,777.20-85,814.14)	37.00 (24.64-52.57)	82,104.84 (55,887.38-116,544.98)	37.44 (24.86-53.51)	0.02 (0 to 0.03)
Jordan	1,063.21 (699.99-1,533.55)	52.95 (35.19-75.47)	5,196.69 (3,538.22-7,492.70)	50.52 (34.27-72.21)	-0.17 (-0.19 to -0.16)
Kazakhstan	11,604.40 (7,684.91-16,360.58)	80.01 (53.15-113.44)	15,911.70 (10,547.88-22,155.17)	80.1 (53.42-110.84)	0 (0 to 0.01)
Kenya	17,415.48 (11,738.48-24,277.86)	151.81 (102.89-209.49)	53,999.54 (37,388.90-74,203.51)	162.52 (110.79-223.47)	0.21 (0.1 to 0.31)
Kingdom of Eswatini	234.73 (155.93-329.77)	56.84 (37.53-80.12)	426.14 (284.34-594.56)	52.77 (35.03-74.29)	-0.22 (-0.23 to -0.21)
Kiribati	15.81 (10.5-23.33)	31.92 (21.26-46.81)	42.36 (28.05-59.79)	42.47 (28.20-60.32)	0.90 (0.72 to 1.08)
Kuwait	923.60 (635.26-1,301.94)	78.70 (53.18-113.46)	3,748.73 (2,549.50-5,337.87)	78.18 (53.49-110.73)	-0.05 (-0.06 to -0.03)
Kyrgyzstan	2,566 (1,716.20-3,613.47)	75.11 (50.39-105.97)	4,497 (2,997.51-6,327.58)	75.48 (50.53-106.06)	0.02 (0.01 to 0.03)
Lao People's Democratic Republic	4,223.44 (2,886.24-5,884.54)	155.72 (106.72-216.49)	9,244.3 (6,282.73-12,938.68)	149.21 (101.81-207.20)	-0.22 (-0.26 to -0.19)
Latvia	1,655.05 (1,107.10-2,324.31)	50.35 (33.96-70.77)	1,440.51 (960.56-2,030.05)	49.72 (33.78-70.31)	-0.04 (-0.05 to -0.03)
Lebanon	1,607.89 (1,090.96-2,288.09)	64.74 (44.13-91.33)	3,619.36 (2,465.46-5,084.01)	60.88 (41.09-85.79)	-0.23 (-0.25 to -0.21)

Lesotho	664.92 (449.28-939.88)	66.18 (45.04-93.02)	954 (645.94-1,332.54)	66.75 (44.97-93.53)	0.08 (0.06 to 0.09)
Liberia	1,481.59 (991.88-2,046.52)	101.5 (68.50-141.92)	3,234.53 (2,165.45-4,493.88)	94.9 (65.61-132.84)	-0.18 (-0.22 to -0.15)
Libya	1,601.68 (1,096.96-2,286.93)	65.86 (45.29-94.31)	3,766.69 (2,557.64-5,394.32)	55.37 (37.61-78.97)	-0.59 (-0.60 to -0.58)
Lithuania	2,126.04 (1,420.99-2,968.93)	49.69 (33.4-69.51)	2,099.76 (1,396.69-2,975.89)	49.19 (32.87-70.07)	-0.05 (-0.06 to -0.04)
Luxembourg	115.9 (76.93-168.77)	23.92 (15.85-34.86)	209.56 (138.26-303.98)	22.87 (15.09-33.2)	-0.16 (-0.18 to -0.14)
Madagascar	14,934.04 (10,255.53-20,315.42)	213.57 (146.8-292.62)	38,840.32 (26,390.22-52,995.5)	216.77 (148.91-297.71)	-0.01 (-0.13 to 0.12)
Malawi	8,653.63 (5,841.30-11,920.81)	159.79 (107.52-222.23)	18,541.81 (12,692.08-25,708.95)	162.01 (113.41-224.12)	0.06 (0.04 to 0.08)
Malaysia	14,902.32 (10,098.13-20,852.04)	117.59 (80.90-164.95)	37,193.88 (25,238.53-52,057.1)	114.04 (77.42-160.17)	-0.19 (-0.22 to -0.17)
Maldives	145.42 (98.61-202.25)	115.69 (79.23-161.45)	582.79 (391.62-818.41)	111.44 (76.28-157.19)	-0.23 (-0.28 to -0.18)
Mali	5,399.35 (3,601.59-7,460.23)	102.87 (68.72-141.9)	13,190.51 (8,958.55-18,108.08)	99.31 (68.36-137.2)	-0.09 (-0.13 to -0.04)
Malta	108.52 (71.93-157.11)	25.98 (17.21-37.44)	178.01 (120.95-257.41)	25.99 (17.40-37.43)	-0.05 (-0.07 to -0.03)
Marshall Islands	14.10 (9.11-19.79)	60.72 (39.81-85.00)	27.11 (18.03-38.54)	57.37 (38.35-80.94)	-0.25 (-0.28 to -0.22)
Mauritania	930.91 (626.58-1,313.11)	75.44 (51.49-106.55)	1,885.27 (1,298.53-2,647.61)	68.4 (47.02-96.29)	-0.33 (-0.39 to -0.26)
Mauritius	927.97 (629.59-1,296.27)	99.38 (67.39-140.95)	1,754.4 (1,154.19-2,468.04)	100.85 (67.07-139.39)	-0.04 (-0.06 to -0.01)
Mexico	40,166.09 (27,278.89-	71.23 (48.29-100.1)	95,617.81 (64,292.18-	70.8 (47.83-99.68)	-0.04 (-0.06 to -0.03)

	56,785.17)		134,838.85)		
Mongolia	947.61 (634.8-1,334.83)	70.63 (47.48-100.04)	2,136.94 (1,422.62-3,062.09)	70.82 (47.45-99.76)	0.04 (0.03 to 0.05)
Montenegro	224.04 (150.47-320.66)	34.26 (22.85-48.89)	292.52 (193.51-419.91)	33.68 (22.59-47.87)	-0.13 (-0.17 to -0.09)
Morocco	11,920.72 (8,109.77-16,995.22)	68.87 (46.71-98.25)	25,004.69 (17,078.32-35,299.44)	65.97 (45.08-93.19)	-0.09 (-0.11 to -0.08)
Mozambique	11,624.05 (8,088.48-16,117.58)	147.02 (102.09-202.69)	25,753.71 (17,760.93-35,551.39)	152.85 (105.06-209.56)	0.10 (0 to 0.20)
Myanmar	29,536.47 (20,122.88-40,708.71)	96.57 (67.00-133.86)	67,764.01 (45,624.09-94,406.00)	119.87 (80.79-167.29)	0.70 (0.68 to 0.72)
Namibia	494.45(328.63-695.33)	58.43 (38.86-82.62)	1,068.78 (715.42-1,520.85)	57.26 (38.34-81.24)	-0.03 (-0.07 to 0.01)
Nepal	18,579.56 (12,899.05-25,230.80)	144.64 (100.15-195.35)	34276.34 (23843.65-47993.17)	126.49 (87.77-176.05)	-0.38 (-0.43 to -0.33)
Netherlands	3,920.43 (2,584.34-5,687.17)	21.85 (14.37-31.76)	6,797.08 (4,496.79-9,858.22)	26.92 (17.72-39.14)	0.73 (0.66 to 0.80)
New Zealand	1,644.73 (1,101.07-2,368.66)	44.12 (29.52-63.42)	3,095.39 (2,066.75-4,427.35)	43.62 (29.2-62.59)	-0.04 (-0.05 to -0.03)
Nicaragua	1,248.86 (839.07-1,786.34)	59.47 (39.81-84.47)	3,855.41 (2,635.66-5,451.34)	65.59 (44.26-92.24)	0.36 (0.31 to 0.40)
Niger	4426.95 (2975.57-6126.80)	105.94 (72.46-147.18)	12,635.95 (8,502.68-17,536.19)	99.97 (68.33-138.55)	-0.17 (-0.22 to -0.11)
Nigeria	55,021.7 (38,066.88-76,523.62)	99.69 (67.9 - 139.13)	133910.38 (92008.66 - 185097.58)	99.38 (68.78 - 139.08)	-0.03 (-0.09 to 0.04)
North Macedonia	731.2 (496.22-1,033.6)	35.86 (24.38-50.41)	1,154.90 (771.55-1,660.19)	36.53 (24.69-51.96)	0.01 (-0.03 to 0.05)
Northern Mariana Islands	25.18 (16.44-36.25)	73.74 (48.98-104.58)	48.7 (32.07-70.18)	82.64 (54.31-117.03)	0.34 (0.32 to 0.36)

Norway	1,879.75 (1,279.70-2,679.19)	34.75 (23.35-49.32)	2,690.60 (1,819.99-3,820.27)	34.53 (22.98-48.76)	0.08 (0.05 to 0.11)
Oman	708.43 (473.58-1,010.99)	61.06 (40.48-86.41)	2,566.78 (1,727.32-3,664.09)	61.23 (41.52-88.33)	0.03 (-0.07 to 0.14)
Pakistan	48,927.75 (33,496.89-69,082.89)	69.42 (47.53-97.99)	137,595.42 (93,033.47-193,031.35)	78.08 (53.25-109.28)	0.39 (0.35 to 0.42)
Palestine	585.71 (400.1-828.93)	52.53 (35.62-74.61)	1,831.18 (1,252.62-2,581.31)	51.46 (34.99-72.23)	-0.11 (-0.13 to -0.08)
Panama	1,093.59 (722.11-1,529.92)	61.74 (40.19-87.02)	2,838.16 (1,870.19-4,009.84)	64.40 (42.43-91.00)	0.13 (0.10 to 0.16)
Papua New Guinea	1,856.43 (1,260.20-2,609.49)	70.99 (48.73-99.94)	5,249.01 (3,515.75-7,405.02)	70.44 (48.11-98.98)	-0.09 (-0.11 to -0.07)
Paraguay	2,157.4 (1,416.66-3,081.57)	80.55 (53.70-116.67)	5,630.9 (3,752.41-8,019.39)	85.2 (56.49-121.57)	0.21 (0.19 to 0.22)
Peru	9,162.62 (6,124.83-12,821.66)	61.41 (41.26-85.84)	22,883.52 (15,191.29-31,823.43)	63.5 (42.1-88.35)	0.18 (0.15 to 0.21)
Philippines	49,980.7 (33,980.03-69,407.13)	122.27 (83.64-170.56)	115,811.68 (80,107.89-162,403.02)	114.89 (78.68-161.95)	-0.26 (-0.28 to -0.24)
Plurinational State of Bolivia	2,155.16 (1,491.51-3,026.22)	52.53 (36.16-73.23)	7,302.18 (4,853.46-10,257.06)	68.30 (45.50-95.60)	-0.06 (-0.07 to -0.04)
Poland	23,232.64 (15,632.79-32,880.51)	54.64 (36.93-76.94)	30,094.08 (20,078.16-42,359.86)	52.01 (35.23-73.27)	-0.22 (-0.24 to -0.2)
Portugal	3,944.90 (2,704.37-5,645.19)	31.7 (21.40-45.24)	5,053.47 (3,444.31-7,180.89)	28.69 (19.44-41.25)	-0.34 (-0.37 to -0.31)
Principality of Monaco	13.47 (9.05-19.45)	27.93 (18.75-40.44)	17.71 (11.87-25.51)	26.83 (17.96-38.38)	-0.15 (-0.18 to -0.12)
Puerto Rico	927.94 (626.61-1,327.79)	25.89 (17.47-37.07)	1,390.87 (928.21-1,957.00)	27.78 (18.61-39.18)	0.20 (0.17 to 0.24)
Qatar	313.83 (209.19-	98.31 (66.14-	2,707.05 (1,827.57-	95.54 (64.85-	0.03 (-0.01 to

	441.16)	139.01)	3,861.07)	134.35)	0.07)
Republic of Cabo Verde	177.44 (121.32-248.25)	79.53 (55.04-113.25)	406.52 (277.15-570.59)	76.35 (51.79-107.08)	-0.11 (-0.16 to -0.06)
Republic of Côte d'Ivoire	6,505.82 (4,496.78-9,016.)	99.68 (68.23-140.47)	16,262.85 (11144.56-22521.13)	93.34 (64.27-130.41)	-0.18 (-0.24 to -0.13)
Republic of Korea	13,365.25 (8,997.63 - 19,324.47)	34.81 (23.31-49.86)	27,614.6 (18,498.09-39,945.6)	32.56 (21.67-46.6)	-0.24 (-0.26 to -0.23)
Republic of Moldova	2,212.08 (1,524.52-3,146.34)	47.87 (33.06-67.97)	2,365.93 (1,597.15-3,332.38)	43.41 (29.58-61.5)	-0.34 (-0.40 to -0.29)
Republic of Nauru	4.99 (3.23 - 7.14)	76.16 (50.68-108.34)	6.19 (4.09-8.83)	76.96 (50.51-109.88)	0.02 (-0.02 to 0.05)
Republic of Niue	1.53 (1.01-2.20)	74.94 (49.28-107.88)	1.56 (1.03-2.22)	76.04 (50.44-108.3)	-0.01 (-0.03 to 0.01)
Republic of Palau	9.20 (6.12-13.17)	74.48 (49.94-107.89)	19.03 (12.49-27.17)	76.76 (50.73-107.88)	0 (-0.04 to 0.04)
Republic of San Marino	9.14 (6.16-13.03)	30.26 (20.48-43.57)	15.38 (10.36-22.05)	28.65 (19.04-41.38)	-0.21 (-0.23 to -0.19)
Republic of the Gambia	472.17 (315.85-661.7)	91.06 (62.03-127.57)	1,169.4 (785.56-1,620.92)	81.71 (55.38-114.86)	-0.32 (-0.37 to -0.28)
Romania	16,487.93 (10,972.87-23,559.20)	61.18 (41.28-86.24)	16,309.92 (10,849.39-22,845.21)	55.31 (37.15-78.53)	-0.40 (-0.44 to -0.35)
Russian Federation	105,113.37 (70,103.9 - 149,697.75)	59.05 (39.63-83.87)	117,396.67 (78,064.10-166,351.40)	55.71 (37.46-78.56)	-0.18 (-0.19 to -0.16)
Rwanda	6,326.6 (4,342.24-8,734.67)	161.54 (110.91-224.91)	13,835.46 (9,510.02-19,150.62)	156.52 (107.15-216.45)	-0.08 (-0.17 to 0.02)
Saint Kitts and Nevis	26.82 (18.09-37.67)	75.45 (50.9-106.19)	54.65 (36.11-77.26)	71.91 (48.11-101.49)	-0.17 (-0.19 to -0.15)
Saint Lucia	64.13 (42.57-90.00)	65.9 (43.35-92.75)	149.06 (99.25-209.69)	63.98 (43.09-89.90)	-0.09 (-0.1 to -0.08)

Saint Vincent and the Grenadines	46.98 (31.31-67.17)	60.37 (40.32-85.83)	84.86 (56.95-119.87)	61.45 (41.16-86.46)	0.06 (0.06 to 0.07)
Samoa	71.54 (47.19-101.38)	66.51 (43.56-94.5)	105.28 (70.02-149.37)	63.05 (41.81-89.31)	-0.24 (-0.29 to -0.2)
Sao Tome and Principe	56.84 (38.09-79.8)	75.8 (51.38-105.51)	98.39 (66.9-137.79)	63.17 (42.84-87.82)	-0.66 (-0.74 to -0.59)
Saudi Arabia	5,937.08 (3,974.92-8,521.41)	67.31 (45.47-95.53)	21,335.98 (14,296.63-30,458.28)	62.36 (42.5-88.89)	-0.25 (-0.3 to -0.21)
Senegal	3,912.54 (2,713.09-5,421.2)	91.11 (63.13-127.71)	8,436.61 (5,718.98-11,776.07)	80.93 (54.81-114.95)	-0.36 (-0.43 to -0.28)
Serbia	5,038.45 (3,328.46-7,256.3)	42.7 (28.51-61.12)	5,585.08 (3,665.93-7,918.29)	42.28 (28.02-60.37)	-0.08 (-0.1 to -0.06)
Seychelles	70.48 (46.94-99.75)	116.34 (77.31-165.29)	144.21 (96.49-203.57)	114.96 (77.16-160.74)	-0.10 (-0.13 to -0.08)
Sierra Leone	2,690.12 (1,821.18-3,720.83)	106.12 (71.81-148.59)	5,106.9 (3,474.31-7,091.63)	96.89 (66.01-135.6)	-0.26 (-0.33 to -0.19)
Singapore	911.63 (616.94-1,311.74)	31.5 (21.34-45.16)	2,728.39 (1,839.12 - 3,964.23)	32.28 (21.79-46.68)	0.10 (0.06 to 0.13)
Slovakia	2,956.71 (1,996.05-4,251.41)	51.67 (34.8-74.39)	3,933.56 (2,596.52-5,641.6)	48.28 (32.06-69.38)	-0.28 (-0.31 to -0.26)
Slovenia	1,095.28 (728.74-1,569.11)	47.09 (31.56-67.38)	1,503.31 (1,005.97-2,138.42)	46.64 (31.4-66.85)	-0.04 (-0.05 to -0.03)
Socialist Republic of Viet Nam	66,563.42 (44,527.13-91,930.97)	136.18 (92.07-188.71)	154,675.04 (104,241.49-217,559.3)	139.77 (94.62-196.91)	0.03 (0.01 to 0.05)
Solomon Islands	116.76 (75.71-166.80)	60.48 (39.70-86.15)	416.89 (277.7-588.54)	82.15 (54.74-117.19)	1.08 (0.89 to 1.27)
Somalia	5,414.94 (3,654.65-7,568.53)	139.6 (95.8-196.55)	14,990.08 (10,045.92-20,654.08)	144.7 (99.67-200.25)	0.11 (0.02 to 0.20)
South Africa	15,274.38 (10,276.19-	58.51 (39.68-	31,528.61 (21,405.92-	56.85 (38.77-	-0.01 (-0.05 to

	21,465.10)	81.56)	44,390.02)	79.48)	0.02)
South Sudan	4,985.9 (3,463.50-6,810.20)	146.38 (101.24-203.68)	8,468.62 (5,817.10-11,817.46)	147.9 (102.21-205.81)	0.01 (-0.06 to 0.08)
Spain	12,994.25 (8,818.36-18,567.41)	27.12 (18.45-38.8)	18,902.95 (12,756.84 - 27,158.65)	25.98 (17.5-37.33)	-0.09 (-0.12 to -0.06)
Sri Lanka	11,858.66 (7,942.24-16,547.43)	86.18 (57.80-120.27)	24,865.44 (16,691.18-34,716.87)	94.32 (63.62-130.83)	0.18 (0.13 to 0.23)
Sudan	8,280.82 (5,551.85-11,966.97)	71.34 (47.93-102.35)	18,656.91 (12,797.06-26,494.34)	69.16 (47.16-99.03)	-0.10 (-0.12 to -0.08)
Suriname	168.24 (110.96-242.34)	56.09 (37.14-80.46)	371.84 (249.28-523.07)	57.49 (38.75-80.49)	0.11 (0.07 to 0.14)
Sweden	3,305.03 (2,209.36-4,759.68)	29.29 (19.51-42.16)	4,051.32 (2,715.66-5,842.65)	27.09 (17.9-39.12)	-0.35 (-0.41 to -0.29)
Switzerland	2,469.71 (1,654.82-3,590.29)	28.27 (19.04-41.09)	3,810.58 (2,536.07-5,472.01)	28.83 (19.29-42.12)	0.02 (-0.02 to 0.05)
Syrian Arab Republic	5,220.64 (3,475.50-7,440.7)	74.73 (50.51-106.8)	9,230.44 (6,212.13-13,139.18)	62.95 (42.35-89.76)	-0.59 (-0.67 to -0.51)
Taiwan (Province of China)	11,739.43 (7,767.03-17,285.23)	61.43 (40.66-90.29)	22,221.47 (14,703.08-32,561.63)	61.35 (40.47-89.52)	-0.02 (-0.09 to 0.04)
Tajikistan	2,000.66 (1,345.08-2,862.07)	59.24 (39.82-85.82)	4,627.18 (3,054.53-6,655.16)	58.85 (39.24-83.88)	-0.04 (-0.06 to -0.02)
Thailand	61,293.1 (41,621.62-84,936.04)	130.2 (88.54-182.01)	125,212.85 (83,768.88-176,656.48)	127.45 (85.94-178.99)	-0.13 (-0.15 to -0.11)
Timor-Leste	492.41 (333.11-693.95)	108.85 (72.73-155.64)	1,058.7 (720.95-1,475.66)	105.16 (71.92-146.8)	-0.21 (-0.23 to -0.18)
Togo	2,038.35 (1,388.88-2,791.63)	107.41 (74.77-148.32)	5,592.74 (3,825.18-7,787.32)	98.77 (67.44-138.39)	-0.23 (-0.28 to -0.18)
Tokelau	0.97 (0.65-1.37)	71.18 (47.88-100.61)	1.04 (0.69-1.49)	73.75 (48.54-105.22)	0.05 (0.03 to 0.08)

Tonga	41.32 (27.73-58.83)	62.34 (42.13-88.06)	58.80 (39.50-82.57)	68.03 (45.56-95.82)	0.16 (0.11 to 0.20)
Trinidad and Tobago	620.53 (419.21-874.93)	64.13 (42.57-90.22)	1,196.8 (793.1-1,688.57)	66.23 (43.94-92.89)	0.15 (0.13 to 0.17)
Tunisia	4,168.24 (2,832.43-5,899.64)	68.99 (47.07-96.95)	8,583.32 (5,751.69-12,157.47)	62.12 (41.82-87.82)	-0.34 (-0.36 to -0.33)
Turkey	36,985.17 (25,449.21-51,502.04)	83.95 (57.95-117.27)	69,066.48 (46,474.91-98,554.41)	71.72 (48.61-101.75)	-0.58 (-0.60 to -0.55)
Turkmenistan	1,403.29 (930.77-2,001.22)	57.84 (38.49-82.69)	2,940.09 (1,924.10-4,188.59)	61.00 (40.28-86.46)	0.16 (0.14 to 0.18)
Tuvalu	5.72 (3.8 -8.17)	72.61 (49.17-103.83)	8.80 (5.90- 12.57)	76.31 (51.11-108.86)	0.11 (0.09 to 0.13)
Uganda	10,759.2 (7,452.01-15,071.13)	121.11 (84.32-168.06)	27,850.76 (18,926.96-38,817.31)	121.98 (83.12-169.39)	0.02 (-0.10 to 0.14)
Ukraine	36,963.18 24,829.11-52,578.87)	55.94 (37.85-79.30)	35,731.22 (24,020.45-51,022.42)	54.16 (36.55-76.84)	-0.11 (-0.12 to -0.09)
United Arab Emirates	1,093.49 (731.99-1,563.39)	85.97 (58.41-123.19)	9,478.99 (6,282.55-13,842.22)	90.5 (62.27-127.65)	0.12 (0.08 to 0.16)
United Kingdom of Great Britain and Northern Ireland	25,623.77 (17,206.33-36,593.39)	34.72 (23.42-49.76)	33,486.85 (22,393.29-47,784.32)	34.23 (22.89-49.46)	0.36 (0.25 to 0.47)
United Republic of Tanzania	20,297.28 (13,739.04-28,000.12)	139.9 (94.65-193.23)	49,740.04 (34,091.05-68,955.41)	139.3 (96.22-191.29)	0.01 (-0.08 to 0.10)
United States of America	149,180.41 (102,028.86-209,564.22)	51.78 (35.37-72.49)	227,030.44 (153,877.39-319,544.42)	47.18 (32.25-66.17)	-0.49 (-0.62 to -0.36)
United States Virgin Islands	61.34 (40.94-87.09)	62.12 (41.39-87.68)	88.84 (59.57-126.13)	64.16 (43.33-91.51)	0.12 (0.11 to 0.13)
Uruguay	1,816.77 (1,229-	51.12 (34.27-	2,502.16 (1,668.28-	56.21 (37.20-	0.34 (0.32 to

	2,625.09)	73.95)	3,564.91)	80.14)	0.37)
Uzbekistan	10,426.28 (7,045.35-14,742.73)	75.99 (51.03-108.75)	24,767.07 (16,437.35-35,026.54)	77.19 (51.69-108.5)	0.03 (0 to 0.06)
Vanuatu	89.25 (59.19-127.13)	99.5 (66.91-140.69)	228.26 (149.74-325.92)	99.7 (65.85-141.77)	-0.04 (-0.05 to -0.02)
Yemen	4,173.72 (2,809.82-6,011.92)	61.71 (41.87-88.7)	10,951.11 (7,434.42-15,685.88)	54.68 (37.51-78.3)	-0.41 (-0.45 to -0.38)
Zambia	5,838.88 (4,011.83-8,010.70)	146.05 (100.30-200.77)	16,310.07 (11,097.29-22,607.33)	148.06 (101.97-206.01)	0.03 (-0.06 to 0.11)
Zimbabwe	4,842.68 (3,222.36-6,764.32)	84.09 (56.22-115.97)	8,542.44 (5,748.85-12,012.17)	82.19 (55.80-115.56)	-0.01 (-0.04 to 0.02)

Table S3. The trends of SEV related to occupational noise-induced hearing loss among 204 countries and territories (1990–2021) .

Location	1990	2021	1990–2021
	**SEV, %, (95% UI)**	**SEV, %, (95% UI)**	**EAPC, %, (95% CI)**
Greenland	6.45 (5.86 - 7.03)	6.11 (5.75 - 6.61)	-0.16 (-0.19 to -0.13)
Sierra Leone	13.55 (12.68 - 14.55)	12.57 (11.97 - 13.32)	-0.21 (-0.26 to -0.16)
China	15.24 (14.69 - 15.92)	14.69 (14.15 - 15.38)	-0.13 (-0.17 to -0.09)
Togo	14.44 (13.52 - 15.58)	13.46 (12.79 - 14.26)	-0.20 (-0.24 to -0.17)
North Korea	10.34 (9.47 - 11.27)	11.11 (10.5 - 11.81)	0.24 (0.23 to 0.26)
Taiwan (Province of China)	10.45 (9.70 - 11.33)	10.65 (10.19 - 11.25)	0.06 (0.03 to 0.08)
Nauru	9.20 (8.44 - 10.18)	9.70 (9.14 - 10.37)	0.25 (0.22 to 0.27)
Cambodia	15.80 (14.97 - 16.68)	16.32 (15.7 - 17.04)	0.12 (0.11 to 0.13)
Maldives	11.19 (10.41 - 12.03)	11.05 (10.54 - 11.73)	-0.07 (-0.11 to -0.04)
Puerto Rico	3.30 (3.11 - 3.58)	3.60 (3.40 - 3.93)	0.24 (0.20 to 0.29)
Bermuda	10.5 (9.70 - 11.37)	11.20 (10.65 - 11.96)	0.22 (0.22 to 0.22)
Tuvalu	8.87 (8.09 - 9.82)	9.58 (9.11 - 10.19)	0.26 (0.26 to 0.27)
Lao People's Democratic Republic	17.24 (16.34 - 18.19)	16.23 (15.59 - 17.03)	-0.23 (-0.26 to -0.2)
Philippines	12.07 (11.48 - 12.85)	11.12 (10.64 - 11.87)	-0.26 (-0.27 to -0.26)
Northern Mariana Islands	8.93 (8.36 - 9.61)	10.51 (9.95 - 11.24)	0.58 (0.53 to 0.62)
Indonesia	12.74 (11.98 - 13.69)	12.17 (11.61 - 12.85)	-0.17 (-0.19 to -0.16)
Vietnam	14.54 (13.68 - 15.56)	15.16 (14.57 - 15.93)	0.16 (0.15 to 0.17)

San Marino	7.17 (6.68 - 7.86)	6.68 (6.33 - 7.28)	-0.25 (-0.26 to -0.23)
Cook Islands	10.90 (10.14 - 11.82)	11.11 (10.56 - 11.85)	0.04 (0.01 to 0.07)
South Sudan	14.26 (13.21 - 15.29)	14.05 (13.4 - 14.81)	-0.05 (-0.06 to -0.04)
Thailand	13.60 (13.04 - 14.35)	13.37 (12.84 - 14.1)	-0.05 (-0.06 to -0.04)
American Samoa	9.52 (8.81 - 10.36)	9.26 (8.73 - 9.94)	-0.09 (-0.11 to -0.07)
Micronesia (Federated States of)	8.95 (8.04 - 9.91)	9.27 (8.75 - 9.85)	0.13 (0.11 to 0.16)
Tonga	7.39 (6.88 - 8.04)	8.18 (7.64 - 8.78)	0.25 (0.17 to 0.32)
Armenia	7.49 (6.74 - 8.28)	8.36 (7.96 - 8.91)	0.42 (0.35 to 0.48)
Fiji	9.27 (8.51 - 10.19)	8.98 (8.50 - 9.56)	-0.14 (-0.15 to -0.12)
Kyrgyzstan	11.17 (10.25 - 12.10)	11.11 (10.62 - 11.74)	0.01 (-0.01 to 0.02)
Samoa	8.03 (7.28 - 8.85)	7.42 (7.00 - 7.99)	-0.26 (-0.34 to -0.18)
Uzbekistan	11.10 (10.26 - 12.05)	11.41 (10.91 - 12.03)	0.06 (0.03 to 0.1)
Sudan	8.44 (7.59 - 9.31)	8.57 (8.10 - 9.24)	0.05 (0.02 to 0.07)
Bulgaria	6.50 (6.04 - 7.10)	5.98 (5.68 - 6.45)	-0.25 (-0.26 to -0.25)
Myanmar	8.27 (7.79 - 8.85)	11.43 (10.97 - 12.04)	1.19 (1.14 to 1.24)
Macedonia	4.43 (3.97 - 4.99)	4.58 (4.34 - 4.95)	0.06 (0.01 to 0.10)
Guam	9.23 (8.73 - 9.89)	8.68 (8.22 - 9.36)	-0.19 (-0.2 to -0.18)
Malaysia	12.08 (11.6 - 12.78)	11.74 (11.24 - 12.42)	-0.11 (-0.13 to -0.10)
Georgia	9.91 (9.03 - 10.80)	9.85 (9.38 - 10.52)	-0.04 (-0.07 to -0.02)
Serbia	5.45 (4.94 - 6.00)	5.44 (5.15 - 5.89)	-0.04 (-0.06 to -0.03)
Tajikistan	7.93 (7.10 - 8.81)	7.82 (7.32 - 8.37)	-0.08 (-0.12 to -0.05)

Monaco	6.46 (5.92 - 7.06)	6.07 (5.70 - 6.62)	-0.20 (-0.23 to -0.17)
Sri Lanka	7.98 (7.42 - 8.64)	9.18 (8.79 - 9.67)	0.47 (0.42 to 0.52)
Palau	9.24 (8.61 - 10.04)	9.64 (9.19 - 10.29)	0.11 (0.08 to 0.14)
Belarus	7.15 (6.66 - 7.78)	7.17 (6.82 - 7.63)	-0.02 (-0.08 to 0.03)
Albania	6.46 (5.89 - 7.12)	6.38 (6.07 - 6.83)	-0.02 (-0.02 to -0.01)
Moldova	6.06 (5.53 - 6.67)	5.32 (5.03 - 5.77)	-0.47 (-0.56 to -0.38)
Tokelau	8.55 (7.77 - 9.46)	9.09 (8.44 - 9.93)	0.20 (0.19 to 0.22)
Czechia	8.05 (7.55 - 8.71)	7.18 (6.83 - 7.76)	-0.42 (-0.44 to -0.40)
Timor-Leste	10.52 (9.68 - 11.45)	10.08 (9.54 - 10.71)	-0.17 (-0.21 to -0.13)
Brunei	6.06 (5.66 - 6.52)	5.98 (5.70 - 6.40)	-0.04 (-0.05 to -0.03)
Niue	9.17 (8.39 - 10.10)	9.45 (8.98 - 10.08)	0.11 (0.09 to 0.13)
Papua New Guinea	8.38 (7.44 - 9.31)	8.29 (7.81 - 8.88)	-0.04 (-0.05 to -0.03)
Kiribati	3.37 (2.89 - 3.93)	4.84 (4.55 - 5.20)	1.23 (1.02 to 1.44)
Poland	6.76 (6.29 - 7.31)	6.24 (5.94 - 6.71)	-0.32 (-0.34 to -0.29)
Andorra	6.49 (6.01 - 7.08)	6.45 (6.05 - 7.02)	-0.03 (-0.04 to -0.02)
Slovenia	6.87 (6.39 - 7.41)	6.81 (6.48 - 7.28)	-0.01 (-0.03 to 0)
Latvia	6.57 (6.06 - 7.14)	6.41 (6.10 - 6.86)	-0.08 (-0.09 to -0.07)
Saint Kitts and Nevis	12.31 (11.77 - 13.02)	11.4 (10.81 - 12.18)	-0.29 (-0.31 to -0.27)
Denmark	7.30 (6.91 - 7.80)	7.30 (6.95 - 7.8)	0.01 (-0.01 to 0.02)
Greece	6.12 (5.79 - 6.57)	5.13 (4.87 - 5.55)	-0.55 (-0.60 to -0.49)
Vanuatu	12.93 (11.98 - 14.01)	13.15 (12.57 - 13.91)	0.07 (0.04 to 0.10)

Ukraine	7.07 (6.46 - 7.73)	6.8 (6.43 - 7.31)	-0.13 (-0.16 to -0.09)
Marshall Islands	7.00 (6.37 - 7.80)	6.62 (6.25 - 7.24)	-0.18 (-0.19 to -0.16)
Azerbaijan	12.91 (12.08 - 13.76)	11.97 (11.49 - 12.67)	-0.27 (-0.31 to -0.23)
Italy	5.76 (5.46 - 6.19)	5.33 (5.06 - 5.74)	-0.23 (-0.25 to -0.20)
Mongolia	10.00 (9.05 - 11.01)	10.20 (9.74 - 10.82)	0.11 (0.10 to 0.13)
Norway	7.44 (7.03 - 8.08)	7.21 (6.86 - 7.79)	-0.07 (-0.08 to -0.06)
Solomon Islands	6.77 (5.89 - 7.75)	10.71 (10.16 - 11.37)	1.71 (1.46 to 1.95)
Switzerland	7.44 (7.03 - 8.00)	7.52 (7.14 - 8.04)	0.03 (0.02 to 0.04)
Chile	7.72 (7.33 - 8.25)	9.55 (9.14 - 10.10)	0.67 (0.66 to 0.69)
Virgin Islands, U.S.	9.21 (8.40 - 10.04)	9.88 (9.19 - 10.67)	0.23 (0.21 to 0.25)
United States	6.73 (6.38 - 7.24)	6.40 (6.07 - 6.89)	-0.18 (-0.21 to -0.15)
The Bahamas	8.18 (7.51 - 8.83)	10.37 (9.88 - 10.96)	0.83 (0.79 to 0.87)
Dominican Republic	9.27 (8.58 - 10.01)	10.04 (9.58 - 10.68)	0.28 (0.27 to 0.30)
Jamaica	10.6 (10.12 - 11.24)	10.36 (9.87 - 11.00)	-0.11 (-0.12 to -0.09)
Trinidad and Tobago	9.88 (9.40 - 10.50)	10.52 (10.09 - 11.13)	0.26 (0.22 to 0.30)
Peru	12.14 (11.27 - 13.19)	12.83 (12.25 - 13.58)	0.26 (0.22 to 0.31)
El Salvador	8.91 (8.21 - 9.68)	9.84 (9.41 - 10.52)	0.39 (0.34 to 0.44)
Croatia	5.02 (4.54 - 5.63)	5.35 (5.09 - 5.78)	0.30 (0.26 to 0.35)
Nicaragua	9.20 (8.49 - 9.94)	11.00 (10.52 - 11.65)	0.63 (0.57 to 0.68)
Brazil	11.34 (10.51 - 12.29)	11.17 (10.65 - 11.87)	-0.01 (-0.03 to 0)
Algeria	6.77 (6.31 - 7.30)	6.45 (6.13 - 6.87)	-0.10 (-0.19 to -0.01)
Iraq	7.47 (6.80 - 8.12)	7.53 (7.16 - 7.97)	0.05 (0.05 to 0.06)

Libya	7.88 (7.21 - 8.66)	6.77 (6.38 - 7.28)	-0.48 (-0.48 to -0.47)
Montenegro	4.25 (3.78 - 4.80)	4.19 (3.94 - 4.57)	-0.11 (-0.15 to -0.07)
Saudi Arabia	8.30 (7.69 - 8.91)	8.09 (7.69 - 8.58)	-0.09 (-0.13 to -0.06)
United Arab Emirates	11.72 (11.09 - 12.34)	12.73 (12.15 - 13.47)	0.25 (0.22 to 0.28)
Afghanistan	9.59 (8.90 - 10.36)	9.46 (9.01 - 10.02)	-0.02 (-0.03 to 0)
Nepal	17.69 (16.76 - 18.66)	15.32 (14.74 - 16.12)	-0.34 (-0.46 to -0.23)
Angola	11.84 (10.91 - 12.82)	11.69 (11.05 - 12.49)	0 (-0.02 to 0.02)
Equatorial Guinea	10.76 (9.93 - 11.83)	11.17 (10.41 - 12.00)	0.14 (0.11 to 0.16)
Slovakia	6.99 (6.49 - 7.58)	6.34 (6.01 - 6.80)	-0.37 (-0.38 to -0.35)
Comoros	8.20 (7.50 - 8.96)	8.13 (7.72 - 8.79)	-0.02 (-0.02 to -0.02)
South Korea	6.53 (6.21 - 7.09)	5.87 (5.56 - 6.41)	-0.37 (-0.38 to -0.36)
Estonia	8.56 (8.03 - 9.22)	7.21 (6.85 - 7.80)	-0.60 (-0.62 to -0.58)
Kazakhstan	12.07 (11.33 - 12.94)	12.09 (11.59 - 12.7)	0.01 (0.01 to 0.02)
Russian Federation	7.57 (7.06 - 8.16)	7.06 (6.71 - 7.56)	-0.21 (-0.23 to -0.2)
Turkmenistan	7.77 (7.01 - 8.58)	8.34 (7.87 - 8.95)	0.24 (0.21 to 0.27)
Japan	6.58 (6.21 - 7.09)	6.66 (6.33 - 7.22)	0.02 (0 to 0.03)
Australia	6.72 (6.41 - 7.15)	6.90 (6.58 - 7.35)	0.11 (0.09 to 0.12)
Bosnia and Herzegovina	4.32 (3.84 - 4.97)	4.35 (4.07 - 4.67)	-0.03 (-0.06 to 0)
Austria	6.38 (6.06 - 6.78)	6.97 (6.64 - 7.39)	0.31 (0.30 to 0.32)
Hungary	6.68 (6.19 - 7.25)	6.06 (5.76 - 6.57)	-0.37 (-0.40 to -0.34)
Finland	7.70 (7.33 - 8.19)	6.89 (6.56 - 7.42)	-0.33 (-0.35 to -0.31)

Iceland	8.13 (7.59 - 8.81)	8.09 (7.69 - 8.76)	-0.03 (-0.05 to -0.02)
Luxembourg	5.99 (5.69 - 6.39)	5.45 (5.20 - 5.84)	-0.32 (-0.32 to -0.31)
Romania	8.48 (7.94 - 9.16)	7.42 (7.06 - 8.01)	-0.5 (-0.55 to -0.44)
Portugal	7.54 (7.17 - 8.05)	6.54 (6.22 - 7.05)	-0.47 (-0.49 to -0.45)
United Kingdom	6.78 (6.42 - 7.28)	6.86 (6.54 - 7.37)	0.03 (0.01 to 0.05)
Uruguay	9.08 (8.55 - 9.68)	10.6 (10.15 - 11.22)	0.56 (0.53 to 0.59)
Barbados	9.79 (9.29 - 10.43)	9.96 (9.46 - 10.62)	0.07 (0.05 to 0.09)
Belize	9.81 (9.17 - 10.57)	10.10 (9.65 - 10.71)	0.08 (0.05 to 0.12)
Grenada	9.02 (8.49 - 9.78)	10.10 (9.50 - 10.79)	0.38 (0.35 to 0.41)
Kenya	11.49 (10.63 - 12.52)	12.36 (11.83 - 13.05)	0.22 (0.16 to 0.28)
Saint Lucia	9.94 (9.10 - 10.90)	9.74 (9.21 - 10.38)	-0.07 (-0.07 to -0.06)
Mozambique	14.61 (13.48 - 15.84)	14.77 (14.15 - 15.59)	0.01 (0 to 0.03)
Lithuania	6.42 (5.93 - 7.00)	6.37 (6.05 - 6.84)	-0.06 (-0.07 to -0.04)
Tanzania	17.61 (16.71 - 18.66)	16.34 (15.67 - 17.15)	-0.25 (-0.29 to -0.21)
Guatemala	12.64 (11.82 - 13.58)	12.15 (11.59 - 12.82)	-0.13 (-0.15 to -0.11)
Singapore	5.70 (5.35 - 6.17)	5.84 (5.55 - 6.28)	0.12 (0.08 to 0.16)
Panama	9.80 (9.38 - 10.42)	10.48 (10.05 - 11.08)	0.23 (0.19 to 0.27)
Paraguay	10.52 (9.70 - 11.42)	11.63 (11.06 - 12.34)	0.38 (0.36 to 0.40)
Bahrain	9.45 (8.88 - 10.13)	10.16 (9.71 - 10.71)	0.28 (0.20 to 0.36)
Jordan	6.43 (5.85 - 7.07)	6.38 (6.08 - 6.79)	-0.02 (-0.03 to 0)
Morocco	8.32 (7.58 - 9.18)	8.34 (7.94 - 8.90)	0.07 (0.05 to 0.1)
Cyprus	5.83 (5.45 - 6.31)	5.78 (5.49 - 6.20)	0 (-0.04 to 0.03)

Germany	6.92 (6.57 - 7.40)	6.87 (6.55 - 7.35)	-0.05 (-0.06 to -0.03)
Lesotho	10.14 (9.17 - 11.14)	10.59 (10.06 - 11.22)	0.16 (0.15 to 0.17)
Israel	5.03 (4.74 - 5.48)	5.83 (5.56 - 6.26)	0.53 (0.49 to 0.57)
New Zealand	7.42 (7.02 - 7.95)	7.16 (6.82 - 7.68)	-0.10 (-0.12 to -0.09)
Zimbabwe	14.47 (13.72 - 15.40)	14.79 (14.22 - 15.58)	0.09 (0.07 to 0.11)
Netherlands	5.11 (4.85 - 5.46)	6.70 (6.38 - 7.11)	0.93 (0.90 to 0.96)
Sweden	8.12 (7.70 - 8.69)	7.14 (6.79 - 7.73)	-0.40 (-0.42 to -0.38)
Cameroon	13.26 (12.22 - 14.33)	13.81 (13.20 - 14.53)	0.19 (0.16 to 0.22)
The Gambia	10.98 (10.02 - 11.98)	10.30 (9.73 - 11.02)	-0.21 (-0.22 to -0.21)
Argentina	9.17 (8.37 - 10.04)	9.62 (9.11 - 10.31)	0.21 (0.16 to 0.26)
Liberia	13.12 (12.07 - 14.22)	12.82 (12.20 - 13.61)	-0.09 (-0.10 to -0.08)
Canada	6.91 (6.56 - 7.35)	6.76 (6.41 - 7.24)	-0.06 (-0.07 to -0.04)
Nigeria	11.06 (10.23 - 11.97)	11.82 (11.26 - 12.57)	0.22 (0.21 to 0.23)
Antigua and Barbuda	10.15 (9.35 - 11.01)	10.59 (10.05 - 11.30)	0.14 (0.14 to 0.14)
Dominica	8.82 (8.16 - 9.57)	9.29 (8.76 - 9.94)	0.20 (0.19 to 0.21)
Haiti	8.00 (7.24 - 8.82)	8.95 (8.44 - 9.60)	0.37 (0.28 to 0.45)
Suriname	8.16 (7.48 - 8.96)	8.71 (8.30 - 9.31)	0.26 (0.21 to 0.32)
Ecuador	9.73 (8.92 - 10.66)	11.21 (10.71 - 11.91)	0.56 (0.51 to 0.61)
Syria	9.68 (8.93 - 10.48)	8.21 (7.81 - 8.84)	-0.56 (-0.67 to -0.45)
Belgium	5.50 (5.20 - 5.90)	5.63 (5.37 - 6.01)	0.11 (0.10 to 0.12)
France	5.95 (5.65 - 6.35)	5.91 (5.64 - 6.37)	0 (0 to 0.01)

Yemen	7.48 (6.93 - 8.10)	6.88 (6.56 - 7.29)	-0.27 (-0.30 to -0.24)
Bangladesh	9.86 (9.22 - 10.65)	10.70 (10.26 - 11.26)	0.28 (0.27 to 0.29)
Pakistan	7.97 (7.50 - 8.55)	9.39 (8.96 - 9.93)	0.57 (0.55 to 0.60)
Central African Republic	11.19 (10.30 - 12.20)	11.40 (10.68 - 12.30)	0.08 (0.07 to 0.08)
Ireland	5.87 (5.54 - 6.23)	5.98 (5.70 - 6.39)	0.10 (0.08 to 0.13)
Gabon	8.46 (7.77 - 9.21)	7.83 (7.35 - 8.43)	-0.26 (-0.30 to -0.21)
Malta	6.03 (5.53 - 6.50)	5.99 (5.70 - 6.39)	-0.06 (-0.07 to -0.04)
Spain	5.63 (5.34 - 6.07)	5.25 (4.99 - 5.63)	-0.16 (-0.20 to -0.12)
Costa Rica	10.91 (10.35 - 11.60)	10.46 (10.02 - 11.04)	-0.13 (-0.15 to -0.11)
Mexico	11.24 (10.57 - 12.03)	11.01 (10.52 - 11.62)	-0.07 (-0.07 to -0.07)
Iran	7.16 (6.53 - 7.80)	7.33 (6.98 - 7.80)	0.10 (0.08 to 0.12)
Djibouti	8.62 (7.83 - 9.43)	8.39 (7.91 - 8.95)	-0.14 (-0.16 to -0.12)
Madagascar	19.44 (18.67 - 20.28)	19.10 (18.48 - 19.86)	-0.05 (-0.06 to -0.04)
Rwanda	16.47 (15.57 - 17.43)	14.98 (14.41 - 15.67)	-0.24 (-0.31 to -0.17)
Uganda	15.65 (14.74 - 16.69)	14.87 (14.29 - 15.69)	-0.18 (-0.19 to -0.17)
Namibia	8.39 (7.59 - 9.22)	8.42 (8.00 - 9.04)	0 (-0.04 to 0.04)
Cape Verde	9.32 (8.56 - 10.14)	9.26 (8.77 - 9.94)	0.01 (-0.01 to 0.03)
Cuba	9.76 (8.90 - 10.81)	10.34 (9.85 - 10.97)	0.17 (0.12 to 0.22)
Lebanon	7.85 (7.16 - 8.58)	7.96 (7.53 - 8.47)	0.03 (0.01 to 0.06)
Ghana	14.24 (13.31 - 15.35)	13.16 (12.53 - 13.95)	-0.24 (-0.28 to -0.19)
Mali	13.35 (12.39 - 14.54)	13.22 (12.64 - 13.94)	-0.01 (-0.03 to 0)
Sao Tome and Principe	8.79 (8.10 - 9.55)	7.29 (6.85 - 7.79)	-0.73 (-0.79 to -0.67)

Guyana	9.42 (8.65 - 10.24)	9.52 (9.10 - 10.15)	0.03 (0.02 to 0.03)
Qatar	13.68 (12.93 - 14.55)	14.24 (13.69 - 14.9)	0.27 (0.22 to 0.32)
Turkey	10.94 (10.36 - 11.70)	9.44 (9.04 - 9.99)	-0.56 (-0.59 to -0.53)
Saint Vincent and the Grenadines	9.06 (8.28 - 9.97)	9.30 (8.82 - 9.97)	0.08 (0.07 to 0.09)
India	11.21 (10.45 - 12.01)	10.49 (10.05 - 11.08)	-0.20 (-0.25 to -0.16)
Democratic Republic of the Congo	13.37 (12.43 - 14.46)	13.19 (12.6 - 13.94)	-0.02 (-0.05 to 0.01)
Burundi	16.71 (15.77 - 17.66)	16.86 (16.19 - 17.62)	0.03 (0.02 to 0.03)
Ethiopia	14.65 (13.74 - 15.62)	14.19 (13.64 - 14.88)	-0.15 (-0.17 to -0.13)
Mauritius	9.77 (9.02 - 10.59)	10.13 (9.66 - 10.71)	0.12 (0.11 to 0.13)
Somalia	13.53 (12.55 - 14.51)	13.91 (13.07 - 14.8)	0.11 (0.09 to 0.12)
Botswana	8.28 (7.53 - 9.13)	9.26 (8.83 - 9.87)	0.42 (0.36 to 0.48)
Swaziland	8.11 (7.31 - 8.98)	7.71 (7.24 - 8.30)	-0.17 (-0.17 to -0.17)
Burkina Faso	16.24 (15.35 - 17.19)	14.73 (14.18 - 15.43)	-0.30 (-0.36 to -0.24)
Cote d'Ivoire	12.52 (11.62 - 13.52)	12.06 (11.46 - 12.78)	-0.09 (-0.11 to -0.07)
Guinea-Bissau	11.66 (10.63 - 12.71)	11.98 (11.18 - 12.83)	0.1 (0.08 to 0.13)
Niger	14.24 (13.29 - 15.24)	13.44 (12.78 - 14.28)	-0.22 (-0.24 to -0.20)
Bolivia	8.55 (8.05 - 9.12)	12.17 (11.70 - 12.84)	1.22 (1.16 to 1.27)
Colombia	10.41 (9.55 - 11.34)	10.84 (10.30 - 11.50)	0.13 (0.09 to 0.16)
Honduras	7.14 (6.79 - 7.63)	10.81 (10.38 - 11.39)	1.35 (1.28 to 1.41)
Venezuela	10.5 (10.02 - 11.15)	10.25 (9.78 - 10.89)	-0.02 (-0.04 to 0)
Egypt	8.12 (7.54 - 8.85)	8.43 (8.03 - 8.99)	0.16 (0.14 to 0.17)

Kuwait	10.09 (9.58 - 10.74)	10.65 (10.17 - 11.31)	0.19 (0.18 to 0.20)
Palestine	6.21 (5.64 - 6.87)	6.33 (6.02 - 6.72)	0.01 (-0.01 to 0.04)
Oman	7.38 (6.71 - 8.09)	8.25 (7.87 - 8.73)	0.42 (0.26 to 0.58)
Tunisia	8.46 (7.93 - 9.03)	7.92 (7.57 - 8.41)	-0.21 (-0.21 to -0.20)
Bhutan	12.47 (11.53 - 13.35)	12.55 (12.01 - 13.22)	0.02 (0.01 to 0.03)
Congo	9.51 (8.66 - 10.44)	9.25 (8.69 - 9.99)	-0.08 (-0.11 to -0.05)
Eritrea	14.11 (13.13 - 15.20)	14.28 (13.48 - 15.09)	0.05 (0.03 to 0.07)
Malawi	14.63 (13.65 - 15.76)	15.3 (14.57 - 16.14)	0.21 (0.18 to 0.24)
Seychelles	11.72 (10.95 - 12.63)	11.73 (11.19 - 12.46)	0.01 (-0.01 to 0.03)
Zambia	14.18 (13.19 - 15.24)	14.06 (13.48 - 14.84)	-0.03 (-0.04 to -0.02)
South Africa	7.51 (6.74 - 8.33)	7.28 (6.89 - 7.83)	-0.10 (-0.11 to -0.09)
Benin	15.10 (14.25 - 16.18)	14.42 (13.78 - 15.27)	-0.15 (-0.16 to -0.13)
Chad	11.51 (10.65 - 12.46)	12.35 (11.62 - 13.23)	0.25 (0.23 to 0.28)
Guinea	13.29 (12.25 - 14.28)	13.11 (12.48 - 13.9)	-0.03 (-0.05 to -0.02)
Mauritania	8.61 (8.05 - 9.26)	8.06 (7.64 - 8.65)	-0.22 (-0.24 to -0.20)
Senegal	11.30 (10.40 - 12.39)	10.19 (9.70 - 10.80)	-0.33 (-0.38 to -0.28)

Table S4. Changes in DALYs of ONIHL according to decomposition analysis from 1990 to 2021 at the global, SDI, and 21 GBD regional levels.

Location	Overall	Aging	Population growth	Epidemiological changes
**Global**	4,009,389.28	839,545.3 (20.94%)	2,735,470.93 (68.23%)	434,373.05 (10.83%)
**SDI regions**				
High	289,206.21	94,843.77 (32.79%)	163,514.78 (56.54%)	30,847.66 (10.67%)
High-middle	802,812.27	251,212.92 (31.29%)	367,438.13 (45.77%)	184,161.21 (22.94%)
Middle	1,589,063.83	558,698.86 (35.16%)	1,044,341.67 (65.72%)	-13,976.7 (-0.88%)
Low-middle	880,224.21	119,520.04 (13.58%)	763,705.13 (86.76%)	-3,000.96 (-0.34%)
Low	446,178.59	-15,831.1 (-3.55%)	475,739 (106.63%)	-13,729.31 (-3.08%)
**GBD regions**				
East Asia	1,426,093.71	666,690 (46.75%)	589,166.59 (41.31%)	170,237.11 (11.94%)
Southeast Asia	485,327.99	129,069.01 (26.59%)	355,500.87 (73.25%)	758.12 (0.16%)
Oceania	4,468.47	372.95 (8.35%)	4,029.17 (90.17%)	66.35 (1.48%)
Central Asia	30,375.32	7,193.29 (23.68%)	23,158.72 (76.24%)	23.31 (0.08%)
Central Europe	11,755.7	13,539.75 (115.18%)	1,643.42 (13.98%)	-3,427.47 (-29.16%)
Eastern Europe	11,571	20,739.51 (179.24%)	-3,466.12 (-29.96%)	-5,702.39 (-49.28%)
High-income Asia Pacific	38,521.98	24,300.42 (63.08%)	15,219.68 (39.51%)	-998.13 (-2.59%)
Australasia	8,447.9	2,194.16 (25.97%)	6,166.74 (73%)	87 (1.03%)
Western Europe	52,306.6	22,991.06 (43.95%)	27,419.94 (52.42%)	1,895.61 (3.62%)
Southern Latin America	19,018.96	3,501.24 (18.41%)	13,902.99 (73.1%)	1,614.73 (8.49%)

High-income North America	88,691.79	38,596.1 (43.52%)	66,683.87 (75.19%)	-16,588.18 (-18.7%)
Caribbean	15,014.68	4,420.32 (29.44%)	9,490.86 (63.21%)	1,103.49 (7.35%)
Andean Latin America	27,120.87	4,934.98 (18.2%)	19,634 (72.39%)	2,551.9 (9.41%)
Central Latin America	106,204.02	29,782.42 (28.04%)	75,545.09 (71.13%)	876.51 (0.83%)
Tropical Latin America	124,059.65	36,884.83 (29.73%)	88,874.13 (71.64%)	-1,699.31 (-1.37%)
North Africa and Middle East	206,496.88	36,798.61 (17.82%)	189,616.38 (91.83%)	-19,918.1 (-9.65%)
South Asia	879,854.88	130,326.23 (14.81%)	801,717.33 (91.12%)	-52,188.68 (-5.93%)
Central Sub-Saharan Africa	51,230.61	-882.13 (-1.72%)	54,152.84 (105.7%)	-2,040.1 (-3.98%)
Eastern Sub-Saharan Africa	233,523.49	-5,748.19 (-2.46%)	231,460.14 (99.12%)	7,811.54 (3.35%)
Southern Sub-Saharan Africa	21,855.03	4,693.03 (21.47%)	18,039.83 (82.54%)	-877.82 (-4.02%)
Western Sub-Saharan Africa	167,449.75	-9,631.36 (-5.75)	179,911.47 (107.44%)	-2,830.36 (-1.69%)

Table S5. The effective difference in ASDR among countries and territories in 2021 for ONIHL according to frontier analysis.

**Location**	**ASDR, per 100,000**	**SDI level**	**Efficiency difference**
Madagascar	216.77	0.4002	168.77
Kenya	162.52	0.5238	131.00
Rwanda	156.52	0.4356	124.81
Lao People's Democratic Republic	149.21	0.4891	117.68
Zambia	148.06	0.5059	116.55
Cambodia	147.65	0.4736	116.13
Malawi	162.01	0.3846	114.08
China	133.99	0.7216	109.66
Viet Nam	139.77	0.6279	108.26
United Republic of Tanzania	139.30	0.4466	107.74
Burundi	169.25	0.2894	107.62
Ethiopia	154.62	0.3588	106.76
Thailand	127.45	0.6825	101.46
Eritrea	148.51	0.4039	100.55
Mozambique	152.85	0.3265	97.65
Nepal	126.50	0.4332	94.75
Seychelles	114.96	0.7302	91.23

Malaysia	114.04	0.7425	90.58
Uganda	121.98	0.4233	89.62
Myanmar	119.87	0.5339	88.35
Indonesia	113.43	0.6569	86.64
Philippines	114.89	0.6512	86.61
South Sudan	147.90	0.2784	86.15
Maldives	111.44	0.6509	83.48
Bhutan	108.02	0.4731	76.51
Mauritius	100.85	0.7183	76.31
Timor-Leste	105.16	0.4447	73.61
Qatar	95.54	0.8469	72.94
Djibouti	103.75	0.4880	72.23
Equatorial Guinea	96.35	0.6579	69.82
Angola	100.74	0.4537	69.22
Sri Lanka	94.32	0.7015	68.33
Vanuatu	99.70	0.4731	68.18
Comoros	99.45	0.4760	67.94
Nigeria	99.38	0.5034	67.86
United Arab Emirates	90.50	0.8493	67.84
Cameroon	99.09	0.4797	67.57
India	96.37	0.5754	64.86

Cook Islands	85.28	0.7791	62.01
Benin	109.34	0.3735	61.38
Cote d'Ivoire	93.34	0.4259	60.91
Ghana	92.28	0.5649	60.76
Democratic Republic of the Congo	108.55	0.3832	60.64
Northern Mariana Islands	82.64	0.7715	59.34
Brazil	85.62	0.6530	58.37
Bangladesh	89.55	0.4924	58.03
Kazakhstan	80.10	0.7251	56.14
Kuwait	78.18	0.8467	55.51
Azerbaijan	81.15	0.6949	55.13
Paraguay	85.20	0.6357	53.69
Palau	76.76	0.7540	53.43
Congo	84.50	0.5831	52.98
Bahrain	75.36	0.7530	52.08
Niue	76.04	0.7262	51.96
Uzbekistan	77.19	0.6626	51.11
Togo	98.77	0.4085	50.90
American Samoa	74.92	0.7237	50.74
Zimbabwe	82.19	0.4738	50.67
Solomon Islands	82.15	0.4294	50.23

Bermuda	71.76	0.8214	49.12
Guam	71.85	0.8040	49.09
Sierra Leone	96.89	0.3587	48.63
Saint Kitts and Nevis	71.91	0.755	48.56
Tokelau	73.75	0.6864	47.74
Guinea	98.74	0.3364	47.59
Ecuador	73.53	0.6610	47.26
Liberia	94.90	0.3524	46.81
Georgia	70.29	0.7325	46.75
Turkey	71.72	0.7127	46.62
Pakistan	78.08	0.5040	46.58
Fiji	72.53	0.6751	46.53
Burkina Faso	107.62	0.2851	45.90
Nauru	76.96	0.6252	45.45
Democratic People's Republic of Korea	76.40	0.5699	44.88
Cabo Verde	76.35	0.5335	44.84
Tuvalu	76.31	0.5766	44.79
Mexico	70.80	0.6646	44.68
Antigua and Barbuda	67.98	0.7499	44.61
Kyrgyzstan	75.48	0.6040	43.97
Micronesia (Federated States of)	74.88	0.5875	43.36

Guinea-Bissau	91.38	0.3531	43.28
Trinidad and Tobago	66.23	0.7688	42.91
Gabon	74.11	0.6347	42.59
Bahamas	65.05	0.8050	42.17
Jamaica	67.46	0.6833	41.44
United States Virgin Islands	64.16	0.8218	41.41
Barbados	64.30	0.7467	40.95
Cuba	66.30	0.6687	40.21
Guatemala	71.35	0.5400	39.82
Saudi Arabia	62.36	0.8151	39.70
Mongolia	70.82	0.6176	39.30
Colombia	66.50	0.6554	39.15
Grenada	65.11	0.6690	39.08
Taiwan (Province of China)	61.35	0.8747	38.70
Panama	64.40	0.7089	38.39
Saint Lucia	63.98	0.6725	37.96
Oman	61.23	0.7734	37.93
Costa Rica	63.87	0.7003	37.87
Dominica	61.03	0.7470	37.69
Sudan	69.16	0.5419	37.64
Mali	99.31	0.2686	37.54

Lebanon	60.88	0.7447	37.53
Peru	63.50	0.6621	37.39
Central African Republic	97.50	0.3092	36.95
Mauritania	68.40	0.4989	36.89
Bolivia (Plurinational State of)	68.30	0.5990	36.79
Tonga	68.03	0.6263	36.52
Papua New Guinea	70.44	0.4178	36.39
Tunisia	62.12	0.6824	36.11
Lesotho	66.75	0.5104	35.24
Armenia	61.16	0.7018	35.16
Turkmenistan	61.00	0.6822	35.00
Morocco	65.97	0.5627	34.46
Nicaragua	65.59	0.5240	34.06
Iran (Islamic Republic of)	59.86	0.6972	33.86
Gambia	81.71	0.4097	33.72
Chad	95.57	0.2404	33.62
Egypt	65.14	0.6068	33.61
Dominican Republic	65.01	0.6194	33.49
Belize	64.78	0.6102	33.27
Senegal	80.93	0.4081	33.03
Russian Federation	55.71	0.8085	32.96

Guyana	60.73	0.6508	32.94
Honduras	64.37	0.5130	32.85
Estonia	55.16	0.8449	32.56
Venezuela (Bolivarian Republic of)	63.99	0.5965	32.48
Romania	55.31	0.7685	31.98
Uruguay	56.21	0.7193	31.74
Sao Tome and Principe	63.17	0.5054	31.64
Samoa	63.05	0.5934	31.53
Syrian Arab Republic	62.95	0.6230	31.44
Saint Vincent and the Grenadines	61.45	0.6372	31.30
Libya	55.37	0.7258	31.25
Iraq	57.15	0.6626	31.13
Botswana	61.059	0.6427	31.10
Ukraine	54.16	0.7608	30.84
South Africa	56.85	0.6796	30.83
Czechia	53.28	0.8285	30.58
Belarus	53.71	0.7845	30.42
Chile	53.56	0.7715	30.24
Poland	52.01	0.8120	29.14
El Salvador	60.60	0.5638	29.09
Argentina	52.64	0.7231	28.64

Haiti	59.57	0.4483	28.01
Tajikistan	58.85	0.5415	27.34
Latvia	49.72	0.8307	26.95
Jordan	50.52	0.7253	26.59
Lithuania	49.19	0.8565	26.49
Suriname	57.49	0.6337	25.98
Marshall Islands	57.37	0.5741	25.85
Namibia	57.26	0.6176	25.75
Algeria	51.67	0.6595	25.52
Slovakia	48.28	0.8106	25.49
United States of America	47.18	0.8624	24.59
Slovenia	46.64	0.8424	24.00
Albania	49.27	0.7068	23.28
Yemen	54.68	0.4504	23.16
Hungary	46.35	0.7908	23.07
Bulgaria	46.24	0.7682	23.00
Greenland	45.04	0.8262	22.36
Canada	44.08	0.8732	21.45
Eswatini	52.77	0.5855	21.25
New Zealand	43.62	0.8494	21.05
Palestine	51.46	0.6310	19.94

Republic of Moldova	43.41	0.7322	19.71
Serbia	42.28	0.7924	18.96
Afghanistan	67.74	0.3372	18.69
Somalia	144.70	0.0777	18.03
Croatia	41.19	0.7983	17.97
Australia	40.60	0.8443	17.90
Japan	37.44	0.8712	14.78
North Macedonia	36.53	0.7506	13.17
Norway	34.53	0.9161	11.92
United Kingdom	34.23	0.8590	11.60
Iceland	33.57	0.8764	10.98
Kiribati	42.47	0.5272	10.94
Bosnia and Herzegovina	34.68	0.7231	10.81
Montenegro	33.68	0.7958	10.53
Republic of Korea	32.56	0.8867	9.94
Brunei Darussalam	32.74	0.8102	9.91
Singapore	32.28	0.8561	9.64
Finland	29.42	0.8598	6.79
Germany	29.19	0.9030	6.60
Italy	29.14	0.8058	6.21
Switzerland	28.83	0.9331	6.18

San Marino	28.65	0.8880	5.97
Niger	99.97	0.1681	5.56
Austria	28.04	0.8538	5.46
Portugal	28.69	0.7442	5.42
France	28.10	0.8384	5.36
Andorra	27.90	0.8694	5.31
Puerto Rico	27.78	0.8255	5.09
Denmark	27.52	0.8964	4.88
Sweden	27.09	0.8869	4.45
Netherlands	26.92	0.8885	4.20
Monaco	26.83	0.9083	4.18
Ireland	26.12	0.8738	3.53
Belgium	25.92	0.8537	3.16
Israel	25.82	0.8090	3.02
Cyprus	25.47	0.8356	2.79
Malta	25.99	0.8016	2.79
Spain	25.98	0.7693	2.70
Greece	25.92	0.7919	2.60
Luxembourg	22.87	0.8844	0.34

Table S6. The future number of DALYs and ASDR of ONIHL globally from 2022 to 2040 by ARIMA model.

Sex	2022		2040	
	**Number of DALYs, (95% UI)**	**ASDR per 100 000 (95% UI)**	**Number of DALYs, (95% UI)**	**ASDR per 100 000 (95% UI)**
**Global**				
**Both**	7,969,135.74 (7,960,571.70–7,977,699.79)	91.05 (90.96–91.14)	9,778,997.44 (8,607,164.25–10,950,830.62)	88.66 (78.53–98.79)
**males**	4,835,323.70 (4,828,859.51–4,841,787.90)	112.96 (112.82–113.09)	5,113,855.40 (3,948,769.84–6,278,940.97)	100.52 (82.29–118.75)
**females**	3,131,992.22 (3,128,130.23–3,135,854.22)	70.16 (70.08–70.23)	4,301,293.44 (3,950,073.01–4,652,513.88)	75.35 (74.15–76.56)

Table S7 Country/Region Groupings Used in This Study to Including SDI Countries and GBD Regions

Location ID	Location Name	2021 SDI Index Value	SDI Quintile
1	Global	0.666367819	Middle SDI
4	Southeast Asia, East Asia, and Oceania	0.696630914	Middle SDI
5	East Asia	0.722912119	High-middle SDI
6	China	0.71867919	High-middle SDI
7	Democratic People's Republic of Korea	0.56945513	Low-middle SDI
8	Taiwan (Province of China)	0.875139514	High SDI
9	Southeast Asia	0.64907177	Middle SDI
10	Cambodia	0.473999694	Low-middle SDI
11	Indonesia	0.657934796	Middle SDI
12	Lao People's Democratic Republic	0.489280726	Low-middle SDI
13	Malaysia	0.742552841	High-middle SDI
14	Maldives	0.657665453	Middle SDI
15	Myanmar	0.528492169	Low-middle SDI
16	Philippines	0.651920253	Middle SDI

Location ID	Location Name	2021 SDI Index Value	SDI Quintile
17	Sri Lanka	0.701371778	Middle SDI
18	Thailand	0.682657272	Middle SDI
19	Timor-Leste	0.450689053	Low SDI
20	Viet Nam	0.621620778	Middle SDI
21	Oceania	0.467359461	Low-middle SDI
22	Fiji	0.669068631	Middle SDI
23	Kiribati	0.525957502	Low-middle SDI
24	Marshall Islands	0.573524783	Low-middle SDI
25	Micronesia (Federated States of)	0.588012508	Low-middle SDI
26	Papua New Guinea	0.418098053	Low SDI
27	Samoa	0.592340278	Low-middle SDI
28	Solomon Islands	0.429541799	Low SDI
29	Tonga	0.629100964	Middle SDI
30	Vanuatu	0.472796337	Low-middle SDI
31	Central Europe, Eastern Europe, and Central Asia	0.768649142	High-middle SDI
32	Central Asia	0.674963478	Middle SDI

Location ID	Location Name	2021 SDI Index Value	SDI Quintile
33	Armenia	0.702496602	Middle SDI
34	Azerbaijan	0.695410598	Middle SDI
35	Georgia	0.733123642	High-middle SDI
36	Kazakhstan	0.718331647	High-middle SDI
37	Kyrgyzstan	0.609180728	Low-middle SDI
38	Mongolia	0.618744133	Low-middle SDI
39	Tajikistan	0.536613238	Low-middle SDI
40	Turkmenistan	0.683039569	Middle SDI
41	Uzbekistan	0.664964654	Middle SDI
42	Central Europe	0.795780357	High-middle SDI
43	Albania	0.706888685	Middle SDI
44	Bosnia and Herzegovina	0.72296408	High-middle SDI
45	Bulgaria	0.764641037	High-middle SDI
46	Croatia	0.799069214	High-middle SDI
47	Czechia	0.828510085	High SDI
48	Hungary	0.791024669	High-middle SDI

Location ID	Location Name	2021 SDI Index Value	SDI Quintile
49	North Macedonia	0.750954677	High-middle SDI
50	Montenegro	0.796532951	High-middle SDI
51	Poland	0.812073312	High SDI
52	Romania	0.766321392	High-middle SDI
53	Serbia	0.79221264	High-middle SDI
54	Slovakia	0.808329132	High-middle SDI
55	Slovenia	0.842633141	High SDI
56	Eastern Europe	0.803414319	High-middle SDI
57	Belarus	0.784114127	High-middle SDI
58	Estonia	0.845787294	High SDI
59	Latvia	0.830715451	High SDI
60	Lithuania	0.857613278	High SDI
61	Republic of Moldova	0.732393345	High-middle SDI
62	Russian Federation	0.809111108	High-middle SDI
63	Ukraine	0.761045561	High-middle SDI
64	High-income	0.85286503	High SDI

Location ID	Location Name	2021 SDI Index Value	SDI Quintile
65	High-income Asia Pacific	0.877157409	High SDI
66	Brunei Darussalam	0.810288851	High SDI
67	Japan	0.871459701	High SDI
68	Republic of Korea	0.887195638	High SDI
69	Singapore	0.856235308	High SDI
70	Australasia	0.845644432	High SDI
71	Australia	0.844269408	High SDI
72	New Zealand	0.850145187	High SDI
73	Western Europe	0.848728514	High SDI
74	Andorra	0.869895393	High SDI
75	Austria	0.854558286	High SDI
76	Belgium	0.853674059	High SDI
77	Cyprus	0.835648571	High SDI
78	Denmark	0.897314038	High SDI
79	Finland	0.860244219	High SDI
80	France	0.837816091	High SDI

Location ID	Location Name	2021 SDI Index Value	SDI Quintile
81	Germany	0.903515704	High SDI
82	Greece	0.791882294	High-middle SDI
83	Iceland	0.874628639	High SDI
84	Ireland	0.873989853	High SDI
85	Israel	0.809091066	High-middle SDI
86	Italy	0.805537426	High-middle SDI
87	Luxembourg	0.884636327	High SDI
88	Malta	0.801853922	High-middle SDI
89	Netherlands	0.888375951	High SDI
90	Norway	0.916631633	High SDI
91	Portugal	0.745394909	High-middle SDI
92	Spain	0.76948336	High-middle SDI
93	Sweden	0.887384361	High SDI
94	Switzerland	0.933531726	High SDI
95	United Kingdom	0.858444983	High SDI
96	Southern Latin America	0.743029817	High-middle SDI

Location ID	Location Name	2021 SDI Index Value	SDI Quintile
97	Argentina	0.733528396	High-middle SDI
98	Chile	0.770149297	High-middle SDI
99	Uruguay	0.721713499	High-middle SDI
100	High-income North America	0.86421664	High SDI
101	Canada	0.873181934	High SDI
102	United States of America	0.863243823	High SDI
103	Latin America and Caribbean	0.646195591	Middle SDI
104	Caribbean	0.6423146	Middle SDI
105	Antigua and Barbuda	0.749849952	High-middle SDI
106	Bahamas	0.805143711	High-middle SDI
107	Barbados	0.74706542	High-middle SDI
108	Belize	0.61055234	Low-middle SDI
109	Cuba	0.669331767	Middle SDI
110	Dominica	0.747381853	High-middle SDI
111	Dominican Republic	0.619170694	Middle SDI
112	Grenada	0.6693506	Middle SDI

Location ID	Location Name	2021 SDI Index Value	SDI Quintile
113	Guyana	0.650902479	Middle SDI
114	Haiti	0.448751017	Low SDI
115	Jamaica	0.68306364	Middle SDI
116	Saint Lucia	0.672601687	Middle SDI
117	Saint Vincent and the Grenadines	0.640886762	Middle SDI
118	Suriname	0.641162711	Middle SDI
119	Trinidad and Tobago	0.769401094	High-middle SDI
120	Andean Latin America	0.654007956	Middle SDI
121	Bolivia (Plurinational State of)	0.604496662	Low-middle SDI
122	Ecuador	0.665675436	Middle SDI
123	Peru	0.662036006	Middle SDI
124	Central Latin America	0.641931122	Middle SDI
125	Colombia	0.65664043	Middle SDI
126	Costa Rica	0.704369665	Middle SDI
127	El Salvador	0.565569678	Low-middle SDI
128	Guatemala	0.540099007	Low-middle SDI

Location ID	Location Name	2021 SDI Index Value	SDI Quintile
129	Honduras	0.513585699	Low-middle SDI
130	Mexico	0.66496867	Middle SDI
131	Nicaragua	0.52364671	Low-middle SDI
132	Panama	0.706659844	Middle SDI
133	Venezuela (Bolivarian Republic of)	0.596599587	Low-middle SDI
134	Tropical Latin America	0.648941531	Middle SDI
135	Brazil	0.648846512	Middle SDI
136	Paraguay	0.650487525	Middle SDI
137	North Africa and Middle East	0.658716072	Middle SDI
138	North Africa and Middle East	0.658716072	Middle SDI
139	Algeria	0.659720087	Middle SDI
140	Bahrain	0.752218099	High-middle SDI
141	Egypt	0.603962121	Low-middle SDI
142	Iran (Islamic Republic of)	0.69729326	Middle SDI
143	Iraq	0.662777495	Middle SDI
144	Jordan	0.725420238	High-middle SDI

Location ID	Location Name	2021 SDI Index Value	SDI Quintile
145	Kuwait	0.846802486	High SDI
146	Lebanon	0.741226017	High-middle SDI
147	Libya	0.73508433	High-middle SDI
148	Morocco	0.561680434	Low-middle SDI
149	Palestine	0.629201641	Middle SDI
150	Oman	0.773801229	High-middle SDI
151	Qatar	0.846704498	High SDI
152	Saudi Arabia	0.814515567	High SDI
153	Syrian Arab Republic	0.622855859	Middle SDI
154	Tunisia	0.681701488	Middle SDI
155	Turkey	0.713246106	High-middle SDI
156	United Arab Emirates	0.849740335	High SDI
157	Yemen	0.453539967	Low SDI
158	South Asia	0.559642669	Low-middle SDI
159	South Asia	0.559642669	Low-middle SDI
160	Afghanistan	0.335068107	Low SDI

Location ID	Location Name	2021 SDI Index Value	SDI Quintile
161	Bangladesh	0.493106236	Low-middle SDI
162	Bhutan	0.476724988	Low-middle SDI
163	India	0.5777383	Low-middle SDI
164	Nepal	0.433952916	Low SDI
165	Pakistan	0.504275856	Low-middle SDI
166	Sub-Saharan Africa	0.461224007	Low SDI
167	Central Sub-Saharan Africa	0.484517732	Low-middle SDI
168	Angola	0.482946052	Low-middle SDI
169	Central African Republic	0.311026626	Low SDI
170	Congo	0.586908906	Low-middle SDI
171	Democratic Republic of the Congo	0.390178166	Low SDI
172	Equatorial Guinea	0.663978286	Middle SDI
173	Gabon	0.639080604	Middle SDI
174	Eastern Sub-Saharan Africa	0.412187942	Low SDI
175	Burundi	0.291288817	Low SDI
176	Comoros	0.476955685	Low-middle SDI

Location ID	Location Name	2021 SDI Index Value	SDI Quintile
177	Djibouti	0.489200321	Low-middle SDI
178	Eritrea	0.404572056	Low SDI
179	Ethiopia	0.360727644	Low SDI
180	Kenya	0.524783146	Low-middle SDI
181	Madagascar	0.401385119	Low SDI
182	Malawi	0.381985594	Low SDI
183	Mauritius	0.717977109	High-middle SDI
184	Mozambique	0.327475463	Low SDI
185	Rwanda	0.436140248	Low SDI
186	Seychelles	0.727579445	High-middle SDI
187	Somalia	0.077433678	Low SDI
189	United Republic of Tanzania	0.448565569	Low SDI
190	Uganda	0.426553554	Low SDI
191	Zambia	0.510230369	Low-middle SDI
192	Southern Sub-Saharan Africa	0.643347819	Middle SDI
193	Botswana	0.643077969	Middle SDI

Location ID	Location Name	2021 SDI Index Value	SDI Quintile
194	Lesotho	0.51157061	Low-middle SDI
195	Namibia	0.618073651	Low-middle SDI
196	South Africa	0.681292244	Middle SDI
197	Eswatini	0.586216849	Low-middle SDI
198	Zimbabwe	0.475577138	Low-middle SDI
199	Western Sub-Saharan Africa	0.446420999	Low SDI
200	Benin	0.37452237	Low SDI
201	Burkina Faso	0.284470947	Low SDI
202	Cameroon	0.480364523	Low-middle SDI
203	Cabo Verde	0.533600978	Low-middle SDI
204	Chad	0.243516859	Low SDI
205	Côte d'Ivoire	0.424540566	Low SDI
206	Gambia	0.410077462	Low SDI
207	Ghana	0.563348184	Low-middle SDI
208	Guinea	0.336555329	Low SDI
209	Guinea-Bissau	0.353448423	Low SDI

Location ID	Location Name	2021 SDI Index Value	SDI Quintile
210	Liberia	0.353229409	Low SDI
211	Mali	0.271175692	Low SDI
212	Mauritania	0.495266784	Low-middle SDI
213	Niger	0.170310328	Low SDI
214	Nigeria	0.503698612	Low-middle SDI
215	Sao Tome and Principe	0.503305577	Low-middle SDI
216	Senegal	0.409005254	Low SDI
217	Sierra Leone	0.35900867	Low SDI
218	Togo	0.410016394	Low SDI
298	American Samoa	0.726267628	High-middle SDI
305	Bermuda	0.821319794	High SDI
320	Cook Islands	0.778251758	High-middle SDI
349	Greenland	0.835640003	High SDI
351	Guam	0.80216771	High-middle SDI
354	Hong Kong Special Administrative Region of China	0.850450848	High SDI
361	Macao Special Administrative Region of China	0.874759481	High SDI

Location ID	Location Name	2021 SDI Index Value	SDI Quintile
367	Monaco	0.909519124	High SDI
369	Nauru	0.627549782	Middle SDI
374	Niue	0.72621855	High-middle SDI
376	Northern Mariana Islands	0.777504838	High-middle SDI
380	Palau	0.754590186	High-middle SDI
385	Puerto Rico	0.824543903	High SDI
393	Saint Kitts and Nevis	0.756332641	High-middle SDI
396	San Marino	0.887883596	High SDI
413	Tokelau	0.68701842	Middle SDI
416	Tuvalu	0.578627145	Low-middle SDI
422	United States Virgin Islands	0.822988043	High SDI
433	Northern Ireland	0.841529955	High SDI
434	Scotland	0.853887319	High SDI
435	South Sudan	0.278377554	Low SDI
482	Eastern Cape	0.619101287	Middle SDI
483	Free State	0.678893967	Middle SDI

Location ID	Location Name	2021 SDI Index Value	SDI Quintile
484	Gauteng	0.736905342	High-middle SDI
485	KwaZulu-Natal	0.662386215	Middle SDI
486	Limpopo	0.613431617	Low-middle SDI
487	Mpumalanga	0.648324523	Middle SDI
488	North-West	0.654616033	Middle SDI
489	Northern Cape	0.665813245	Middle SDI
490	Western Cape	0.719732052	High-middle SDI
491	Anhui	0.661241263	Middle SDI
492	Beijing	0.845876468	High SDI
493	Chongqing	0.713248108	High-middle SDI
494	Fujian	0.715426566	High-middle SDI
495	Gansu	0.629648423	Middle SDI
496	Guangdong	0.766590014	High-middle SDI
497	Guangxi	0.677747142	Middle SDI
498	Guizhou	0.622926981	Middle SDI
499	Hainan	0.70526301	Middle SDI

Location ID	Location Name	2021 SDI Index Value	SDI Quintile
500	Hebei	0.703535825	Middle SDI
501	Heilongjiang	0.712062797	High-middle SDI
502	Henan	0.699479878	Middle SDI
503	Hubei	0.711832348	Middle SDI
504	Hunan	0.692120518	Middle SDI
505	Inner Mongolia	0.731684517	High-middle SDI
506	Jiangsu	0.755114988	High-middle SDI
507	Jiangxi	0.678215672	Middle SDI
508	Jilin	0.735628033	High-middle SDI
509	Liaoning	0.744555054	High-middle SDI
510	Ningxia	0.695319453	Middle SDI
511	Qinghai	0.648309483	Middle SDI
512	Shaanxi	0.724147628	High-middle SDI
513	Shandong	0.725623497	High-middle SDI
514	Shanghai	0.828661817	High SDI
515	Shanxi	0.711816196	Middle SDI

Location ID	Location Name	2021 SDI Index Value	SDI Quintile
516	Sichuan	0.672696014	Middle SDI
517	Tianjin	0.833804693	High SDI
518	Tibet	0.483893412	Low-middle SDI
519	Xinjiang	0.70686651	Middle SDI
520	Yunnan	0.627205795	Middle SDI
521	Zhejiang	0.742041363	High-middle SDI
522	Sudan	0.542748299	Low-middle SDI
523	Alabama	0.826417909	High SDI
524	Alaska	0.857125567	High SDI
525	Arizona	0.847683473	High SDI
526	Arkansas	0.816754461	High SDI
527	California	0.871090459	High SDI
528	Colorado	0.875986347	High SDI
529	Connecticut	0.901972117	High SDI
530	Delaware	0.866079256	High SDI
531	District of Columbia	0.907426863	High SDI

Location ID	Location Name	2021 SDI Index Value	SDI Quintile
532	Florida	0.861825164	High SDI
533	Georgia	0.847268118	High SDI
534	Hawaii	0.87084045	High SDI
535	Idaho	0.836495322	High SDI
536	Illinois	0.880611434	High SDI
537	Indiana	0.844050669	High SDI
538	Iowa	0.864342086	High SDI
539	Kansas	0.858890931	High SDI
540	Kentucky	0.821720983	High SDI
541	Louisiana	0.826669718	High SDI
542	Maine	0.866792716	High SDI
543	Maryland	0.891055635	High SDI
544	Massachusetts	0.90725037	High SDI
545	Michigan	0.864940748	High SDI
546	Minnesota	0.887884435	High SDI
547	Mississippi	0.811867151	High SDI

Location ID	Location Name	2021 SDI Index Value	SDI Quintile
548	Missouri	0.849044295	High SDI
549	Montana	0.859517184	High SDI
550	Nebraska	0.865629234	High SDI
551	Nevada	0.847864111	High SDI
552	New Hampshire	0.898526447	High SDI
553	New Jersey	0.891850577	High SDI
554	New Mexico	0.832846305	High SDI
555	New York	0.88592619	High SDI
556	North Carolina	0.846173734	High SDI
557	North Dakota	0.876134627	High SDI
558	Ohio	0.851227042	High SDI
559	Oklahoma	0.82814491	High SDI
560	Oregon	0.870189511	High SDI
561	Pennsylvania	0.873950359	High SDI
562	Rhode Island	0.884283653	High SDI
563	South Carolina	0.838586487	High SDI

Location ID	Location Name	2021 SDI Index Value	SDI Quintile
564	South Dakota	0.856263782	High SDI
565	Tennessee	0.831968835	High SDI
566	Texas	0.836777383	High SDI
567	Utah	0.854829295	High SDI
568	Vermont	0.89152237	High SDI
569	Virginia	0.881907315	High SDI
570	Washington	0.878013634	High SDI
571	West Virginia	0.82033351	High SDI
572	Wisconsin	0.873095963	High SDI
573	Wyoming	0.863142903	High SDI
4618	North East England	0.825842759	High SDI
4619	North West England	0.843295382	High SDI
4620	Yorkshire and the Humber	0.836407087	High SDI
4621	East Midlands	0.835023214	High SDI
4622	West Midlands	0.834648192	High SDI
4623	East of England	0.85577408	High SDI

Location ID	Location Name	2021 SDI Index Value	SDI Quintile
4624	Greater London	0.9041535	High SDI
4625	South East England	0.875365568	High SDI
4626	South West England	0.860849638	High SDI
4636	Wales	0.833274667	High SDI
4643	Aguascalientes	0.682557435	Middle SDI
4644	Baja California	0.704776585	Middle SDI
4645	Baja California Sur	0.710175355	Middle SDI
4646	Campeche	0.665087938	Middle SDI
4647	Coahuila	0.678075116	Middle SDI
4648	Colima	0.699338436	Middle SDI
4649	Chiapas	0.569756592	Low-middle SDI
4650	Chihuahua	0.674472052	Middle SDI
4651	Mexico City	0.759378377	High-middle SDI
4652	Durango	0.640562517	Middle SDI
4653	Guanajuato	0.647044734	Middle SDI
4654	Guerrero	0.584126986	Low-middle SDI

Location ID	Location Name	2021 SDI Index Value	SDI Quintile
4655	Hidalgo	0.633128071	Middle SDI
4656	Jalisco	0.677078025	Middle SDI
4657	México	0.681505383	Middle SDI
4658	Michoacán de Ocampo	0.613949206	Low-middle SDI
4659	Morelos	0.670104932	Middle SDI
4660	Nayarit	0.657928691	Middle SDI
4661	Nuevo León	0.712152517	High-middle SDI
4662	Oaxaca	0.588389144	Low-middle SDI
4663	Puebla	0.622884968	Middle SDI
4664	Querétaro	0.684048164	Middle SDI
4665	Quintana Roo	0.682591131	Middle SDI
4666	San Luis Potosí	0.647579585	Middle SDI
4667	Sinaloa	0.678348037	Middle SDI
4668	Sonora	0.709903997	Middle SDI
4669	Tabasco	0.649850519	Middle SDI
4670	Tamaulipas	0.682912198	Middle SDI

Location ID	Location Name	2021 SDI Index Value	SDI Quintile
4671	Tlaxcala	0.64898896	Middle SDI
4672	Veracruz de Ignacio de la Llave	0.627366789	Middle SDI
4673	Yucatán	0.654459012	Middle SDI
4674	Zacatecas	0.636670528	Middle SDI
4709	Aceh	0.671759479	Middle SDI
4710	North Sumatra	0.669499052	Middle SDI
4711	West Sumatra	0.66784543	Middle SDI
4712	Riau	0.724215125	High-middle SDI
4713	Jambi	0.640779478	Middle SDI
4714	South Sumatra	0.646421244	Middle SDI
4715	Bengkulu	0.613885329	Low-middle SDI
4716	Lampung	0.60891275	Low-middle SDI
4717	Bangka-Belitung Islands	0.644388463	Middle SDI
4718	Riau Islands	0.749803315	High-middle SDI
4719	North Kalimantan	0.754016402	High-middle SDI
4720	Jakarta	0.801237549	High-middle SDI

Location ID	Location Name	2021 SDI Index Value	SDI Quintile
4721	West Java	0.644279321	Middle SDI
4722	Central Java	0.613780247	Low-middle SDI
4723	Yogyakarta	0.676829859	Middle SDI
4724	East Java	0.646540022	Middle SDI
4725	Banten	0.641544087	Middle SDI
4726	Bali	0.652382779	Middle SDI
4727	West Nusa Tenggara	0.587663369	Low-middle SDI
4728	East Nusa Tenggara	0.550545868	Low-middle SDI
4729	West Kalimantan	0.587438981	Low-middle SDI
4730	Central Kalimantan	0.639931265	Middle SDI
4731	South Kalimantan	0.622221912	Middle SDI
4732	East Kalimantan	0.761652368	High-middle SDI
4733	North Sulawesi	0.652614588	Middle SDI
4734	Central Sulawesi	0.617544908	Low-middle SDI
4735	South Sulawesi	0.622994177	Middle SDI
4736	Southeast Sulawesi	0.618324728	Low-middle SDI

Location ID	Location Name	2021 SDI Index Value	SDI Quintile
4737	Gorontalo	0.571050074	Low-middle SDI
4738	West Sulawesi	0.576458969	Low-middle SDI
4739	Maluku	0.581624279	Low-middle SDI
4740	North Maluku	0.563444978	Low-middle SDI
4741	West Papua	0.676555222	Middle SDI
4742	Papua	0.646553603	Middle SDI
4749	England	0.860552694	High SDI
4750	Acre	0.562074727	Low-middle SDI
4751	Alagoas	0.529742892	Low-middle SDI
4752	Amazonas	0.603585976	Low-middle SDI
4753	Amapá	0.629807813	Middle SDI
4754	Bahia	0.574142222	Low-middle SDI
4755	Ceará	0.563912693	Low-middle SDI
4756	Distrito Federal	0.776152007	High-middle SDI
4757	Espírito Santo	0.667428625	Middle SDI
4758	Goiás	0.639347711	Middle SDI

Location ID	Location Name	2021 SDI Index Value	SDI Quintile
4759	Maranhão	0.49216193	Low-middle SDI
4760	Minas Gerais	0.648904701	Middle SDI
4761	Mato Grosso do Sul	0.642693307	Middle SDI
4762	Mato Grosso	0.647043759	Middle SDI
4763	Pará	0.577314362	Low-middle SDI
4764	Paraíba	0.557922296	Low-middle SDI
4765	Paraná	0.669860641	Middle SDI
4766	Pernambuco	0.583214758	Low-middle SDI
4767	Piaui	0.520291625	Low-middle SDI
4768	Rio de Janeiro	0.710470527	Middle SDI
4769	Rio Grande do Norte	0.585328241	Low-middle SDI
4770	Rondônia	0.618510473	Low-middle SDI
4771	Roraima	0.609883228	Low-middle SDI
4772	Rio Grande do Sul	0.689722837	Middle SDI
4773	Santa Catarina	0.694839624	Middle SDI
4774	Sergipe	0.590589987	Low-middle SDI

Location ID	Location Name	2021 SDI Index Value	SDI Quintile
4775	São Paulo	0.711182598	Middle SDI
4776	Tocantins	0.601403	Low-middle SDI
4841	Andhra Pradesh	0.552472881	Low-middle SDI
4842	Arunachal Pradesh	0.585180701	Low-middle SDI
4843	Assam	0.573442541	Low-middle SDI
4844	Bihar	0.45805086	Low SDI
4846	Chhattisgarh	0.540359862	Low-middle SDI
4849	Delhi	0.728650031	High-middle SDI
4850	Goa	0.724320503	High-middle SDI
4851	Gujarat	0.622975241	Middle SDI
4852	Haryana	0.629602575	Middle SDI
4853	Himachal Pradesh	0.643033305	Middle SDI
4854	Jammu & Kashmir and Ladakh	0.609583681	Low-middle SDI
4855	Jharkhand	0.5242297	Low-middle SDI
4856	Karnataka	0.585910724	Low-middle SDI
4857	Kerala	0.666417241	Middle SDI

Location ID	Location Name	2021 SDI Index Value	SDI Quintile
4859	Madhya Pradesh	0.531115567	Low-middle SDI
4860	Maharashtra	0.639494993	Middle SDI
4861	Manipur	0.584558179	Low-middle SDI
4862	Meghalaya	0.560405068	Low-middle SDI
4863	Mizoram	0.62843683	Middle SDI
4864	Nagaland	0.637648412	Middle SDI
4865	Odisha	0.551364782	Low-middle SDI
4867	Punjab	0.632901572	Middle SDI
4868	Rajasthan	0.536745684	Low-middle SDI
4869	Sikkim	0.635190296	Middle SDI
4870	Tamil Nadu	0.629914474	Middle SDI
4871	Telangana	0.567480596	Low-middle SDI
4872	Tripura	0.555782348	Low-middle SDI
4873	Uttar Pradesh	0.527579329	Low-middle SDI
4874	Uttarakhand	0.661804198	Middle SDI
4875	West Bengal	0.556791581	Low-middle SDI

Location ID	Location Name	2021 SDI Index Value	SDI Quintile
4910	Oslo	0.947286104	High SDI
4920	Rogaland	0.916764681	High SDI
4923	Møre og Romsdal	0.908149197	High SDI
4926	Nordland	0.898213885	High SDI
4940	Sweden except Stockholm	0.875759008	High SDI
4944	Stockholm	0.916765403	High SDI
25318	Abia	0.629261465	Middle SDI
25319	Adamawa	0.474621913	Low-middle SDI
25320	Akwa Ibom	0.589407255	Low-middle SDI
25321	Anambra	0.62862687	Middle SDI
25322	Bauchi	0.294070246	Low SDI
25323	Bayelsa	0.558802657	Low-middle SDI
25324	Benue	0.49859261	Low-middle SDI
25325	Borno	0.398589587	Low SDI
25326	Cross River	0.562249026	Low-middle SDI
25327	Delta	0.614789164	Low-middle SDI

Location ID	Location Name	2021 SDI Index Value	SDI Quintile
25328	Ebonyi	0.540874531	Low-middle SDI
25329	Edo	0.6270757	Middle SDI
25330	Ekiti	0.609689218	Low-middle SDI
25331	Enugu	0.60872942	Low-middle SDI
25332	FCT (Abuja)	0.621406693	Middle SDI
25333	Gombe	0.379904259	Low SDI
25334	Imo	0.629384576	Middle SDI
25335	Jigawa	0.287216991	Low SDI
25336	Kaduna	0.442437011	Low SDI
25337	Kano	0.408256207	Low SDI
25338	Katsina	0.297115378	Low SDI
25339	Kebbi	0.284501095	Low SDI
25340	Kogi	0.560608509	Low-middle SDI
25341	Kwara	0.536539828	Low-middle SDI
25342	Lagos	0.679573249	Middle SDI
25343	Nasarawa	0.518505244	Low-middle SDI

Location ID	Location Name	2021 SDI Index Value	SDI Quintile
25344	Niger	0.42518919	Low SDI
25345	Ogun	0.569648186	Low-middle SDI
25346	Ondo	0.592384037	Low-middle SDI
25347	Osun	0.627939478	Middle SDI
25348	Oyo	0.584428907	Low-middle SDI
25349	Plateau	0.530865998	Low-middle SDI
25350	Rivers	0.638605802	Middle SDI
25351	Sokoto	0.254081326	Low SDI
25352	Taraba	0.438659637	Low SDI
25353	Yobe	0.312002634	Low SDI
25354	Zamfara	0.267800369	Low SDI
35424	Hokkaidō	0.844008657	High SDI
35425	Aomori	0.828693902	High SDI
35426	Iwate	0.835652812	High SDI
35427	Miyagi	0.860058983	High SDI
35428	Akita	0.832380775	High SDI

Location ID	Location Name	2021 SDI Index Value	SDI Quintile
35429	Yamagata	0.838535343	High SDI
35430	Fukushima	0.841218485	High SDI
35431	Ibaraki	0.860523394	High SDI
35432	Tochigi	0.861256293	High SDI
35433	Gunma	0.861770439	High SDI
35434	Saitama	0.85655373	High SDI
35435	Chiba	0.861645419	High SDI
35436	Tōkyō	0.929043198	High SDI
35437	Kanagawa	0.882743596	High SDI
35438	Niigata	0.845233683	High SDI
35439	Toyama	0.865453766	High SDI
35440	Ishikawa	0.860214615	High SDI
35441	Fukui	0.856579697	High SDI
35442	Yamanashi	0.858173518	High SDI
35443	Nagano	0.858966672	High SDI
35444	Gifu	0.853447894	High SDI

Location ID	Location Name	2021 SDI Index Value	SDI Quintile
35445	Shizuoka	0.865720709	High SDI
35446	Aichi	0.883329296	High SDI
35447	Mie	0.860795402	High SDI
35448	Shiga	0.874377981	High SDI
35449	Kyōto	0.876288854	High SDI
35450	Ōsaka	0.876433199	High SDI
35451	Hyōgo	0.868490782	High SDI
35452	Nara	0.851417382	High SDI
35453	Wakayama	0.847330156	High SDI
35454	Tottori	0.836091465	High SDI
35455	Shimane	0.838873648	High SDI
35456	Okayama	0.862350161	High SDI
35457	Hiroshima	0.870170469	High SDI
35458	Yamaguchi	0.856635725	High SDI
35459	Tokushima	0.85729815	High SDI
35460	Kagawa	0.857948449	High SDI

Location ID	Location Name	2021 SDI Index Value	SDI Quintile
35461	Ehime	0.843779842	High SDI
35462	Kōchi	0.835890247	High SDI
35463	Fukuoka	0.858415047	High SDI
35464	Saga	0.835872528	High SDI
35465	Nagasaki	0.829106338	High SDI
35466	Kumamoto	0.834728198	High SDI
35467	Ōita	0.848715974	High SDI
35468	Miyazaki	0.826768586	High SDI
35469	Kagoshima	0.832472168	High SDI
35470	Okinawa	0.821640127	High SDI
35494	Piemonte	0.806813454	High-middle SDI
35495	Valle d'Aosta	0.812706016	High SDI
35496	Liguria	0.821521126	High SDI
35497	Lombardia	0.829091495	High SDI
35498	Provincia autonoma di Bolzano	0.838907386	High SDI
35499	Provincia autonoma di Trento	0.829531919	High SDI

Location ID	Location Name	2021 SDI Index Value	SDI Quintile
35500	Veneto	0.808196606	High-middle SDI
35501	Friuli-Venezia Giulia	0.819858092	High SDI
35502	Emilia-Romagna	0.829695157	High SDI
35503	Toscana	0.811485035	High SDI
35504	Umbria	0.799092255	High-middle SDI
35505	Marche	0.803749028	High-middle SDI
35506	Lazio	0.827195546	High SDI
35507	Abruzzo	0.816496827	High SDI
35508	Molise	0.787791453	High-middle SDI
35509	Campania	0.766832076	High-middle SDI
35510	Puglia	0.763946911	High-middle SDI
35511	Basilicata	0.783798467	High-middle SDI
35512	Calabria	0.775304844	High-middle SDI
35513	Sicilia	0.76255912	High-middle SDI
35514	Sardegna	0.772644153	High-middle SDI
35617	Baringo	0.514621991	Low-middle SDI

Location ID	Location Name	2021 SDI Index Value	SDI Quintile
35618	Bomet	0.530035509	Low-middle SDI
35619	Bungoma	0.488274636	Low-middle SDI
35620	Busia	0.478169739	Low-middle SDI
35621	Elgeyo-Marakwet	0.525285148	Low-middle SDI
35622	Embu	0.548377806	Low-middle SDI
35623	Garissa	0.32165019	Low SDI
35624	Homa Bay	0.507694781	Low-middle SDI
35625	Isiolo	0.435133975	Low SDI
35626	Kajiado	0.501634865	Low-middle SDI
35627	Kakamega	0.508518514	Low-middle SDI
35628	Kericho	0.520411743	Low-middle SDI
35629	Kiambu	0.593046232	Low-middle SDI
35630	Kilifi	0.486686815	Low-middle SDI
35631	Kirinyaga	0.546740689	Low-middle SDI
35632	Kisii	0.549398782	Low-middle SDI
35633	Kisumu	0.548720604	Low-middle SDI

Location ID	Location Name	2021 SDI Index Value	SDI Quintile
35634	Kitui	0.474177297	Low-middle SDI
35635	Kwale	0.47965716	Low-middle SDI
35636	Laikipia	0.576686523	Low-middle SDI
35637	Lamu	0.505575183	Low-middle SDI
35638	Machakos	0.55015778	Low-middle SDI
35639	Makueni	0.514230695	Low-middle SDI
35640	Mandera	0.239926734	Low SDI
35641	Marsabit	0.399976958	Low SDI
35642	Meru	0.509152437	Low-middle SDI
35643	Migori	0.482207014	Low-middle SDI
35644	Mombasa	0.598166665	Low-middle SDI
35645	Murang'a	0.55292203	Low-middle SDI
35646	Nairobi	0.684188978	Middle SDI
35647	Nakuru	0.5721094	Low-middle SDI
35648	Nandi	0.516039708	Low-middle SDI
35649	Narok	0.458227005	Low SDI

Location ID	Location Name	2021 SDI Index Value	SDI Quintile
35650	Nyamira	0.593029203	Low-middle SDI
35651	Nyandarua	0.577540201	Low-middle SDI
35652	Nyeri	0.579362636	Low-middle SDI
35653	Samburu	0.371475608	Low SDI
35654	Siaya	0.484421932	Low-middle SDI
35655	Taita Taveta	0.542579215	Low-middle SDI
35656	Tana River	0.389358757	Low SDI
35657	Tharaka Nithi	0.528624517	Low-middle SDI
35658	Trans Nzoia	0.549784994	Low-middle SDI
35659	Turkana	0.3683857	Low SDI
35660	Uasin Gishu	0.567922916	Low-middle SDI
35661	Vihiga	0.527054687	Low-middle SDI
35662	Wajir	0.258713923	Low SDI
35663	West Pokot	0.44769021	Low SDI
43872	Andhra Pradesh, Urban	0.647551072	Middle SDI
43873	Arunachal Pradesh, Urban	0.68419449	Middle SDI

Location ID	Location Name	2021 SDI Index Value	SDI Quintile
43874	Assam, Urban	0.675446354	Middle SDI
43875	Bihar, Urban	0.591183966	Low-middle SDI
43877	Chhattisgarh, Urban	0.679763349	Middle SDI
43880	Delhi, Urban	0.730151189	High-middle SDI
43881	Goa, Urban	0.741223296	High-middle SDI
43882	Gujarat, Urban	0.703282867	Middle SDI
43883	Haryana, Urban	0.703932889	Middle SDI
43884	Himachal Pradesh, Urban	0.748161993	High-middle SDI
43885	Jammu & Kashmir and Ladakh, Urban	0.684343769	Middle SDI
43886	Jharkhand, Urban	0.670458961	Middle SDI
43887	Karnataka, Urban	0.658831954	Middle SDI
43888	Kerala, Urban	0.672698917	Middle SDI
43890	Madhya Pradesh, Urban	0.675329604	Middle SDI
43891	Maharashtra, Urban	0.705231591	Middle SDI
43892	Manipur, Urban	0.632756357	Middle SDI
43893	Meghalaya, Urban	0.685725867	Middle SDI

Location ID	Location Name	2021 SDI Index Value	SDI Quintile
43894	Mizoram, Urban	0.670528706	Middle SDI
43895	Nagaland, Urban	0.691617405	Middle SDI
43896	Odisha, Urban	0.657514066	Middle SDI
43898	Punjab, Urban	0.698430939	Middle SDI
43899	Rajasthan, Urban	0.671757713	Middle SDI
43900	Sikkim, Urban	0.695740102	Middle SDI
43901	Tamil Nadu, Urban	0.676173806	Middle SDI
43902	Telangana, Urban	0.667241582	Middle SDI
43903	Tripura, Urban	0.633348097	Middle SDI
43904	Uttar Pradesh, Urban	0.63211101	Middle SDI
43905	Uttarakhand, Urban	0.746776963	High-middle SDI
43906	West Bengal, Urban	0.6493937	Middle SDI
43908	Andhra Pradesh, Rural	0.495920251	Low-middle SDI
43909	Arunachal Pradesh, Rural	0.549469207	Low-middle SDI
43910	Assam, Rural	0.551639812	Low-middle SDI
43911	Bihar, Rural	0.434277772	Low SDI

Location ID	Location Name	2021 SDI Index Value	SDI Quintile
43913	Chhattisgarh, Rural	0.486779046	Low-middle SDI
43916	Delhi, Rural	0.655467765	Middle SDI
43917	Goa, Rural	0.687517157	Middle SDI
43918	Gujarat, Rural	0.549296846	Low-middle SDI
43919	Haryana, Rural	0.579178153	Low-middle SDI
43920	Himachal Pradesh, Rural	0.629512224	Middle SDI
43921	Jammu & Kashmir and Ladakh, Rural	0.572664356	Low-middle SDI
43922	Jharkhand, Rural	0.463971644	Low SDI
43923	Karnataka, Rural	0.526848679	Low-middle SDI
43924	Kerala, Rural	0.659487084	Middle SDI
43926	Madhya Pradesh, Rural	0.464482329	Low SDI
43927	Maharashtra, Rural	0.574895865	Low-middle SDI
43928	Manipur, Rural	0.560840633	Low-middle SDI
43929	Meghalaya, Rural	0.519625901	Low-middle SDI
43930	Mizoram, Rural	0.57456484	Low-middle SDI
43931	Nagaland, Rural	0.600219084	Low-middle SDI

Location ID	Location Name	2021 SDI Index Value	SDI Quintile
43932	Odisha, Rural	0.520670909	Low-middle SDI
43934	Punjab, Rural	0.585512634	Low-middle SDI
43935	Rajasthan, Rural	0.478194737	Low-middle SDI
43936	Sikkim, Rural	0.593976905	Low-middle SDI
43937	Tamil Nadu, Rural	0.580536414	Low-middle SDI
43938	Telangana, Rural	0.483176983	Low-middle SDI
43939	Tripura, Rural	0.515999282	Low-middle SDI
43940	Uttar Pradesh, Rural	0.490024946	Low-middle SDI
43941	Uttarakhand, Rural	0.61464175	Low-middle SDI
43942	West Bengal, Rural	0.502428983	Low-middle SDI
44533	China (without Hong Kong and Macao)	0.717406624	High-middle SDI
44538	Other Union Territories	0.676062684	Middle SDI
44539	Other Union Territories, Rural	0.601933292	Low-middle SDI
44540	Other Union Territories, Urban	0.708204745	Middle SDI
44643	Darlington	0.835427723	High SDI
44644	Northumberland	0.822333819	High SDI

Location ID	Location Name	2021 SDI Index Value	SDI Quintile
44645	Stockton-on-Tees	0.829517602	High SDI
44646	Newcastle upon Tyne	0.871588115	High SDI
44647	North Tyneside	0.835247504	High SDI
44648	Redcar and Cleveland	0.796360243	High-middle SDI
44649	County Durham	0.810754624	High SDI
44650	Gateshead	0.828579746	High SDI
44651	Middlesbrough	0.798976656	High-middle SDI
44652	South Tyneside	0.799489743	High-middle SDI
44653	Sunderland	0.817933435	High SDI
44654	Hartlepool	0.797297174	High-middle SDI
44655	Cheshire East	0.884278805	High SDI
44656	Stockport	0.861014705	High SDI
44657	Trafford	0.896679044	High SDI
44658	Cheshire West and Chester	0.870242062	High SDI
44659	Sefton	0.825438177	High SDI
44660	Lancashire	0.839626361	High SDI

Location ID	Location Name	2021 SDI Index Value	SDI Quintile
44661	Cumbria	0.842325739	High SDI
44662	Bolton	0.812595954	High SDI
44663	Wirral	0.818111684	High SDI
44664	Bury	0.828816843	High SDI
44665	St Helens	0.810914124	High SDI
44666	Warrington	0.878129342	High SDI
44667	Oldham	0.796704511	High-middle SDI
44668	Rochdale	0.800322186	High-middle SDI
44669	Wigan	0.80548334	High-middle SDI
44670	Halton	0.835687566	High SDI
44671	Liverpool	0.847483043	High SDI
44672	Tameside	0.798557943	High-middle SDI
44673	Salford	0.837393179	High SDI
44674	Blackburn with Darwen	0.810134059	High SDI
44675	Knowsley	0.811985259	High SDI
44676	Blackpool	0.788904606	High-middle SDI

Location ID	Location Name	2021 SDI Index Value	SDI Quintile
44677	Manchester	0.88057256	High SDI
44678	North Yorkshire	0.855520996	High SDI
44679	East Riding of Yorkshire	0.835193656	High SDI
44680	York	0.888110353	High SDI
44681	North East Lincolnshire	0.803563778	High-middle SDI
44682	Calderdale	0.836996359	High SDI
44683	North Lincolnshire	0.824582845	High SDI
44684	Bradford	0.814923693	High SDI
44685	Kirklees	0.823525465	High SDI
44686	Leeds	0.86787974	High SDI
44687	Sheffield	0.854247178	High SDI
44688	Wakefield	0.804506533	High-middle SDI
44689	Rotherham	0.803331209	High-middle SDI
44690	Doncaster	0.793628472	High-middle SDI
44691	Kingston upon Hull, City of	0.797611011	High-middle SDI
44692	Barnsley	0.788111758	High-middle SDI

Location ID	Location Name	2021 SDI Index Value	SDI Quintile
44693	Northamptonshire	0.839033237	High SDI
44694	Leicestershire	0.851100665	High SDI
44695	Lincolnshire	0.820522815	High SDI
44696	Rutland	0.852195785	High SDI
44697	Derby	0.844076935	High SDI
44698	Derbyshire	0.823174583	High SDI
44699	Nottinghamshire	0.822432787	High SDI
44700	Nottingham	0.858455251	High SDI
44701	Leicester	0.828051202	High SDI
44702	Warwickshire	0.865693767	High SDI
44703	Herefordshire, County of	0.846194561	High SDI
44704	Solihull	0.871566638	High SDI
44705	Shropshire	0.842380445	High SDI
44706	Worcestershire	0.842681308	High SDI
44707	Staffordshire	0.828181727	High SDI
44708	Dudley	0.802585915	High-middle SDI

Location ID	Location Name	2021 SDI Index Value	SDI Quintile
44709	Coventry	0.847335743	High SDI
44710	Telford and Wrekin	0.826093832	High SDI
44711	Stoke-on-Trent	0.796441727	High-middle SDI
44712	Walsall	0.790654736	High-middle SDI
44713	Wolverhampton	0.810887602	High SDI
44714	Birmingham	0.836949232	High SDI
44715	Sandwell	0.793668776	High-middle SDI
44716	Bedford	0.856962284	High SDI
44717	Central Bedfordshire	0.851318833	High SDI
44718	Suffolk	0.840388992	High SDI
44719	Hertfordshire	0.886963263	High SDI
44720	Essex	0.844953071	High SDI
44721	Cambridgeshire	0.887630336	High SDI
44722	Thurrock	0.818629818	High SDI
44723	Norfolk	0.836988602	High SDI
44724	Southend-on-Sea	0.825018651	High SDI

Location ID	Location Name	2021 SDI Index Value	SDI Quintile
44725	Peterborough	0.837008458	High SDI
44726	Luton	0.838003231	High SDI
44727	Richmond upon Thames	0.932021729	High SDI
44728	Kensington and Chelsea	0.946366051	High SDI
44729	Barnet	0.885110546	High SDI
44730	Westminster	0.93701032	High SDI
44731	Bromley	0.869193749	High SDI
44732	Bexley	0.844097911	High SDI
44733	Redbridge	0.849278219	High SDI
44734	Merton	0.887252238	High SDI
44735	Brent	0.858683624	High SDI
44736	Hillingdon	0.892536477	High SDI
44737	Havering	0.834176853	High SDI
44738	Kingston upon Thames	0.908394132	High SDI
44739	Sutton	0.857530229	High SDI
44740	Harrow	0.858244679	High SDI

Location ID	Location Name	2021 SDI Index Value	SDI Quintile
44741	Enfield	0.845516817	High SDI
44742	Croydon	0.851439949	High SDI
44743	Hammersmith and Fulham	0.934892187	High SDI
44744	Ealing	0.882007048	High SDI
44745	Greenwich	0.845443655	High SDI
44746	Wandsworth	0.924121015	High SDI
44747	Waltham Forest	0.840038834	High SDI
44748	Camden	0.936076172	High SDI
44749	Lambeth	0.916015975	High SDI
44750	Lewisham	0.85672931	High SDI
44751	Hounslow	0.896360928	High SDI
44752	Southwark	0.919165412	High SDI
44753	Newham	0.840477768	High SDI
44754	Barking and Dagenham	0.80642649	High-middle SDI
44755	Haringey	0.8714663	High SDI
44756	Hackney	0.891329222	High SDI

Location ID	Location Name	2021 SDI Index Value	SDI Quintile
44757	Islington	0.924302624	High SDI
44758	Tower Hamlets	0.903700654	High SDI
44759	Wokingham	0.910821902	High SDI
44760	Buckinghamshire	0.888192339	High SDI
44761	Surrey	0.904483995	High SDI
44762	Windsor and Maidenhead	0.915543426	High SDI
44763	West Berkshire	0.897124589	High SDI
44764	Hampshire	0.87199888	High SDI
44765	Bracknell Forest	0.890716234	High SDI
44766	West Sussex	0.863885895	High SDI
44767	Oxfordshire	0.899135231	High SDI
44768	Reading	0.90527187	High SDI
44769	Kent	0.844500542	High SDI
44770	Brighton and Hove	0.897901251	High SDI
44771	Medway	0.819461594	High SDI
44772	East Sussex	0.83898657	High SDI

Location ID	Location Name	2021 SDI Index Value	SDI Quintile
44773	Portsmouth	0.864778156	High SDI
44774	Isle of Wight	0.826023587	High SDI
44775	Milton Keynes	0.886756129	High SDI
44776	Southampton	0.860211008	High SDI
44777	Slough	0.877374496	High SDI
44778	South Gloucestershire	0.88411864	High SDI
44779	Dorset	0.8514167	High SDI
44780	Wiltshire	0.859020608	High SDI
44781	North Somerset	0.85864883	High SDI
44782	Devon	0.854622345	High SDI
44783	Poole	0.862990852	High SDI
44784	Bath and North East Somerset	0.895075988	High SDI
44785	Gloucestershire	0.870918382	High SDI
44786	Somerset	0.842820976	High SDI
44787	Swindon	0.86670182	High SDI
44788	Torbay	0.812459589	High SDI

Location ID	Location Name	2021 SDI Index Value	SDI Quintile
44789	Bristol, City of	0.896565514	High SDI
44790	Bournemouth	0.870149525	High SDI
44791	Cornwall	0.839219489	High SDI
44792	Plymouth	0.84217698	High SDI
44793	Central	0.577789494	Low-middle SDI
44794	Coast	0.518014043	Low-middle SDI
44795	Eastern	0.508217906	Low-middle SDI
44796	Nairobi	0.684188971	Middle SDI
44797	North Eastern	0.264360533	Low SDI
44798	Nyanza	0.525077558	Low-middle SDI
44799	Rift Valley	0.51425552	Low-middle SDI
44800	Western	0.504497721	Low-middle SDI
44850	New Zealand Maori population	0.770791784	High-middle SDI
44851	New Zealand non-Maori population	0.870812225	High SDI
44852	Tigray	0.38400255	Low SDI
44853	Afar	0.286502778	Low SDI

Location ID	Location Name	2021 SDI Index Value	SDI Quintile
44854	Amhara	0.322156172	Low SDI
44855	Oromia	0.337961203	Low SDI
44856	Somali	0.27014767	Low SDI
44857	Benishangul-Gumuz	0.323654895	Low SDI
44858	Southern Nations, Nationalities, and Peoples	0.357248469	Low SDI
44859	Harari	0.539739601	Low-middle SDI
44860	Gambella	0.460576186	Low SDI
44861	Addis Ababa	0.695287927	Middle SDI
44862	Dire Dawa	0.542617703	Low-middle SDI
44864	Alborz	0.748208954	High-middle SDI
44865	Ardebil	0.658777858	Middle SDI
44866	East Azarbayejan	0.667933993	Middle SDI
44867	West Azarbayejan	0.626918833	Middle SDI
44868	Bushehr	0.708677286	Middle SDI
44869	Chahar Mahaal and Bakhtiari	0.678339055	Middle SDI
44870	Fars	0.715109154	High-middle SDI

Location ID	Location Name	2021 SDI Index Value	SDI Quintile
44871	Gilan	0.712361968	High-middle SDI
44872	Golestan	0.656422158	Middle SDI
44873	Hamadan	0.666968762	Middle SDI
44874	Hormozgan	0.670775004	Middle SDI
44875	Ilam	0.705185457	Middle SDI
44876	Isfahan	0.709893952	Middle SDI
44877	Kerman	0.66878396	Middle SDI
44878	Kermanshah	0.674511267	Middle SDI
44879	North Khorasan	0.651483141	Middle SDI
44880	Khorasan-e-Razavi	0.67053956	Middle SDI
44881	South Khorasan	0.653364832	Middle SDI
44882	Khuzestan	0.669816556	Middle SDI
44883	Kohgiluyeh and Boyer-Ahmad	0.694488035	Middle SDI
44884	Kurdistan	0.642334326	Middle SDI
44885	Lorestan	0.669421197	Middle SDI
44886	Markazi	0.682789151	Middle SDI

Location ID	Location Name	2021 SDI Index Value	SDI Quintile
44887	Mazandaran	0.729935836	High-middle SDI
44888	Qazvin	0.687516004	Middle SDI
44889	Qom	0.694133773	Middle SDI
44890	Semnan	0.724001841	High-middle SDI
44891	Sistan and Baluchistan	0.549869409	Low-middle SDI
44892	Tehran	0.776102826	High-middle SDI
44893	Yazd	0.713637577	High-middle SDI
44894	Zanjan	0.661463751	Middle SDI
44903	Belgorod oblast	0.803695074	High-middle SDI
44904	Bryansk oblast	0.792329315	High-middle SDI
44905	Vladimir oblast	0.788797618	High-middle SDI
44906	Voronezh oblast	0.810406591	High SDI
44907	Ivanovo oblast	0.789631875	High-middle SDI
44908	Kaluga oblast	0.800527701	High-middle SDI
44909	Kostroma oblast	0.787756437	High-middle SDI
44910	Kursk oblast	0.793649987	High-middle SDI

Location ID	Location Name	2021 SDI Index Value	SDI Quintile
44911	Lipetzk oblast	0.802916176	High-middle SDI
44912	Moscow oblast	0.828120859	High SDI
44913	Oryol oblast	0.79451294	High-middle SDI
44914	Ryazan oblast	0.793693116	High-middle SDI
44915	Smolensk oblast	0.800950484	High-middle SDI
44916	Tambov oblast	0.790982828	High-middle SDI
44917	Tver oblast	0.799102213	High-middle SDI
44918	Tula oblast	0.797660828	High-middle SDI
44919	Yaroslavl oblast	0.805364359	High-middle SDI
44920	Moscow City	0.887772609	High SDI
44921	Republic of Karelia	0.81238914	High SDI
44922	Komi Republic	0.790079817	High-middle SDI
44923	Nenets autonomous district	0.800997369	High-middle SDI
44924	Arkhangelsk oblast without Nenets autonomous district	0.812017693	High SDI
44925	Vologda oblast	0.786532281	High-middle SDI
44926	Kaliningrad oblast	0.803693646	High-middle SDI

Location ID	Location Name	2021 SDI Index Value	SDI Quintile
44927	Leningrad oblast	0.814446452	High SDI
44928	Murmansk oblast	0.829529044	High SDI
44929	Novgorod oblast	0.779643289	High-middle SDI
44930	Pskov oblast	0.787746847	High-middle SDI
44931	Saint Petersburg	0.859435837	High SDI
44932	Republic of Adygeya	0.783355473	High-middle SDI
44933	Republic of Kalmykia	0.754503495	High-middle SDI
44934	Republic of Crimea	0.713851386	High-middle SDI
44935	Krasnodar kray	0.799215014	High-middle SDI
44936	Astrakhan oblast	0.777754786	High-middle SDI
44937	Volgograd oblast	0.792090776	High-middle SDI
44938	Rostov oblast	0.804560133	High-middle SDI
44939	Sevastopol	0.778562755	High-middle SDI
44940	Republic of Dagestan	0.75489971	High-middle SDI
44941	Republic of Ingushetia	0.723181052	High-middle SDI
44942	Kabardino-Balkar Republic	0.773368211	High-middle SDI

Location ID	Location Name	2021 SDI Index Value	SDI Quintile
44943	Karachay-Cherkess Republic	0.756823567	High-middle SDI
44944	Republic of North Ossetia-Alania	0.792451998	High-middle SDI
44945	Chechen Republic	0.697000298	Middle SDI
44946	Stavropol kray	0.785308224	High-middle SDI
44947	Republic of Bashkortostan	0.807048582	High-middle SDI
44948	Republic of Mari El	0.777484633	High-middle SDI
44949	Republic of Mordovia	0.77934414	High-middle SDI
44950	Republic of Tatarstan	0.815624973	High SDI
44951	Udmurt Republic	0.786843627	High-middle SDI
44952	Chuvash Republic	0.779169152	High-middle SDI
44953	Perm kray	0.796955473	High-middle SDI
44954	Kirov oblast	0.785897475	High-middle SDI
44955	Nizhny Novgorod oblast	0.81057854	High SDI
44956	Orenburg oblast	0.78313786	High-middle SDI
44957	Penza oblast	0.790346819	High-middle SDI
44958	Samara oblast	0.814000588	High SDI

Location ID	Location Name	2021 SDI Index Value	SDI Quintile
44959	Saratov oblast	0.785980203	High-middle SDI
44960	Ulyanovsk oblast	0.782641776	High-middle SDI
44961	Kurgan oblast	0.76552614	High-middle SDI
44962	Sverdlovsk oblast	0.816064746	High SDI
44963	Khanty-Mansi autonomous area	0.826709092	High SDI
44964	Yamalo-Nenets autonomous area	0.837700215	High SDI
44965	Tyumen oblast without autonomous areas	0.813017728	High SDI
44966	Chelyabinsk oblast	0.793022546	High-middle SDI
44967	Republic of Altai	0.749802645	High-middle SDI
44968	Republic of Buryatia	0.778616162	High-middle SDI
44969	Republic of Tuva	0.692513465	Middle SDI
44970	Republic of Khakassia	0.777318539	High-middle SDI
44971	Altai kray	0.777099855	High-middle SDI
44972	Zabaikalsk kray	0.740925147	High-middle SDI
44973	Krasnoyarsk kray	0.796852774	High-middle SDI
44974	Irkutsk oblast	0.77351672	High-middle SDI

Location ID	Location Name	2021 SDI Index Value	SDI Quintile
44975	Kemerovo oblast	0.782797668	High-middle SDI
44976	Novosibirsk oblast	0.796528925	High-middle SDI
44977	Omsk oblast	0.791260764	High-middle SDI
44978	Tomsk oblast	0.802325795	High-middle SDI
44979	Republic of Sakha (Yakutia)	0.814649347	High SDI
44980	Kamchatka kray	0.798113769	High-middle SDI
44981	Primorsky kray	0.802659761	High-middle SDI
44982	Khabarovsk kray	0.818047393	High SDI
44983	Amur oblast	0.793881993	High-middle SDI
44984	Magadan oblast	0.830546268	High SDI
44985	Sakhalin oblast	0.822655388	High SDI
44986	Jewish autonomous oblast	0.765463494	High-middle SDI
44987	Chukotka Autonomous Area	0.800770455	High-middle SDI
50559	Ukraine (without Crimea & Sevastopol)	0.761743094	High-middle SDI
53432	Trøndelag	0.916773946	High SDI
53533	Mountain Province	0.51921863	Low-middle SDI

Location ID	Location Name	2021 SDI Index Value	SDI Quintile
53534	Ifugao	0.597325624	Low-middle SDI
53535	Benguet	0.716293119	High-middle SDI
53536	Abra	0.654173651	Middle SDI
53537	Apayao	0.60770129	Low-middle SDI
53538	Kalinga	0.575239213	Low-middle SDI
53539	La Union	0.661006064	Middle SDI
53540	Ilocos Norte	0.687657015	Middle SDI
53541	Ilocos Sur	0.671836522	Middle SDI
53542	Pangasinan	0.666135554	Middle SDI
53543	Nueva Vizcaya	0.616609509	Low-middle SDI
53544	Cagayan	0.63433253	Middle SDI
53545	Isabela	0.636783935	Middle SDI
53546	Quirino	0.575885131	Low-middle SDI
53547	Batanes	0.682565947	Middle SDI
53548	Bataan	0.660374268	Middle SDI
53549	Zambales	0.65425423	Middle SDI

Location ID	Location Name	2021 SDI Index Value	SDI Quintile
53550	Tarlac	0.650760386	Middle SDI
53551	Pampanga	0.697271285	Middle SDI
53552	Bulacan	0.708002625	Middle SDI
53553	Nueva Ecija	0.650591609	Middle SDI
53554	Aurora	0.614063114	Low-middle SDI
53555	Rizal	0.710524418	Middle SDI
53556	Cavite	0.72917856	High-middle SDI
53557	Laguna	0.701620417	Middle SDI
53558	Batangas	0.686055208	Middle SDI
53559	Quezon	0.630168626	Middle SDI
53560	Occidental Mindoro	0.46028876	Low SDI
53561	Oriental Mindoro	0.60555167	Low-middle SDI
53562	Romblon	0.527572654	Low-middle SDI
53563	Palawan	0.527211201	Low-middle SDI
53564	Marinduque	0.549650294	Low-middle SDI
53565	Catanduanes	0.60942324	Low-middle SDI

Location ID	Location Name	2021 SDI Index Value	SDI Quintile
53566	Camarines Norte	0.594459098	Low-middle SDI
53567	Sorsogon	0.600776304	Low-middle SDI
53568	Albay	0.640151269	Middle SDI
53569	Masbate	0.458060424	Low SDI
53570	Camarines Sur	0.633743794	Middle SDI
53571	Capiz	0.5714003	Low-middle SDI
53572	Aklan	0.64042058	Middle SDI
53573	Antique	0.569823295	Low-middle SDI
53574	Negros Occidental	0.604235111	Low-middle SDI
53575	Iloilo	0.673508341	Middle SDI
53576	Guimaras	0.609860186	Low-middle SDI
53577	Negros Oriental	0.578181475	Low-middle SDI
53578	Cebu	0.658154663	Middle SDI
53579	Bohol	0.604933545	Low-middle SDI
53580	Siquijor	0.600945243	Low-middle SDI
53581	Southern Leyte	0.60149118	Low-middle SDI

Location ID	Location Name	2021 SDI Index Value	SDI Quintile
53582	Eastern Samar	0.499800385	Low-middle SDI
53583	Northern Samar	0.52396754	Low-middle SDI
53584	Samar (Western Samar)	0.527590959	Low-middle SDI
53585	Leyte	0.611701754	Low-middle SDI
53586	Biliran	0.643027685	Middle SDI
53587	Zamboanga Sibugay	0.549420679	Low-middle SDI
53588	Zamboanga Del Norte	0.53803222	Low-middle SDI
53589	Zamboanga Del Sur	0.630995266	Middle SDI
53590	Misamis Occidental	0.588184738	Low-middle SDI
53591	Bukidnon	0.551058807	Low-middle SDI
53592	Lanao Del Norte	0.587617211	Low-middle SDI
53593	Misamis Oriental	0.662873429	Middle SDI
53594	Camiguin	0.632089767	Middle SDI
53595	Davao Oriental	0.547236917	Low-middle SDI
53596	Davao de Oro	0.533098715	Low-middle SDI
53597	Davao Del Sur	0.659917629	Middle SDI

Location ID	Location Name	2021 SDI Index Value	SDI Quintile
53598	Davao Occidental	0.578314908	Low-middle SDI
53599	Davao Del Norte	0.636970822	Middle SDI
53600	South Cotabato	0.632536869	Middle SDI
53601	Sultan Kudarat	0.519757294	Low-middle SDI
53602	Cotabato (North Cotabato)	0.55211245	Low-middle SDI
53603	Sarangani	0.582418699	Low-middle SDI
53604	Agusan Del Norte	0.615707618	Low-middle SDI
53605	Agusan Del Sur	0.541666497	Low-middle SDI
53606	Surigao Del Sur	0.587486668	Low-middle SDI
53607	Surigao Del Norte	0.627545678	Middle SDI
53608	Dinagat Islands	0.622041414	Middle SDI
53609	Tawi-Tawi	0.535621341	Low-middle SDI
53610	Basilan	0.546108643	Low-middle SDI
53611	Sulu	0.48399862	Low-middle SDI
53612	Maguindanao	0.51070243	Low-middle SDI
53613	Lanao Del Sur	0.532909532	Low-middle SDI

Location ID	Location Name	2021 SDI Index Value	SDI Quintile
53614	National Capital Region	0.751536473	High-middle SDI
53615	Azad Jammu & Kashmir	0.541342775	Low-middle SDI
53616	Balochistan	0.417109886	Low SDI
53617	Gilgit-Baltistan	0.399312068	Low SDI
53618	Islamabad Capital Territory	0.695559154	Middle SDI
53619	Khyber Pakhtunkhwa	0.451366327	Low SDI
53620	Punjab	0.520053339	Low-middle SDI
53621	Sindh	0.513737094	Low-middle SDI
53660	Dolnośląskie	0.817783922	High SDI
53661	Kujawsko-Pomorskie	0.790185071	High-middle SDI
53662	Łódzkie	0.805693677	High-middle SDI
53663	Lubelskie	0.789673817	High-middle SDI
53664	Lubuskie	0.791264292	High-middle SDI
53665	Małopolskie	0.812434112	High SDI
53666	Mazowieckie	0.849859302	High SDI
53667	Opolskie	0.794191353	High-middle SDI

Location ID	Location Name	2021 SDI Index Value	SDI Quintile
53668	Podkarpackie	0.792282338	High-middle SDI
53669	Podlaskie	0.786166754	High-middle SDI
53670	Pomorskie	0.804282452	High-middle SDI
53671	Śląskie	0.81907325	High SDI
53672	Świętokrzyskie	0.796464119	High-middle SDI
53673	Warmińsko-Mazurskie	0.777449817	High-middle SDI
53674	Wielkopolskie	0.813257407	High SDI
53675	Zachodniopomorskie	0.792789122	High-middle SDI
60132	Vestland	0.917642172	High SDI
60133	Agder	0.907093137	High SDI
60134	Vestfold og Telemark	0.907351769	High SDI
60135	Innlandet	0.899977235	High SDI
60136	Viken	0.914602128	High SDI
60137	Troms og Finnmark	0.904453583	High SDI
